# Controlled synthesis and characterization of porous silicon nanoparticles for dynamic nuclear polarization†

**DOI:** 10.1039/d4nr02603a

**Published:** 2024-09-18

**Authors:** Gevin von Witte, Aaron Himmler, Viivi Hyppönen, Jiri Jäntti, Mohammed M. Albannay, Jani O. Moilanen, Matthias Ernst, Vesa-Pekka Lehto, Joakim Riikonen, Sebastian Kozerke, Mikko I. Kettunen, Konstantin Tamarov

**Affiliations:** a Institute for Biomedical Engineering, University and ETH Zurich Zurich Switzerland; b Institute of Molecular Physical Science, ETH Zurich Zurich Switzerland; c Kuopio Biomedical Imaging Unit, A.I. Virtanen Institute, University of Eastern Finland Kuopio Finland; d Department of Technical Physics, University of Eastern Finland Kuopio Finland konstantin.tamarov@uef.fi; e Department of Chemistry, Nanoscience Center, University of Jyväskylä Jyväskylä Finland

## Abstract

Si nanoparticles (NPs) have been actively developed as a hyperpolarized magnetic resonance imaging (MRI) contrast agent with an imaging window close to one hour. However, the progress in the development of NPs has been hampered by the incomplete understanding of their structural properties that correspond to efficient hyperpolarization buildup and long polarization decays. In this work we study dynamic nuclear polarization (DNP) of single crystal porous Si (PSi) NPs with defined doping densities ranging from nominally undoped to highly doped with boron or phosphorus. To develop such PSi NPs we perform low-load metal-assisted catalytic etching for electronic grade Si powder followed by thermal oxidation to form the dangling bonds in the Si/SiO_2_ interface, the *P*_b_ centers. *P*_b_ centers are the endogenous source of the unpaired electron spins necessary for DNP. The controlled fabrication and oxidation procedures allow us to thoroughly investigate the impact of the magnetic field, temperature and doping on the DNP process. We argue that the buildup and decay rate constants are independent of size of Si crystals between approximately 10 and 60 nm. Instead, the rates are limited by the polarization transfer across the nuclear spin diffusion barrier determined by the large hyperfine shift of the central ^29^Si nuclei of the *P*_b_ centers. The size-independent rates are then weakly affected by the doping degree for low and moderately doped Si although slight doping is required to achieve the highest polarization. Thus, we find the room temperature relaxation of low boron doped PSi NPs reaching 75 ± 3 minutes and nuclear polarization levels exceeding ∼6% when polarized at 6.7 T and 1.4 K. Our study thus establishes solid grounds for further development of Si NPs as hyperpolarized contrast agents.

## Abstract 2

Investigating the dynamic nuclear polarization mechanism in controllably synthesized porous Si nanoparticles, we measure the longest relaxation time to date.

## Graphical abstract

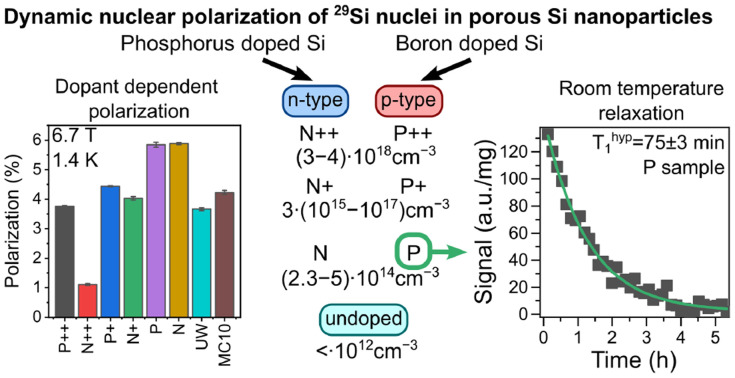

## 1 Introduction

Magnetic resonance imaging (MRI) is a non-invasive versatile technique that can provide anatomical images with either sub-millimeter spatial^1^ or milliseconds temporal^2^ resolution. Applying recent advances in artificial intelligence based image reconstruction and enhancement methods,^3^ low field MRI has recently reached real world adoption even in the mobile setting.^4^ MRI, however, is inherently insensitive at room temperature due to low thermal polarization of nuclei, which complicates the observation of nuclei other than ^1^H. Detecting low-abundant nuclei, such as ^13^C, ^15^N or ^29^Si, brings additional versatility to MRI allowing to *e.g.*, image tumor metabolism,^5^ locally detect pH,^6,7^ detect Si particles *in vivo* during prolonged time window.^8–10^ Porous Si nanoparticles (NPs) hold particular promise due to their biocompatibility and numerous treatment modalities.^11^

To detect Si NPs in an MRI scanner, their ^29^Si nuclei require hyperpolarization *i.e.*, a polarization significantly beyond thermal equilibrium at body temperature. A mature method to hyperpolarize various nuclei in the solid state is dynamic nuclear polarization (DNP).^12^ DNP requires the presence of polarized unpaired electronic spins, whose polarization is subsequently transferred to hyperfine (HF) coupled nuclei by (near-) resonant microwave (MW) irradiation.

In Si, the unbound electrons required for DNP can originate from substitutional donor dopant atoms, such as group V (P, As, Sb, Bi) or group VI (S) atoms, which carry one or more donor electrons. As each dopant carries extra electron(s), the majority carriers are negatively charged electrons and Si is named n-type. Spins of electrons bound to ^31^P donors have been widely used to polarize ^29^Si nuclear spins and to study polarization dynamics in bulk Si samples with different ^29^Si and ^31^P content.^13–16^ With the variation of ^31^P and ^29^Si content, well resolved solid effect (SE),^13,15,16^ differential SE^13^ and Overhauser effect (OE)^14–16^ DNP mechanisms of ^29^Si hyperpolarization have been identified. More sophisticated protocols, such as resonant polarization transfer from polarized ^31^P to ^29^Si nuclei,^17^ have been demonstrated.

If Si is doped with group III atoms, in particular boron, each dopant atom binds an electron leaving a hole in the valence band. The majority carriers are the positively charged holes and the Si is called p-type. Hole states in the valence band from the p-orbitals as opposed to the s-orbitals of electrons in the conduction band. The need to satisfy the 3-fold degeneracy of the p-orbital results in the splitting of the valence band into heavy and light hole bands.^18^ The degeneracy of these bands combined with the dopant atom-induced local random stresses broadens the electron paramagnetic resonance (EPR) spectrum making it hard to observe in B-doped Si unless uniaxial strain is applied.^19–21^ Strained single crystal Si : B has been used to study the integrated solid effect.^20^

Another source of electron spins are defect sites found in amorphous Si,^22^ oxidized Si surfaces^23–25^ and elemental Si particles.^8,9,26^ Such defect sites are characterized by a broken Si bond with an unpaired electron mostly localized on the central Si atom.^25^ When the defect is located at the Si/SiO_2_ interface, it is called the *P*_b_ center.^23–25^*P*_b_ centers and *P*_b_-like centers in amorphous Si have been used to hyperpolarize various Si particles and apply them as background-free contrast agents for MRI.^8,27^ The long hyperpolarization decay times (*τ*_dec_) of Si particles around ∼40 min at room temperature offered extended imaging time windows compared to about 30 s in ^13^C molecules^6^ or 145 s (15 min) in nanodiamonds (microdiamonds).^28^ In diamonds, the substitutional nitrogen defects in the particle's bulk (often called C or P1 center) are responsible for DNP while surface dangling bonds commonly cause strong relaxation. The surface dangling bonds thus are detrimental for nanodiamonds with high surface-to-bulk ratio leading to lower polarization levels and faster relaxation compared to microdiamonds.^29^ This is different from the case in Si with the *P*_b_ centers located on the interface to the naturally forming surface oxide which allows the hyperpolarization of 50 nm particles with identical enhancements compared to μm-sized particles.^26^

Despite the demonstrated high nuclear polarization and long nuclear *τ*_dec_ relaxation times at room temperature^26,30^ in bulk Si particles, the proposed slow spin diffusion fails to explain the similar *τ*_dec_ in micro- and nanoparticles. The diversity of fabrication methods further complicates the identification of the structural properties, their comparison between different particles and influence on *τ*_dec_. In this study, we apply a top-down fabrication approach^31,32^ to produce porous silicon nanoparticles (PSi NPs, sometimes denoted as nanobeads) with a high surface area from doping controlled, single crystal Si wafers. The role of the high surface area is twofold. First, it enables the controlled formation of a relatively large number of endogenous *P*_b_ centers to drive the DNP process. To the best of our knowledge, previous attempts to hyperpolarize PSi NPs required the use of external radicals for DNP to be efficient^10^ complicating possible MRI applications of those NPs. Second, the large surface area combines good biocompatibility with a well understood diverse chemistry for (targeted) nanocarrier capabilities^11^ making the developed PSi NPs suitable both for imaging and drug delivery.^11^ Herein, we prove that endogenous *P*_b_ centers in PSi NPs are capable of providing DNP enhancements similar to state-of-the-art bulk particles.^26^ Furthermore, we demonstrate that PSi nanoparticles from slightly doped Si wafers can achieve room temperature hyperpolarization decay times exceeding one hour and ^29^Si polarization levels around 6%.

## 2 Methods

### 2.1 Silicon

Previous studies on the DNP of Si NPs relied on either commercially available^9,26,30,37^ or on in-house bottom-up fabrication approaches.^10,30,38,39^ In contrast, we selected single crystal Si wafers as the starting material to precisely control crystallinity and doping level (Table 1). Specifically, we used electronics grade single crystal (100) silicon wafers of different doping (Okmetic Oy, Finland). The samples were denoted according to the doping type and doping density. Doping type was indicated by P (positive) and N (negative) letters for boron and phosphorus doping, respectively. The doping density ranged from 4 × 10^18^ cm^−3^ for P++ and N++ porous Si (PSi) NPs down to less than 10^12^ cm^−3^ for the nominally undoped wafer (UW). The doping density was below the insulator-to-metal transition for all Si wafers considered. In addition to wafers, we prepared PSi NPs from a relatively cheaper commercially available polycrystalline (1–10) μm Silgrain Supreme MC10 SB powder (Elkem Silicon Products, Norway) with known concentration of impurities (MC10 sample): the purity of the powder was 99.997% determined by the supplier, where the main impurities were Fe (14 ppm), Al (6 ppm), Ca (3 ppm), Ti (1 ppm), B (<1 ppm), and P (<1 ppm).

**Table 1:** Summary of Si grade abbreviations used to fabricate PSi NPs

Abbreviation	Resistivity (Ω cm)	Dopant	Doping density^a^ (cm^−3^)	Average dopant distance^b^ (nm)
P++^c^	0.0186	Boron	4 × 10^18^	3.49
P+^c^	0.105	Boron	3 × 10^17^	8.27
P^c^	25	Boron	5 × 10^14^	69.8
UW^c^	>5000	Boron	<10^12^	>554
N^c^	19.7	Phosphorus	2.3 × 10^14^	90.4
N+^c^	1.15	Phosphorus	3 × 10^15^	38.4
N++^c^	0.0144	Phosphorus	3 × 10^18^	3.84
MC10^d^	Metallurgical grade powder, polycrystalline, 99.997% purity. Impurities: Al, Fe, Ca, Ti			

a Dopant densities were calculated using Caughey–Thomas expression^33^ for electron and hole mobilities. Effective Bohr radii are 1.3 (3.8) and 2.1 nm for heavy (light) holes and electrons in B doped and P doped Si, respectively. The effective Bohr radius of the P electron assumes the pancake-like wavefunction ansatz proposed by Kohn and Luttinger.^34^

b Average distance between the dopant atoms was calculated from their density using the random probability distribution in three dimensions.^36^

c Powder from single crystal (100) wafers, Okmetic.

d Elkem Silicon Products.

Dopant type of the Si wafers was verified by hot point probe method.^40^ Specific resistivity was calculated using a MATLAB (The MathWorks, Inc., USA) script using wafer thickness and resistivity measured with a four-point probe (Jandel Engineering Ltd, UK) connected to a Cropico DO5000 microhmmeter (Seaward Electronics Ltd, UK).^40^ The dopant concentrations were estimated by comparing the measured specific resistivities with the ones calculated using Caughey–Thomas expression^33^ from electron and hole mobilities at 300 K assuming full ionization of dopant atoms. The average distances between dopant atoms were calculated from the doping densities using the probability density function to find the atom at a distance *r*.^36^ Assuming the uniform random distribution of the dopant atoms, the average distance is 〈*r*〉 ≈ 0.554·*N*_c_^−⅓^, where *N*_c_ is the density of atoms. The equivalent Bohr radii for acceptors were estimated using the expression derived from the hydrogen atom-like model of donor (acceptor): *a*_A_ = *ε*_r_*m*_0_/*m*_eff_·*a*_0_, where *a*_0_ is the Bohr radius of hydrogen atom, *ε*_r_ is the relative dielectric permittivity of Si, *m*_0_ is the electron mass, and *m*_eff_ is the effective mass of a hole. For the donors, *a* more precise value of the electron's effective Bohr radius is given by the geometric mean *a*_D_ = *a*^⅓^_∥_*a*^⅔^_⊥_ ≈ 2.087 nm, where *a*_∥_ ≈ 1.44 nm and *a*_⊥_ ≈ 2.51 nm are the two radii of the pancake-like wavefunction ansatz for the electron ground state proposed by Kohn and Luttinger.^34,35^ The information about Si types and abbreviations used in the text are summarized in Table 1.

### 2.2 Porous Si powders

(10–25) μm powders were prepared by ball-milling Si wafers using Fritsch Pulverisette 7 Premium Line (Fritsch GmbH, Germany). Obtained powders were washed in 3 wt% aqueous H_2_O_2_ by sonicating them for 1 h in an ultrasound bath.^32^ Such washing removes possible surface contaminations and ensures reproducibility. The powders were then dried and used to produce porous Si by low-load metal-assisted catalytic etching (LL-MACE) as reported before.^31,32^ The protocol was scaled up to perform etching of 2 g Si powder batches. Briefly, 2 g of Si powder was first dispersed in 30 ml of acetic acid (Ph. Eur., VWR Chemicals) inside of a 50 ml PTFE dish by 5 min sonication. Then, 30 ml of hydrogen fluoride (HF, 30–40%, Merck) was added, and the dish was placed in a water bath on a heat plate under stirring. Next, Au NPs were nucleated on Si powder surfaces using a syringe pump injection of 8.334 ml (= 50 μmol) of 0.006 M Au^3+^ ion solution, which was prepared by dissolving HAuCl_4_·3H_2_O (99.99%, Alfa Aesar, Thermo Fisher GmbH) in water. Injection rate was 333.3 μl min^−1^; after it was completed, Si powder suspension was stirred for 5 min more to complete the nucleation of Au^3+^ NPs. The temperature of the water bath was kept at about 39 °C to retain the temperature of the suspension in the range of (51–53) °C during etching. The etching was performed by injecting H_2_O_2_/H_2_O solution using the syringe pump at a rate of 133.3 μl min^−1^ (injection time equals to 90 min). The H_2_O_2_ volume (35 wt%, Acros Organics, Thermo Fisher GmbH) in the solution was selected to match the H_2_O_2_/Si molar ratio of 1.03. The open end of the plastic tube going from the syringe was immersed into the suspension with Si particles.

After the etching finished, porous Si particles were washed in Büchner-style funnel on a 55 mm diameter Grade 2 WhatmanRRR filter. After the etching solution was removed, porous Si particles were washed three times on the filter using deionized water. Next, about 10 ml of *n*-pentane (≥99%, VWR Chemicals) was poured on the filter with porous powder and it was allowed to dry for a few min under the fume hood. *N*-Pentane reduced the surface tension of water inside the pores and prevented the collapse of porous structure during the final drying which was completed overnight in an oven at 65 °C. Obtained microscale PSi powders were then stored in glass vials.

### 2.3 Surface oxidation and preparation of nanoparticles

After LL-MACE surfaces of PSi powders were hydrogen terminated (Fig. S3 and S4, ESI†). Localized electronic defects (*P*_b_ centers) formed at the Si/SiO_2_ interface during thermal oxidation of PSi particles. This approach gives the highest number of *P*_b_ defects among other methods.^41^ Thermal oxidation was done for 2 hours in NaberTherm R50/500/12 tube furnace (Nabertherm GmbH) at 345 °C in air.^41^

**Fig. 1:**
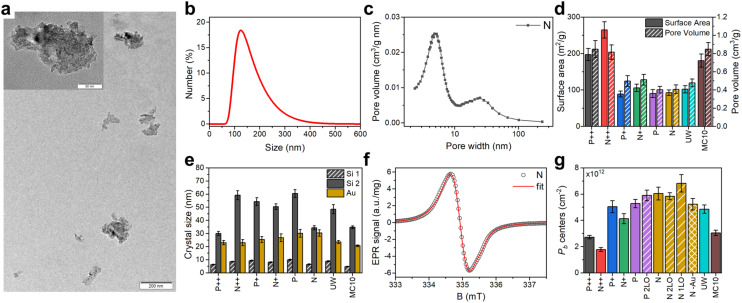
Characterization of PSi NPs. (a) Typical transmission electron microscopy image of PSi NPs dried out of suspension; the inset shows the high magnification view. (b) Hydrodynamic size distribution of the N PSi NPs in water suspension. (c) Pore size distribution of N Si powder after LL-MACE. (d) Specific surface areas and pore volumes obtained from N_2_ sorption measurements of Si powders after LL-MACE. (e) Crystalline sizes of Au NPs and pore sizes in PSi NPs calculated from X-ray powder diffraction spectra. (f) Electron paramagnetic resonance spectrum of N PSi NPs. The experimental data (black circles) was fitted (red lines) using trigonal *P*^(111)^_b_ and isotropic *P*^iso^_b_ centers (details see text and section S2.4, ESI†). (g) *P*_b_ defect density of PSi NPs formed by thermal oxidation (no label), thermal and two-step liquid-phase oxidation (2LO label), thermal and one-step liquid-phase oxidation (1LO label), and oxidation induced by Au dissolving solution (–Au label).

Thermally oxidized PSi powders were then milled into NPs using a dedicated system.^42^ About 400 mg of a PSi powder was placed into a 4 ml glass vial which was subsequently filled with 1 mm ZrO_2_ milling balls. The milling was then performed in 5 min cycles at 900 rpm to avoid overheating and leaks. After each cycle, pressure was released from the vials. Typically, 10 cycles were enough to obtain PSi NPs with most of the particles below 200 nm in hydrodynamic diameter (Fig. 1b and S1, ESI†).

In addition to thermal oxidation, two-step liquid-phase oxidation (oxidation in H_2_O_2_/NH_4_OH solution followed by oxidation in H_2_O_2_/HCl solution),^41^ or one-step (only H_2_O_2_/HCl solution) was performed for thermally oxidized PSi NPs (*i.e.*, after milling of thermally oxidized PSi powders, details in ESI†). Liquid-phase oxidation removed the remaining hydrogen in −O_*y*_SiH_*x*_ groups (Fig. S3 and S4, ESI†) as well as induced additional backbond oxidation. Liquid-phase oxidation was tested because it would be needed in future surface modification with PEG molecules to prolong the systemic circulation time and enabling the use of the PSi NPs *e.g.*, as MR imaging agents.^43^

### 2.4 Au removal

The absence of Au NPs influence on the DNP was confirmed with the N sample. Au NPs were dissolved using the KI/I_2_ gold etchant for the porous Si powder after LL-MACE. Gold etchant solution was prepared by dissolving 6.08 g of KI and 1.51 g of I_2_ in 30 ml of 5 M HCl. Use of HCl as solvent demonstrated better Au dissolution compared to water. To dissolve Au NPs, about 250 mg of N powder after LL-MACE was dispersed in 3 ml of ethanol to wet the hydrophobic surfaces. Then, 15 ml of gold etchant was slowly added to the Si powder suspension. Particles were then stirred for 2 h at 75 °C. Au amount before and after the dissolution was measured using a home build portable XRF setup^44^ and calculated using the calibration standards prepared with Au deposition step of LL-MACE. Finally, particles were washed 3 times with water in a Büchner-style funnel, wetted with *n*-pentane and dried in an oven as above. Then the powder was milled to NPs and denoted as N–Au.

### 2.5 Characterization

Morphology of PSi NPs was examined by transmission electron microscopy (JEOL JEM-2100F, JEOL Ltd). A 2.5 μl drop of suspension diluted in ethanol to a concentration of 20 μg ml^−1^ was dried on 400 mesh Cu holey carbon grid (Agar Scientific Ltd) and the grid was examined in the instrument. Hydrodynamic sizes of PSi NP were measured using dynamic light scattering (Zetasizer Nano ZS, Malvern Panalytical Ltd) after redispersion in water at 0.1 mg ml^−1^ concentration.

Specific surface area, specific pore volume and pore size distributions of PSi powders after LL-MACE were characterized by N_2_ sorption (Tristar II, Micromeritics Instrument Corp.). Specific surface areas were calculated from the linear part of adsorption isotherm using Brunauer–Emmett–Teller theory. Specific pore volumes were obtained from the total adsorbed amount at relative pressure of 0.97. Pore size distributions were calculated from desorption isotherm using Barrett–Joyner–Halenda model.

Pore sizes and sizes of catalytic Au NPs were further measured with X-ray powder diffraction (XRD, D8 Discover, Bruker Corp.). Powders were placed on a zero-background holder and scanned in (25–60)° two-theta angle range with step size of 0.0057° and time per step of 0.205 s. Crystalline sizes of two Si phases and one Au phase were then calculated with Rietvield refinement method using TOPAS® 4.6 software (section S2.3, ESI†). The sizes calculated from the Si peak broadenings corresponded to the two pore sizes according to the Babinet's principle in single crystals.^45^

Surface chemical species and *P*_b_ centers formed by oxidation were studied with Fourier-transform infrared spectroscopy (FTIR, Thermo Nicolet iS50, ThermoFisher Scientific Corp.) and electron paramagnetic resonance spectroscopy (EPR, Magnettech MiniScope MS5000, Bruker Corp.). In FTIR, KBr tablets with dried PSi NPs were measured in transmission mode (Sec. S2.2, ESI†). For EPR measurements, the first 7 mm of an EPR tube were filled with dried PSi NP powder. The tube was placed in the spectrometer at the same height for each measurement with the following parameters: (1) *B*_0_ = 336 mT, *B*^scan^_0_ = 15.5 mT, *B*^modulation^_0_ = 0.2 mT, *t*_scan_ = 60 s, MW attenuation 25 dB and gain 10 for the full spectra; (2) *B*_0_ = 336 mT, *B*^scan^_0_ = 35 mT, *B*^modulation^_0_ = 0.7 mT, *t*_scan_ = 60 s averaged 3 times, MW attenuation 15 dB and gain 500 to resolve hyperfine peaks. To calculate the concentration of *P*_b_ centers and the *g*-factor, a standard 2,2,6,6-tetramethylpiperidinyloxyl (TEMPO) radical (99%, Sigma-Aldrich) sample with known number of paramagnetic centers and *g*-factors was used. EPR spectra were fitted using EasySpin 5.2.35 by simulating solid-state continuous-wave powder spectra using a combination of anisotropic *P*^(111)^_b_ and isotropic *P*^iso^_b_ centers. *g*-Factor strain, hyperfine coupling and Voigtian line broadening were included (section S2.4, ESI†).^46^

### 2.6 Dynamic nuclear polarization

Hyperpolarization of PSi NPs was studied using three different polarizer designs: SpinAligner (Polarize ApS) operating at 3.35 T or 6.7 T and a base temperature of 1.4 K as well as with two home-built setups with 3.34 and 7 T (ref. 47–49) with both operating at a base temperature of 3.4 K. About 100 mg of dried PSi NP powder was packed into a polymer sample container for measurements with the SpinAligner compared to (50–60) mg for the home-built set-ups. Microwave radiation was delivered through a waveguide elbow to directly irradiate the sample. The microwave irradiation^9,26^ was frequency modulated in all polarizers. Magnetic field strength, temperature, microwave power *W*, frequency modulation bandwidth Δ*ν*_MW_ and frequency of modulation *ν*_MW_ are summarized in Table 2.

**Table 2:** Summary of the DNP conditions

*B*_0_, T	*T*, K	*W* a, mW	Δ*ν*_FM_ b, MHz	*ν*_FM_ c, kHz
3.34	3.4	200	∼150	1
3.35	1.4	80	100	1
6.7	1.4	30	200	3
7	3.4	200^d^	300	10

a Microwave power.

b Frequency modulation bandwidth.

c Frequency of the modulation.

d Silver-plating the waveguide approximately doubled the MW power reaching the sample^49^ for the nominal 200 mW output of the source.

To monitor the ^29^Si signal, a flip angle of ∼2.8° was used in the SpinAligner with varied time intervals between the measurements. Flip angles of ∼1.5° each 20 min at 3.34 T and ∼6.9° every 6 to 10 min at 7 T were used. Obtained data was analyzed using MATLAB scripts, where either the real part of the time-domain free induction decay (FID) was fitted with an exponential ansatz or the real part after fast Fourier transform (FFT) in the frequency-domain was fitted with pseudo-Voigt functions. Polarization enhancements and absolute polarization values were calculated from the thermal equilibrium signal taken in the hyperpolarization conditions after 72 h of polarization with microwave irradiation switched off for the 6.7 T (1.4 K) measurements (section S3.1, ESI†). For the 3.34 and 7 T (3.4 K) measurements, the thermal equilibrium signal at 300 K of a fully ^29^Si isotope labeled sample (Isoflex, Russia) was measured and adjusted for temperature upon calculation of enhancements and absolute polarization in the DNP experiments. Both the polarization buildup data and the relaxation data was corrected for the perturbations by the monitoring RF pulses^50^ (except for the 3.34 T due to the small flip angle used and difficulties in measuring such small flip angles with high relative accuracy).

### 2.7 Correlation analysis

Correlation analysis was performed using the Matlab corrcoef() function. The data supplied to the function consisted of sample characterization data (Fig. 1), EPR data obtained from fitting (Tables S1 and S2, ESI†), DNP data obtained from fitting and the rate-equation model (Fig. 3 and S26, ESI†).

## 3 Results

The applied fabrication procedure (Methods section) results in irregular shaped PSi NPs with average particle sizes of (150 ± 65) nm (Fig. 1a and b). Additional milling and centrifugal selection could further reduce particle sizes if required for a specific (biological or medical) application (section S2.1, ESI†). The porous structure with two distinct pore sizes was formed during the Au-catalyzed LL-MACE (Fig. 1c). Etch track pores (>10 nm) were produced by Au NPs boring into Si, while tortuous pores (<10 nm) were formed by hole escape from space-charge layers to distant Si surfaces.^31,32^ This porosity resulted in a high surface area and a high number of surface *P*_b_ centers after oxidation (Fig. 1d and g). X-ray powder diffraction (Fig. 1e and section S5, ESI†) showed Si peaks with distinct superimposed peak profiles. Typically, the peak broadening of small crystals is dependent on the crystallite size but the porous nature of the PSi NPs complicates the picture. Since the Si particles are single crystals before etching (except for the MC10 sample) and preserve the crystallinity during the etching, the pores also give a contribution to the peak broadening according to the Babinet's principlecite.^45^ Therefore, three distinct contributions to the peak broadening would be expected for the PSi NPs caused by the crystallite size, population of wide etch track pores and the population of narrow tortuous pores. It was not possible to reliably fit the data with three peak profiles but instead fit with two profiles was done. The narrower peak profiles ((30–60) nm bars) were attributed to the etch track pores and the broadening from the small crystal size, while the wider ones ((5–10) nm bars) were due to tortuous pores penetrating the large crystals (Fig. 1e).^31,32^ The wide and narrow XRD peaks were on the order of the corresponding pore sizes measured by N_2_ sorption and depicted in Fig. 1c.

The subsequent thermal oxidation (Methods and section S2.2, ESI†) stabilized the H-terminated surface of freshly etched samples simultaneously making them hydrophilic. The created core–shell structure of PSi NPs thus consisted of the crystalline cores of pore walls (bulk) with a thin oxide shell (surface). The lattice constant mismatch between the Si and SiO_2_ led to the formation of paramagnetic centers in the Si/SiO_2_ interface.

Electron paramagnetic resonance (EPR) spectra (Fig. 1f, discussion of *P*_b_ centers below and section S2.4, ESI†) showed the presence of two typical paramagnetic centers found on oxidized (porous) Si surfaces: (i) trigonal *P*^(111)^_b_ centers with axial symmetry similar to defects found in oxidized planar (111) and porous Si surfaces (*g*_∥_ = 2.00185, *g*_⊥_ = 2.0081)^25,51,52^ and (ii) isotropic *P*^iso^_b_ defects commonly observed in oxidized porous Si (*g* = 2.0055).^51,53–57^ EasySpin^58^ was used to simulate the experimental EPR spectra to obtain the relative weights of the *P*^(111)^_b_ and *P*^iso^_b_ centers in our samples (section S2.4, ESI†). The simulations gave typical weights of (10–20)% for the *P*^(111)^_b_ and (80–90)% for the *P*^iso^_b_ defects. It was expected that *P*^iso^_b_ is the dominant defect center due to the random nature of pore formation in LL-MACE and thermal oxidation in air. Hyperfine (HF) interaction with the central ^29^Si was also observed (section S2.4, ESI†) and measured to be in the range of *A* = (325–431) MHz, which coincided well with *A*_∥_ = 210 MHz and *A*_⊥_ = 417 MHz for the planar *P*^(111)^_b_ center.^52^ The number of all types of *P*_b_ centers per unit area and per mass varied between (1.8–6.8) × 10^12^ cm^−2^ (Fig. 1g) and (4.4–6.3) × 10^15^ mg^−1^, respectively (section S2.4, ESI†). These values corresponded to the fraction of total *P*_b_ centers per silicon interface atoms of *f* ≡ [*P*_b_]/*N*_a_ = (0.23–0.87)% (where *N*_a_ = 7.83 × 10^14^ cm^−2^ is the density of lattice sites in the (111) plane). The average distance between the *P*_b_ centers was then calculated from the concentration per unit area using the nearest neighbors distribution^36^ derived for the 2D case. The average distances varied between 1.9 nm (N 1LO PSi NPs) and 3.7 nm (N++ PSi NPs). Correspondingly, the maximum dipolar interaction between electron spins of *P*_b_ centers ranges from 1.0 to 7.4 MHz if a uniform surface distribution of *P*_b_ centers is assumed.

We performed DNP NMR hyperpolarization and relaxation studies at four different conditions (3.34 T and 7 T at 3.4 K, 3.35 T and 6.7 T at 1.4 K, Table 2) with only selected samples evaluated at all the experimental conditions. The measured DNP profiles followed the symmetry of the EPR spectrum with the positive and negative DNP lobes located at a similar distance to the central zero crossing of the DNP enhancement (section S3.3, ESI†). The zero crossing of the DNP enhancement coincided with the center of the EPR line in agreement with previous works with endogenous defects in Si.^26,30^

**Fig. 2:**
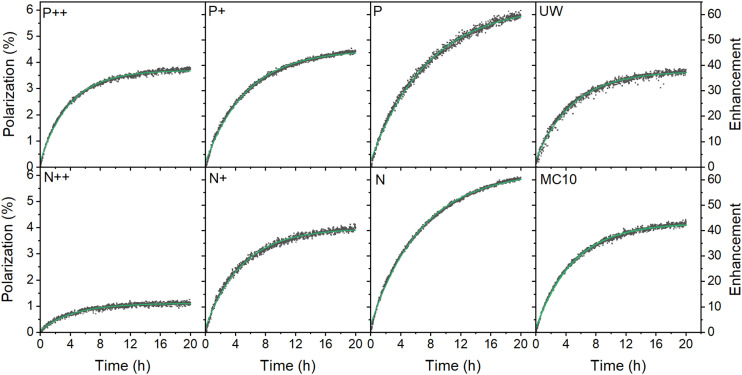
Dynamic nuclear polarization of thermally oxidized PSi NPs with different dopants after correcting for perturbation by the RF pulses^50^ (dark squares) and single exponential fit (green lines) at 6.7 T and 1.4 K. The microwave frequency was set to 187.82 GHz with a 150 MHz modulation bandwidth, 3 kHz modulation frequency and 30 mW microwave output power. The enhancement is relative to the thermal polarization of ^29^Si nuclear at the polarization buildup conditions. For the characteristics of the various samples, see Table 1.

^29^Si polarization buildup data at 6.7 T (1.4 K) for the thermally oxidized PSi NPs with various dopants are depicted in Fig. 2. The data was corrected for the perturbations by the monitoring RF pulses.^50^ We confirmed that the algorithm correctly recovered the genuine buildup dynamics from high sampling rate data in Fig. 2 using a low sampling rate of 30 min for the P sample (Fig. S17, ESI†). The one-compartment model underlying the RF correction assumes a mono-exponential buildup and decay dynamics^50^ as observed in all our samples and experimental conditions (section S3.3, ESI†).

The polarization buildup (at 6.7 T and 1.4 K) depended on the doping degree. The lowest polarization was found for the highly doped P++ and N++ samples but with significant difference between them despite the similar doping level of the starting Si powder (Table 1). With the decrease of doping density, the gained polarization levels tended to equalize between different doping types (P+ and N+ PSi NPs). Interestingly, the nominally undoped UW PSi NPs did not show the highest absolute ^29^Si polarization; the highest polarization levels were obtained for lightly doped P and N samples. Moreover, the relatively impure polycrystalline MC10 PSi NPs showed slightly better DNP polarization and similar buildup times than moderately doped P+ and N+ samples. Such polycrystalline grades could thus be a cheaper alternative to electronics grade sample with sufficiently good DNP properties.

**Fig. 3:**
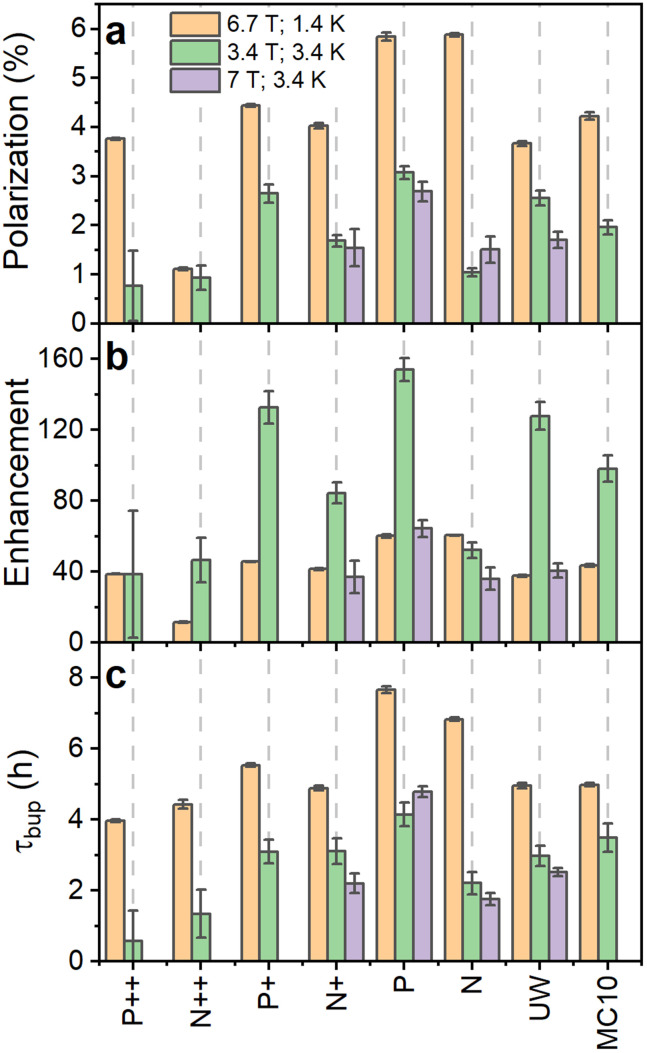
Comparison of ^29^Si nuclear polarization (a), enhancement over the thermal signal (b) and polarization buildup time (c) for PSi NPs at 6.7 T (1.4 K) (orange bars) as well as 3.34 T (3.4 K) (green bars) and 7 T (3.4 K) (violet bars). Temperature decrease or increase of magnetic field strength increase the thermal nuclear polarization used to calculate the enhancement from the nuclear polarization. The polarization, enhancement and buildup time are corrected for perturbations by the RF pulses.^50^ MW frequency modulation was employed in all the experiments.

The DNP characteristics changed significantly at 3.34 T and 3.4 K (Fig. 3 and S18, ESI†). The polarization buildup times (Fig. 3c) for all the samples almost halved compared to 6.7 T (1.4 K). The observed enhancements (Fig. 3b) were significantly higher at 3.34 T especially for the low B doped PSi NPs compared to the 6.7 T data. The n-type samples demonstrated only moderate enhancement increases with the N sample showing even lower enhancement than at 6.7 T. Despite the higher enhancements at 3.34 T, the estimated absolute ^29^Si polarization was still higher at 6.7 T (1.4 K) compared to 3.34 T (3.4 K) (Fig. 2, 3a and S18, ESI†) due to the higher thermal nuclear polarization.

In order to clarify the influence of the experimental conditions on DNP, we performed selected measurements at 7 T (3.4 K) to discriminate between field and temperature dependent changes (Fig. 3 and S19, ESI†). The decreased polarization for the N PSi NPs clearly followed the same trend as at 3.34 T while the absolute enhancement values and buildup times for P and UW samples were close to the 6.7 T data. The similarities for P and UW samples were even more striking provided the MW power was 30 mW at 6.7 T compared to 200 mW at 7 T. We then verified at 7 T (3.4 K) that 200 mW and 20 mW provided similar enhancements at 7 T making the comparison between 6.7 T and 7 T possible despite the large difference in MW power (Fig. S22, ESI†). We, therefore, conclude that temperature plays the crucial role in DNP performance of n-type PSi NPs, while it has less influence on the p-type samples. The temperature dependence for p-type samples was further investigated at 3.35 T (1.4 K) (Fig. S23, ESI†). We found a significant decrease of enhancement levels compared to the other conditions with minor differences between P and P++ PSi NPs.

**Fig. 4:**
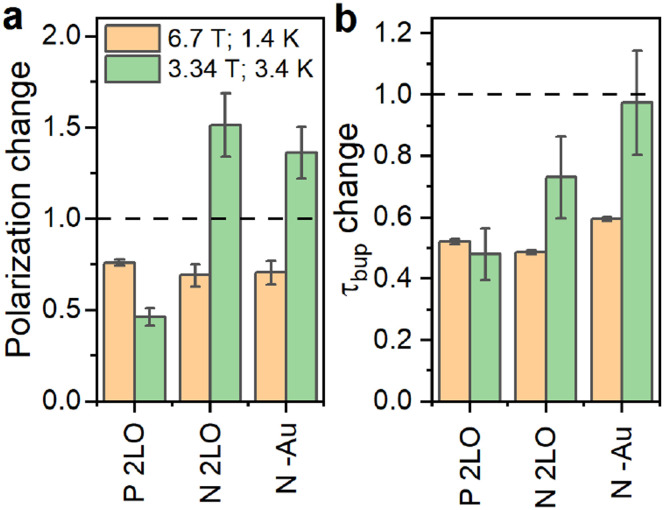
Relative change of the ^29^Si steady-state polarization (enhancement) (a) and polarization build up time (b) due to oxidations for P and N samples. The 2LO oxidation indicates the two-step liquid oxidation (section S1.1, ESI†) performed after the thermal oxidation. For the N–Au, oxidation emerged during the Au removal after LL-MACE (Experimental section). The dashed line indicates no change *i.e.*, the same measured value compared to thermally oxidized samples. The absolute values are in Fig. S21, ESI.†

In addition to the thermal oxidation used to create *P*_b_ centers on differently doped PSi NPs, we applied liquid oxidation^41^ to the P and N PSi NPs. Liquid oxidation reduced the number of surface hydrogen in –Si_*y*_H_*x*_-Si–H and –O_3_SiH surface groups (section S2.2, ESI†), which is an important step towards an improved surface coating for biomedical applications.^43^ We note that liquid oxidation affected the p- and n-type Si samples differently (section S2.2, ESI†). The same is true for the measurements with different DNP conditions (Fig. 4 and Fig. S18, S20, S21, ESI†): for the P sample, enhancement dropped significantly at 6.7 T (1.4 K) and 3.34 T (3.4 K). Contrary to the P sample, liquid oxidation of the N sample increased the enhancement about 1.4 times at 3.34 T (3.4 K), while at 6.7 T (1.4 K) the enhancement decreased. The polarization build up times were affected in a more consistent manner (Fig. 4b): for all the samples and liquid oxidations, the buildup times shortened to (0.5–0.7) times the buildup time of the thermally oxidized N or P PSi NPs. Future studies might explore the influence of oxidation, doping, and DNP conditions on the DNP *via P*_b_ centers further.

Finally, we verified that the presence of Au NPs left in PSi NPs after LL-MACE had little impact on DNP performance. For verification, we applied an iodine-based Au etchant to the N PSi NPs directly after LL-MACE (no thermal oxidation). The Au dissolution resulted in decrease of Au content from 0.37% for N PSi NPs to 0.02% for N–Au PSi NPs as measured by XRF. Since the Au etchant is a strong oxidative solution, the dissolution process also oxidized the PSi NP surfaces which are hydrogen terminated and hydrophobic after LL-MACE. For N PSi NPs, the etchant-induced oxidation had similar effects as liquid oxidation (Fig. 4).

After collecting the DNP data for the various samples at 3.34 T and 6.7 T, we selected the P, UW and N samples for room temperature relaxation measurements (Fig. S25, ESI†). For this, the samples were hyperpolarized at 3.34 T (3.4 K) for around 20 h and subsequently transferred (dry, tightly packed sample container) to the nearby temperature-controlled (300 K) 7 T setup. At room temperature, the differences between the decay times *τ*_dec_ of the selected samples diminished compared to liquid helium temperatures (Table S3, ESI†). Nevertheless, a smaller *τ*_dec_ for the N sample compared to the P and UW samples was observed. The hyperpolarized decay times at room temperatures of the P and UW samples were around 70 min (Table 3).

**Table 3:** Relaxation time of the selected PSi NPs at 7 T and room temperature after DNP at 3.34 T and 3.4 K

Abbreviation	*τ*_dec_, min
P	75 ± 3
UW	67 ± 8
N	52 ± 5

## 4 Discussion

**Fig. 5:**
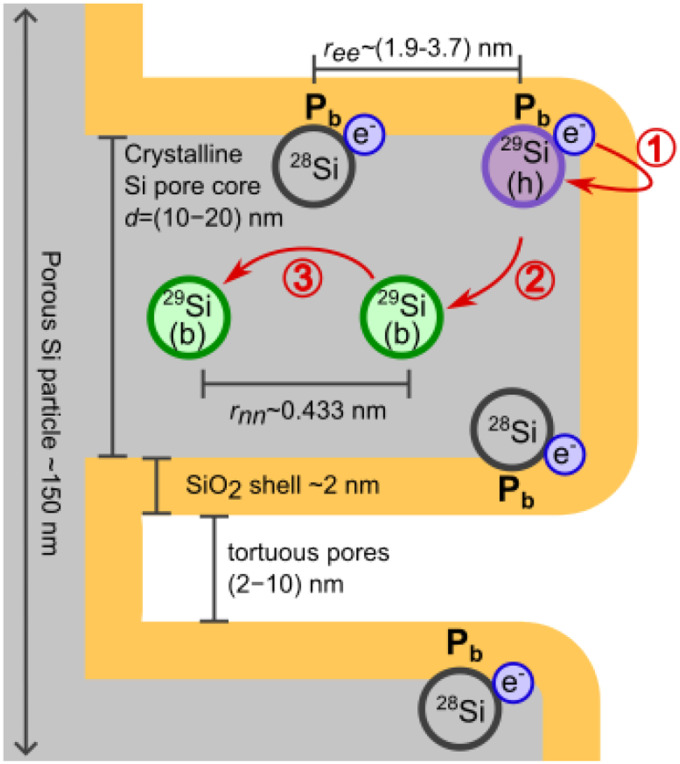
Sketch of the PSi NPs with ∼150 nm particle size and a large number of torturous pores (not to scale). *P*_b_ centers form at the interface between the surface oxide shell and the crystalline pore wall cores. Average electron–electron (*r*_ee_) and (^29^Si) nuclear-nuclear (*r*_nn_) distances for 4.7% natural abundance ^29^Si are indicated. The hyperpolarization pathway is indicated in red. The polarization is transferred from the electron to the hypershifted ^29^Si (h) nucleus of a *P*_b_ center (step 1) and from there to a nearby bulk (b), NMR visible ^29^Si spin (step 2). Within the crystalline pore wall core (step 3), the nuclear hyperpolarization is spread *via* nuclear spin diffusion. Only 4.7% of *P*_b_ centers have ^29^Si nucleus and, therefore, directly participate in DNP.

The following discussion is organised along Fig. 5, which sketches the relevant length scales and the proposed polarization pathway in the PSi NPs. The *P*_b_ centers at the interface between the surface oxide shell and the crystalline silicon core provide the unbound electrons required for DNP. Thus, understanding DNP in PSi NPs requires a basic understanding of *P*_b_ centers, which will be provided in section 4.1.

To achieve a hyperpolarized nuclear state, the high thermal electron polarization is transferred *via* hyperfine (HF) coupling to ^29^Si nuclei of a *P*_b_ center located on the interface between the bulk pore walls and oxide shell (step 1 of hyperpolarization buildup sketched in Fig. 5). The HF coupling shifts the resonance frequency of the *P*_b_ nuclear spins rendering it difficult to observe these spins with NMR (hypershifted spins^59^). The hypershifted spins have a resonance frequency (energy) discrepancy to the bulk nuclear spins in the pore walls (visible by NMR). The frequency discrepancy suppresses the nuclear spin diffusion between the hypershifted and bulk spins (step 2 in Fig. 5) making the step to be the time limiting as further argued below in section 4.2. Between the bulk ^29^Si in the pore wall cores, nuclear spin diffusion (nSD) spreads the nuclear hyperpolarization throughout the crystalline pore wall cores (step 3 in Fig. 5). The discussion of the different steps is then extended to the hyperpolarization decay in section 4.3. Finally, the effects of different samples and experimental conditions are discussed in section 4.4.

### 4.1 *P*_b_ centers

In section S2.5, ESI,† we concisely review existing literature on the interfacial *P*_b_ centers in Si/SiO_2_. Based on this review, the measured EPR spectra are fitted with two types of *P*_b_ centers: (i) a *P*^(111)^_b_ center with trigonal symmetry and (ii) *P*^iso^_b_ with spatially isotropic *g*-factor. If a *P*_b_*c*enter has a ^29^Si nucleus at its central site, it possesses a Fermi-contact (isotropic) hyperfine coupling of several hundred MHz (*A*_∥_ = 210 MHz; *A*_⊥_ = 417 MHz) to the unbound electron owing to the localized *P*_b_ electron's wavefunction. The next nearest neighboring ^29^Si atoms have hyperfine (HF) coupling of a few tens of MHz.^25^ Si nuclei further away from the *P*_b_ center are assumed to be mostly coupled *via* dipolar HF interactions to the electron and to other Si nuclei. In naturally abundant Si with 4.7% ^29^Si, only a fraction of *P*_b_ centers has a HF coupling exceeding tens of MHz and only 4.7% will have HF couplings exceeding hundreds of MHz.

The HF couplings of hundreds of MHz split the EPR line into three lines: a strong central EPR line of *P*_b_ centers with non-magnetic Si nuclei and weak HF doublet of the 4.7% of *P*_b_ centers with a ^29^Si nucleus at its central site. In our experiments, the doublet outer lines are shifted by roughly ±*A*_ave_/2 = ±(*A*_⊥_ + *A*_∥_)/4 ≈ ±162 MHz with respect to the central electron line in a good agreement with the literature (section S2.5†). Each of the three lines is anisotropically broadened due to *g*-factor strain in the irregular Si/SiO_2_ interface, which leads to a full electron line consisting of three connected EPR lines (*m*_I_ = −½, 0, ½). Taken together, the anisotropic line broadening provided by the HF interaction and the *g*-factor strain (together >300 MHz) is larger than the nuclear Larmor frequency *ω*_0n_ (between 28 and 60 MHz).

From the fitted *P*_b_ signal (Fig. 1d and Table S1, ESI†) and the measured surface area (Fig. 1d), the estimated average distance between the *P*_b_ centers assuming their uniform distribution is *r*_ee_ = (1.9–3.7) nm. This distance gives the estimated electron dipolar coupling *D*_ee_ on the order of *D*_ee_ = (1.0–7.4) MHz. The electron dipolar coupling is about (1–10) times lower than the homogeneous line broadening (Table S2, ESI†) which might indicate a non-uniform distribution and with that larger electronic couplings. The estimated electron dipolar coupling values *D*_ee_ are strong enough to induce mutual electron spin flip-flops within the EPR line.^60^

The summarized EPR data satisfies the three main conditions for the triple spin (2 electron spins and 1 nuclear spin) family of DNP mechanisms. First, the dipolar interaction is strong enough within the EPR line to result in electron–electron flip-flops. Second, the EPR line is broader than the nuclear Larmor frequency at all the experimental conditions. Third, part of the electron spins in *P*_b_ centers are HF coupled to ^29^Si nuclei. Following the ongoing theoretical efforts to understand triple spin flips in DNP,^60–66^ we refrain ourselves from going into the specific variants, such as cross effect or thermal mixing DNP. We highlight that *D*_ee_ values in our samples support cross effect DNP according to recent quantum mechanical simulations.^60^ Finally, we also note the results from previous study of nominally undoped Si microparticles, in which the decay of nuclear hyperpolarization was explained through triple spin flips,^67^ emphasizing the importance of triple spin flips in the Si/SiO_2_ interface.

### 4.2 Rate limiting step for the polarization buildup

To achieve the polarization levels up to a few percent, the polarization needs to penetrate from the surface nuclei into the pore wall cores of the PSi NPs for which we invoke the concept of nuclear spin diffusion (nSD).^12,68^ The dipolar interaction between nuclei induces nuclear spin flip-flops—a zero-quantum (ZQ) process with no net change of the total magnetic quantum number. This ZQ process causes an effective spatial transport of magnetization that can be described by a diffusion equation if a nuclear polarization gradient is present in the sample.

The nSD constant in Si was previously approximated with *D*_diff_ ≈ *a*^2^/(*cT*_2n_), where *c* = 30 ^13^ or *c* = 50,^69^*a* is the average distance between ^29^Si nuclei in a cubic lattice and *T*_2n_ is a measure for the inhomogeneous single quantum (SQ) line width in the spectra. In the approximation of *D*_diff_ in ref. 69, it was implicitly assumed that the experimentally measured SQ Hahn echo decay characterized by *T*′_2n_ ≈ 5.6 ms (ref. 70) characterizes also the width of the ZQ line, which is relevant for nSD. In ref. 13, the decay constant of the FID 
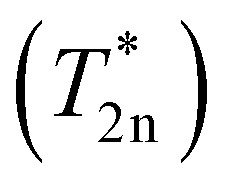
 was assumed to characterize the width of the ZQ line.^13^ We note that all of these decay time constants are not relaxation times in the strict sense of stochastic processes that lead to decoherence. Nonetheless, both approaches lead to similar nSD coefficients of *D*_diff_ ≈ 0.5–1.7 nm^2^ s^−1^. Therefore, for the polarization to diffuse from the surface into the pore wall cores *r*_wall_/2, a time scale of only ∼8 s or ∼140 s (*τ*_diff_ = 〈(*r*_wall_/2)^2^〉/6*D*_diff_) is required for the tortuous or etch track pores, respectively (Fig. 1c and e). These time scales are orders of magnitude shorter than the liquid helium build-up times of hours (Fig. 2 and section S3.3, ESI†) or the room temperature decay times of around one hour (Table 3 and Fig. S25, ESI†). Hence, we conclude that the nuclear spin diffusion (step 3 of the hyperpolarization buildup in Fig. 5) is not limiting the hyperpolarization process.

**Fig. 6:**
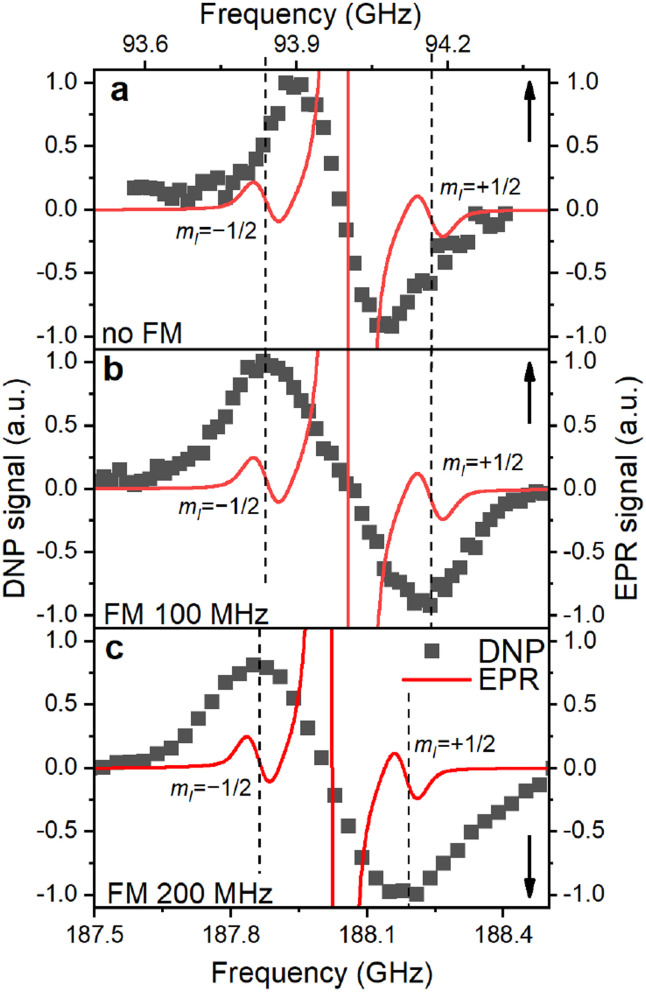
Overlay of the simulated EPR and experimental DNP spectra for the P sample at (a) 3.35 T, 1.4 K without MW modulation (frequency modulation – FM), (b) 3.35 T, 1.4 K with 100 MHz MW modulation and (c) 6.7 T, 1.4 K with 200 MHz MW modulation. The DNP and EPR spectra are normalized separately in each panel. EPR spectrum is the frequency-swept spectrum simulated using the model obtained from the experimental data fitting (Fig. 1f, section S2.4, ESI†). EPR spectrum consists of the central ^28^Si manifold (clipped) and two hyperfine-split manifolds for *P*_b_ centers with ^29^Si nuclei (*m*_I_ = ±1/2, dashed lines). The upwards and downwards arrows indicate the *X* axis for each graph.

The EPR spectrum extrapolated to the DNP field strength of 3.34 T or 6.7 T consists of three lines (Fig. 6): the central line for *P*_b_ with ^28^Si nuclei is surrounded by the two HF-split lines for *m*_I_ = ±1/2 whose shape is the same as of the central line. The DNP profiles show two DNP peaks of positive and negative enhancements with nearly equal amplitude and width (Fig. 6 and S13, ESI†). If MW modulation is applied, the extrema of the DNP enhancement in our samples coincide with the frequencies of the HF-split *m*_I_ = ±1/2 doublet in the EPR spectrum (Fig. 6b and c). Switching off MW modulation (Fig. 6a) narrows the DNP profile while retaining its featureless shape with its width far exceeding the nuclear Larmor frequency (*ω*_0n_ (3.34 T) ≈ 28 MHz).

In DNP, the strength of the HF interaction between electron and nuclear spins determines the polarization transfer rate constant, which is proportional to the square of the HF coupling matrix element. Owing to the large HF constant between the *P*_b_ electron and the central Si atom (*A*_∥_ = 210 MHz; *A*_⊥_ = 417 MHz), the DNP of these nuclei should be efficient and fast (step 1 of the hyperpolarization buildup in Fig. 5). Already for nearest neighbors (*A*_2n_ ≈ 42 MHz (ref. 25)) ^29^Si nuclei the roughly ten times lower HF coupling would lead to an approximately hundred-fold lower polarization transfer rate compared to the central ^29^Si, which outweighs the higher number of nearest neighbor lattice sites (between 1.5 and 3 depending on the location of a *P*_b_ center). MW modulation further improves the DNP likely through recruiting more electrons and shifts the positive and negative enhancements apart when applied^9,26,71^ as observed in Fig. 6a and b. Interestingly, we found the optimal MW modulation bandwidth to be 100 MHz and 200 MHz at 3.35 T and 6.7 T, respectively. These bandwidths make the maximum positive and negative DNP enhancements to coincide with the *m*_I_ = ±1/2 EPR manifolds. We interpret this coincidence as indication for the electron-nuclear polarization transfer pathway occurring preferentially through *P*_b_ centers with ^29^Si central nuclei and not through the backbonded nearest neighbor ^29^Si. The increased transfer efficiency to the central ^29^Si can be understood by the up to ten times larger HF coupling compared to other possible locations of ^29^Si and amplified by the polarization transfer rate scaling approximately with the HF coupling squared.

Taken together, both the polarization of the central *P*_b_^29^Si and the nuclear spin diffusion throughout pore walls (steps 1 and 3 of the hyperpolarization buildup in Fig. 5) are relatively fast processes compared to the measured buildup and decay times at all the DNP conditions (Fig. 2 and section S3.3, ESI†). In order to explain the long polarization buildup and decays we shall recall that there are ^29^Si nuclei with remarkably strong HF interaction—the central and backbonded *P*_b_ nuclei. Between these strongly hypershifted ^29^Si spins and the bulk spins exists a large shift in frequency/energy, which is further enhanced due to the sparsity of ^29^Si in the naturally abundant PSi NPs. Such frequency shifts suppress nuclear flip-flop transitions and create a so called spin diffusion barrier.^12,68^ The transfer from the hypershifted nuclear spins to the bulk ^29^Si is, therefore, restrained, making it the rate limiting step in the DNP buildup.

For the nuclear polarization to diffuse across the spin diffusion barrier, the electrons need to modify the effective nuclear-nuclear spin interactions.^72–80^ Specifically, a coherent electron-nuclear four-spin flip-flop process^79,80^ can be consistent with low temperatures employed in our experiments. The electron-nuclear four-spin flip-flops are very similar to triple spin flips involving an electronic flip-flop and nuclear spin flip but the nuclear spin flip is replaced by a nuclear dipolar flip-flop.^79^ The transition matrix element of the electron-nuclear four-spin flip-flops is proportional to the electronic and nuclear dipolar couplings. Thus, a higher nuclear isotope abundance increases the rate of electron-nuclear four-spin flip-flops by increasing the nuclear dipolar couplings (due to the decrease of the average internuclear distances). A higher electron-nuclear four-spin flip-flop rate leads to a faster spin transport from the hypershifted to bulk spins (step 2 in Fig. 5).

Another possible explanation for the long buildup time invokes the polarization transfer towards weakly HF-coupled spins. In this case, distant ^29^Si nuclei are polarized directly by the dipolar coupling to a *P*_b_ electron spin. The direct polarization transfer hypothesis, however, possess a few flaws. First, this process has a low probability since the HF coupling between a *P*_b_ electron and a distant ^29^Si nucleus rapidly vanishes with the distance between them. For example, for a nuclei located at a distance of three lattice constants, the HF interaction is ∼3.5 kHz, yielding low rates of direct polarization transfer. Such a low polarization transfer rate might be too slow for the observed buildup times and enhancements. Second, even a frequency difference of ∼3.5 kHz is large compared to natural abundance SQ NMR line width of around 100 Hz.^13^ Assuming that the SQ line is a good approximation for the ZQ line mediating nSD, the spectral density of energy conserving ZQ flip-flops vanishes when the ZQ line width is much smaller than the energy difference between the nuclei^81^ as in this case by the given HF couplings. Hence, nSD would still be suppressed by the HF couplings and would require an electron spin to alter the spin diffusion locally similar to the polarization transfer occurring at the strongly HF coupled nuclei.^72–80^ Third, the direct polarization transfer fails to explain the *m*_I_ = ±1/2 DNP enhancements and zero DNP for the central EPR peak (Fig. 6).

We highlight that 95.3% of the *P*_b_ centers have magnetically inactive ^28^Si or ^30^Si central nuclei. Therefore, in addition to the low probability of transfer from the central ^29^Si to the distanced bulk, only 4.7% of *P*_b_ contribute to the hyperpolarization buildup if the polarization transfer flows through the central nuclei. According to the sample characterization data, a typical PSi NP of 150 nm size, 55% porosity (0.55 cm^3^ g^−1^ pore volume) and 100 m^2^ g^−1^ surface area contains on average 2.3 × 10^6 29^Si nuclei and 1.3 × 10^4^*P*_b_ centers (Fig. 1b–g). Therefore, a straightforward but incorrect calculation yields the number of ^29^Si to be polarized by one *P*_b_ center equal to ∼180—a common value for partially deuterated water glycerol mixtures (DNP juice).^82^ However, the number of ^29^Si nuclei that are central to the *P*_b_ electrons is only 4.7% of the total number of nuclei. This leads to around 3800 nuclei to be polarized per DNP-active *P*_b_ center, a much lower value than in typical DNP samples.

### 4.3 Rate limiting step for the polarization decay

The polarization decay in Si particles with endogenous electronic centers has been commonly considered to be limited by nSD from bulk ^29^Si to the ^29^Si in the core–shell interface.^8,37,67^ The arguments of low electron polarization at room temperature and orders of magnitude lower *T*_1e_ and *T*_2e_ than at DNP conditions further supported the hypothesis of nSD limiting relaxation. Although these arguments seem to be a reasonable for μm-sized Si particles, they are hardly applicable to our case of PSi NPs or to other types of Si NPs^26,30,38,83^ with crystalline cores on the order of 20 nm. If nSD is the rate limiting step for the relaxation in our samples, the polarization decay time *τ*_dec_ at room temperature should be tens of seconds at the slowest, according to the estimated *D*_diff_ ≈ 0.5–1.7 nm^2^ s^−1^. Unless this estimation is orders of magnitude incorrect, which is unlikely, nSD fails to explain the room temperature relaxation times in nanoscale Si.

**Fig. 7:**
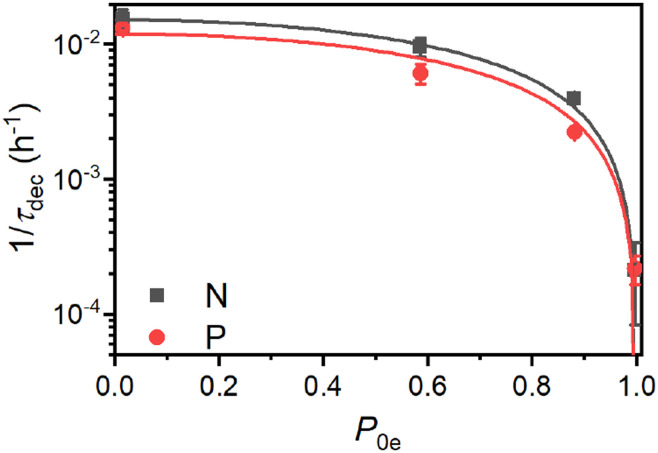
The dependence of the hyperpolarization decay rate *τ*_dec_^−1^ on the thermal electron polarization *P*_0e_ for the P and N PSi NPs. The shown experimental data was measured at (ordered with increasing *P*_0e_) 300 K, 7 T; 3.4 K, 3.4 T; 3.4 K, 7 T and 1.4 K, 6.7 T. For the fits, we assumed *τ*_dec_^−1^ ∝ 1 − *P*^2^_0e_ (see main text for discussion).

Fig. 7 depicts the dependence of nuclear hyperpolarization decay rates *τ*_dec_^−1^ on the thermal electron polarization *P*_0e_ at the given experimental conditions. The *τ*_dec_^−1^ follows a 1 − *P*^2^_0e_ scaling indicating that the nuclear relaxation appears to be governed by paramagnetic effects.^12,84^

We note that naturally abundant 160 nm (ref. 38) and 50 nm (ref. 9 and 26) non-porous Si NPs as well as the porous particles in this work all give similar room temperature relaxation times of around 50 min. Such a similar relaxation time across particles and the dependence on the ^29^Si abundance might indicate that even at room temperature the nuclear relaxation is governed by the same process as at the DNP conditions, *i.e.*, by the electron modified nSD across the spin diffusion barrier with fast relaxation of hypershifted ^29^Si spins. The observed 1 − *P*^2^_0e_ scaling (Fig. 7) would in this case not describe the paramagnetic relaxation itself but the actual spin transport *via* electron-nuclear four-spin flip-flops.^79^ Electron-nuclear four-spin flip-flops have a similar mechanism as triple spin flips causing indirect paramagnetic relaxation^12^ because both mechanisms involve an electron dipolar flip-flop, which provides energy for a nuclear excitation *e.g.*, a nuclear spin flip for triple spin flips and a nuclear flip-flop for electron-nuclear four-spin flip-flops. Therefore, both triple spin and four-spin flip-flops can be considered to have the same scaling with electron polarization, which for paramagnetic relaxation follows 1 − *P*^2^_0e_ dependence.^12^

Alternatively, the averaging of the HF couplings in combination with slow paramagnetic relaxation could explain the long room temperature relaxation times. Both interpretations would be in good agreement with isotope enrichment experiments^38^ which found a 3-fold decrease in *τ*_dec_ (from 48 to 17 minutes) upon increasing the ^29^Si abundance from 4.7% to 15%. The higher isotope abundance results in larger nuclear dipolar couplings and reduced frequency differences for nuclei close to the electron which would increase the transport of polarization across the spin diffusion barrier. Additionally, more *P*_b_ centers with a central ^29^Si results in a larger fraction of *P*_b_ centers with large (averaged) HF couplings which increases nuclear relaxation.

A major open question concerning the proposed rate limiting step of the room temperature decay is the lack of knowledge of the electron relaxation times with respect to the nuclear Larmor frequency *ω*_0n_. If the relaxation rate is much smaller than *ω*_0n_, the HF couplings are not averaged and the situation is similar to low temperature DNP conditions. For much faster relaxation rates than *ω*_0n_, the thermal electron polarization of 1.6% at room temperature and 7 T leads to (pseudo-)contact shifts due to the partially averaged HF couplings.^85–88^ Even in this averaged case the (pseudo-)contact shift is expected to be large compared to the nuclear dipolar couplings mediating the nSD.

### 4.4 Samples and experimental conditions

Above we discussed that DNP likely originates from triple spin flips which require strong HF coupling of the involved electrons to the central ^29^Si nuclei of *P*_b_ centers for a fast polarization transfer between them. This is followed by a slow transfer from this central, strongly hypershifted nucleus to the bulk nuclei, followed by fast spin diffusion in the bulk. For the decay, the inverse process happens with paramagnetic relaxation instead of triple spin flip DNP. Similar to the buildup, the decay time appears to be governed by the hyperpolarization transfer from the bulk to the strongly hypershifted ^29^Si nuclei. Since both the buildup and the decay involve a single rate limiting step, a mono-exponential hyperpolarization dynamics^50^ is expected and found experimentally (Fig. 2, section S3.3, ESI†).

For a mono-exponential hyperpolarization dynamics, the buildup time, steady-state polarization and thermal electron polarization can be used to define hyperpolarization injection and decay rate constants during buildup,^50^ which we briefly summarize below and in more detail in section S3.4, ESI.† With these rate constants, it is possible to quantify the nuclear relaxation during buildup and compare it to the rate with which hyperpolarization is created.

In the rate-equation model, the buildup time *τ*_bup_ depends on two competing processes: the nuclear polarization injection with rate constant *k*_W_ and the nuclear relaxation with rate constant *k*^bup^_R_. Together with the thermal electron polarization *P*_0e_, we can describe the buildup as:^50^1a
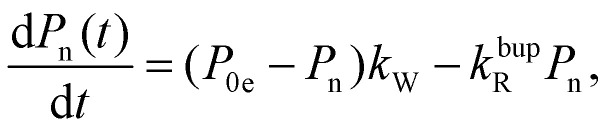
1b
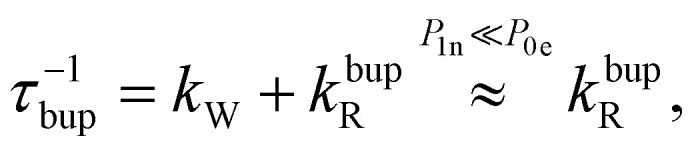
1c
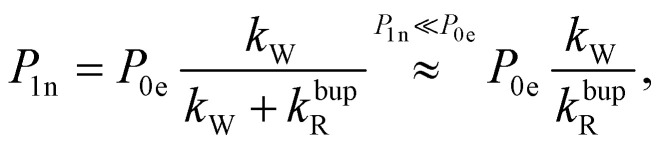
where *P*_1n_ is the steady-state nuclear polarization reached by the end of the DNP process. For the decay, the decay rate constant *k*^dec^_R_ = *τ*_dec_^−1^ provides sufficient description since MW radiation is off.

**Fig. 8:**
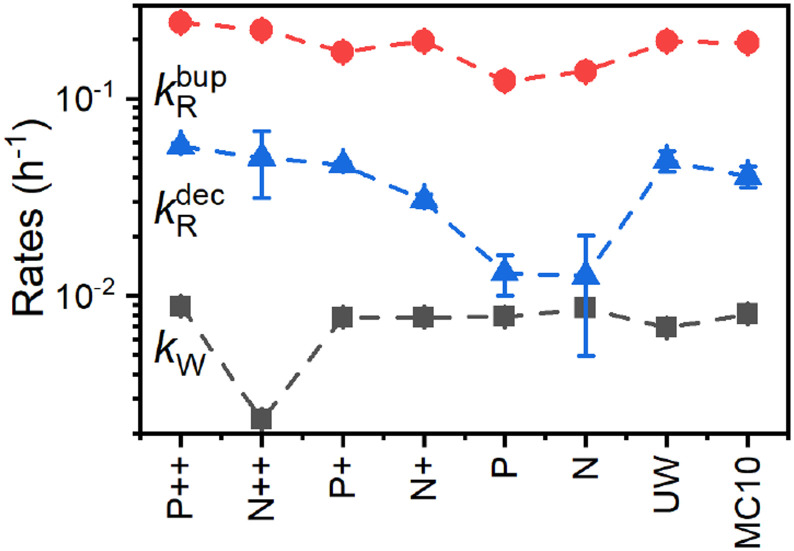
Polarization buildup (dark squares and red circles) and decay (blue triangles) rates (eqn (1a)–(1c)) for the PSi NPs with different doping and oxidation. The data was acquired at 6.7 T, 1.4 K. Lines are a guide for the eye.

Fig. 8 compares the DNP injection and relaxation rates during buildup and decay at 6.7 T, 1.4 K. The rates at other conditions and a more detailed discussion of these are given in section S3.4, ESI† with the main results summarized below. The relaxation rates during the buildup are an order of magnitude larger than the injection rates and, hence, govern the buildup time. The imbalance between buildup and relaxation rates results in moderate steady-state polarization (and enhancements) compared to ^1^H or ^13^C enhancements under similar conditions (ref. 89 and references therein for state-of-the-art enhancements). DNP injection appears rather uniform across the samples while the relaxation rates show a variation between samples. Thus, differences between samples mostly originate from different relaxation properties. Furthermore, the DNP injection shows a less pronounced dependence on the experimental conditions than the relaxation rates (Fig. S26, ESI† and discussion thereof). However, suppressing the relaxation with lower temperatures (1.4 K instead of 3.4 K) shows at best only a modest improvement because relaxation enhancement by MW irradiation^89^ becomes more pronounced as evident by the much higher relaxation rates during buildup (*k*^bup^_R_) compared to decay (*k*^dec^_R_) as shown in Fig. 8. Reduction of this relaxation enhancement *e.g.*, by higher fields, lower temperatures or shortening of electronic relaxation times,^89^ offers the possibility of higher enhancements and polarization levels.

By performing the correlation analysis, we further connect the structural properties of PSi NPs, *P*_b_ centers and hyperpolarization. The nuclear hyperpolarization at 6.7 T, 1.4 K (Fig. 2) is nearly independent from the number of *P*_b_ centers (Fig. 1g) expressed by a correlation coefficient of 0.04 across all samples but correlates (0.44) with the interface density of the *P*_b_ centers (number of *P*_b_ defects divided by the specific surface area, Fig. 1d and g). The density of *P*_b_ centers correlates strongly negatively across all the samples with the specific surface area (coefficient equals to −0.9) because the increase of surface area does not lead to the proportional increase of the number of *P*_b_ centers (Fig. 1d and g). Such an independence of the number of *P*_b_ centers suggests existence of a limit for their formation at least for the oxidation methods applied here. A more detailed discussion of the correlation analysis can be found in section S3.4, ESI.†

Before concluding, we want to highlight specific aspects of the investigated PSi NPs. First, the ability to create nuclear hyperpolarization in PSi NP appears exceptionally robust due to the core–shell nature of the particles with the paramagnetic centers protected from the environment. Specifically, the *P*_b_ centers form at the interface between the crystalline pore wall cores and the oxide shell. Hence, the *P*_b_ centers as well as the nuclear hyperpolarization are largely shielded from everything outside each particle *e.g.*, different particle coating or solution media.^83^ This is exemplified by the inertness to the presence of the catalytic Au NPs in PSi NPs: removing the Au NPs used as an etching catalyst shows no clear effect on the hyperpolarization process and the observed changes are in line with other additional oxidation steps (Fig. 4). This suggests that the pores in PSi NPs with their large surface area available for coating could be used for loading with additional substances to add further diagnostic or therapy capabilities.

Second, the nuclear hyperpolarization in the bulk seems to be inert with respect to a wide range of bulk defects and their densities: both boron and phosphorous doping with densities up to ∼10^16^ cm^−3^ show similar high polarization levels (Fig. 2 and 3). Furthermore, the light doping of ∼5 × 10^14^ cm^−3^ is superior compared to the most pure UW sample. The increase of doping level to the order of 10^18^ cm^−3^ becomes detrimental for achievable hyperpolarization levels due to the onset of the wavefunction overlap of the dopants. At room temperature, high densities of thermally excited mobile charge carriers from shallow dopants strongly increase the nuclear relaxation.^67^ The least pure MC10 sample possesses various different dopants, with energy levels often deep in the bandgap of Si and thus with narrow defect wave functions, which makes DNP performance of MC10 sample very similar to the best electronic wafer grade samples. Such a stable polarization process is important if other, especially bottom-up manufacturing techniques should be employed as these offer a reduced control over the bulk purity compared to the top-down approach of the current work.

## 5 Conclusion

We employed low-load metal assisted catalytic etching (LL-MACE)^31,32^ to fabricate a variety of porous Si NPs from electronic grade single crystal Si wafers. This top-down fabrication approach allowed us to vary dopant type and density while achieving nearly identical surface properties and crystallinity in all the NPs. A separate oxidation step led to the formation of electronic *P*_b_ centers with similar structure and surface density for all the types of PSi NPs. This resulted in the successful and similar DNP injection in all samples with the polarization differences mostly ascribed to relaxation. The robustness of the hyperpolarization process to different shallow dopant concentrations and metallurgical grade Si samples containing deep dopants enables and justifies the use of a wide range of manufacturing methods with eventually poor control over the bulk composition *e.g.*, bottom-up synthesis methods. The highest steady-state polarization levels were achieved with lightly (∼10^14^ cm^−3^) phosphorous or boron-doped samples. Measurements at 7 T (3.4 K) and 3.35 T (1.4 and 3.4 K) gave lower polarization levels than the 6% achieved at 6.7 T, 1.4 K. Room temperature decay times of the studied samples exceeded one hour—the longest hyperpolarization decay time obtained so far in Si NPs to our knowledge although slightly longer saturation-recovery *T*_1_ times (102 ± 10) min have been reported for 21 nm (comparable to pore walls in the present work) particles.^90^

The gained insights about *P*_b_ centers enabled us to shed light on the polarization transfer from the electron spins to ^29^Si. Owing to the core–shell nature of the PSi NPs with the *P*_b_ centers at the interface between the pore wall cores and oxide shell, nuclear spin diffusion is required to transport the hyperpolarization across the sample. The central ^29^Si nuclei of the *P*_b_ centers with hyperfine couplings around 300 MHz are predominantly hyperpolarized by DNP. The large difference in frequency compared to the bulk ^29^Si spins seems to cause a slow transport of polarization from the central *P*_b_^29^Si nuclei towards the bulk, which causes the long buildup and relaxation times in the presence of fast bulk spin diffusion. Isotope labelling may improve the transport across the spin diffusion barrier and, therefore, improve the NMR signal through the increased enhancement and number of magnetically active spins. The disadvantages of isotope labelling are high sample cost and shortened room temperature decay times. The described hyperpolarization process could be translated to create other slowly relaxing NPs with sizes down to possibly 10 nm. For this, three properties of (Si) NPs appear essential: (i) a low bulk relaxation, (ii) DNP on the outer surfaces of the particles and (iii) a slow transport from the bulk to the surface *e.g.*, a strongly localized wavefunction of a surface paramagnetic center with large hyperfine coupling compared to the nuclear line width.

## Author contributions

Conceptualization: G. v. W., M. E., J. R., V. P. L., S. K., M. I. K., K. T. Data curation: G. v. W., V. H., M. I. K., K. T. Methodology: G. v. W., A. H., M. M. A., M. E., M. I. K., K. T. Formal analysis: G. v. W., K. T. Investigation: G. v. W., A. H., V. H., J. J., J. O. M., S. K., M. I. K., K. T. Funding acquisition: M. E., J. R., V. P. L., S. K., M. I. K., K. T. Resources: J. O. M., M. E., S. K., V. P. L., M. I. K. Writing – original draft: K. T., G. v. W. Writing – review and editing: G. v. W, M. E., J. R., S. K., M. I. K., K. T. All authors approved the draft.

## Data availability

All the data and analysis scripts for this article are available at ETH Library at https://doi.org/10.3929/ethz-b-000679152.

## Conflicts of interest

There are no conflicts to declare.

## Supplementary Material

NR-016-D4NR02603A-s001

NR-016-D4NR02603A-s002
