# Supplementary figures and images for: Controlled synthesis and characterization of porous silicon nanoparticles for dynamic nuclear polarization
Source: Nanoscale. 2024 Sep 18;16(41):19385–99. doi: 10.1039/d4nr02603a (PMC11430043; doi:10.1039/d4nr02603a)

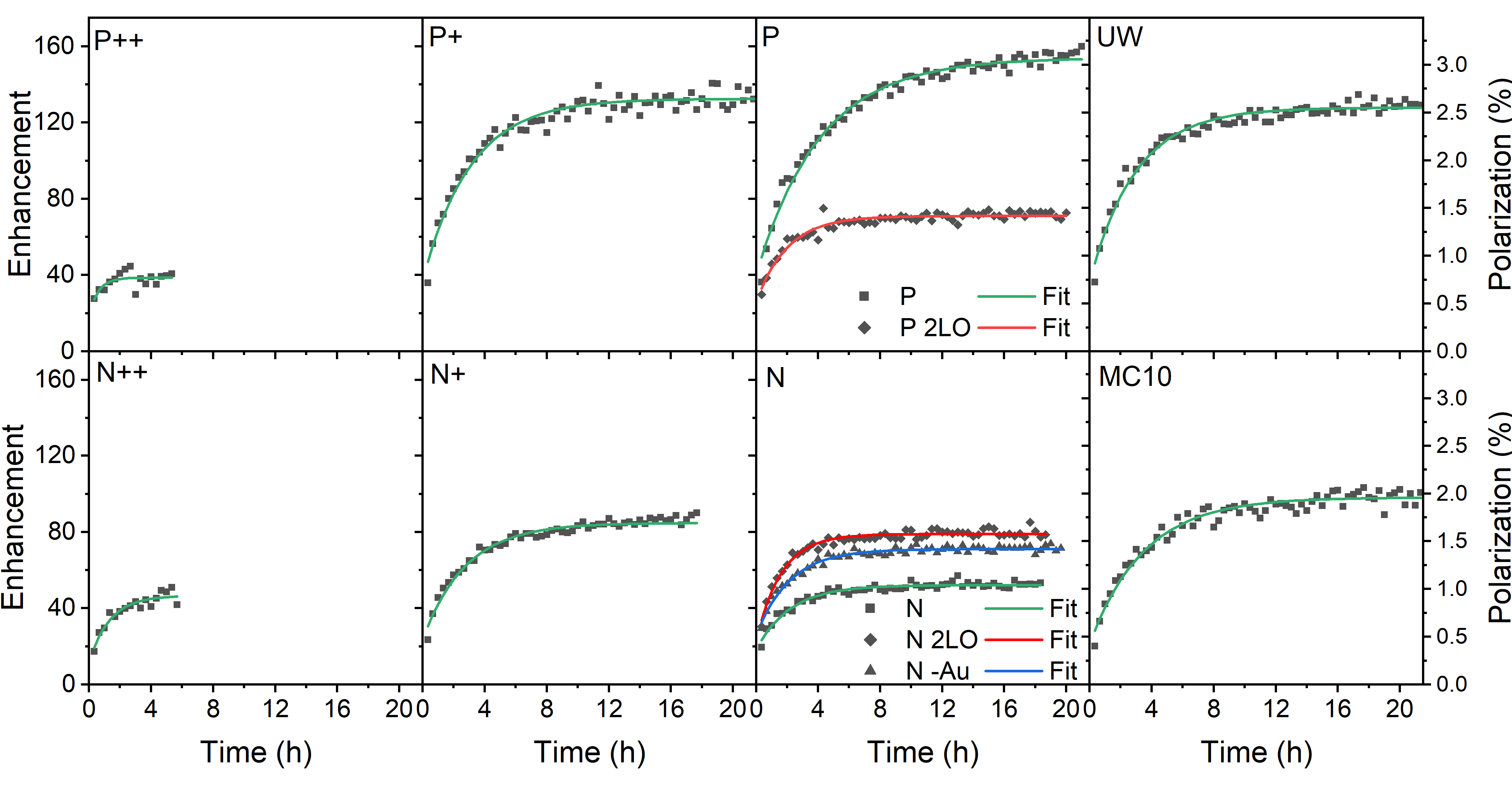

Supplement: NR-016-D4NR02603A-s001 [file NR-016-D4NR02603A-s001.zip › ESI Figs/Supp_Bup_Grid_3p4T.png]

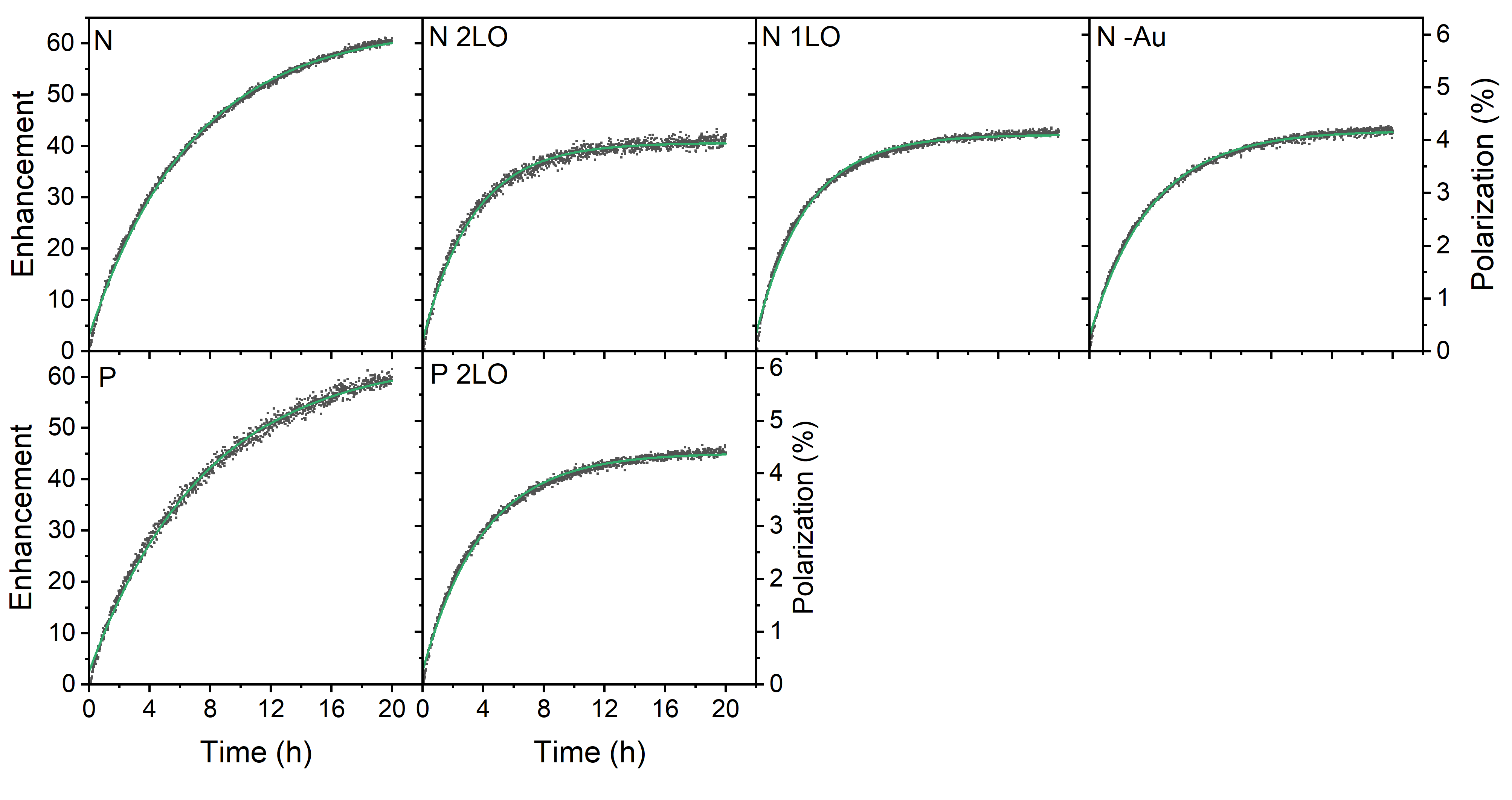

Supplement: NR-016-D4NR02603A-s001 [file NR-016-D4NR02603A-s001.zip › ESI Figs/Supp_Bup_Oxy_Grid_6p7.png]

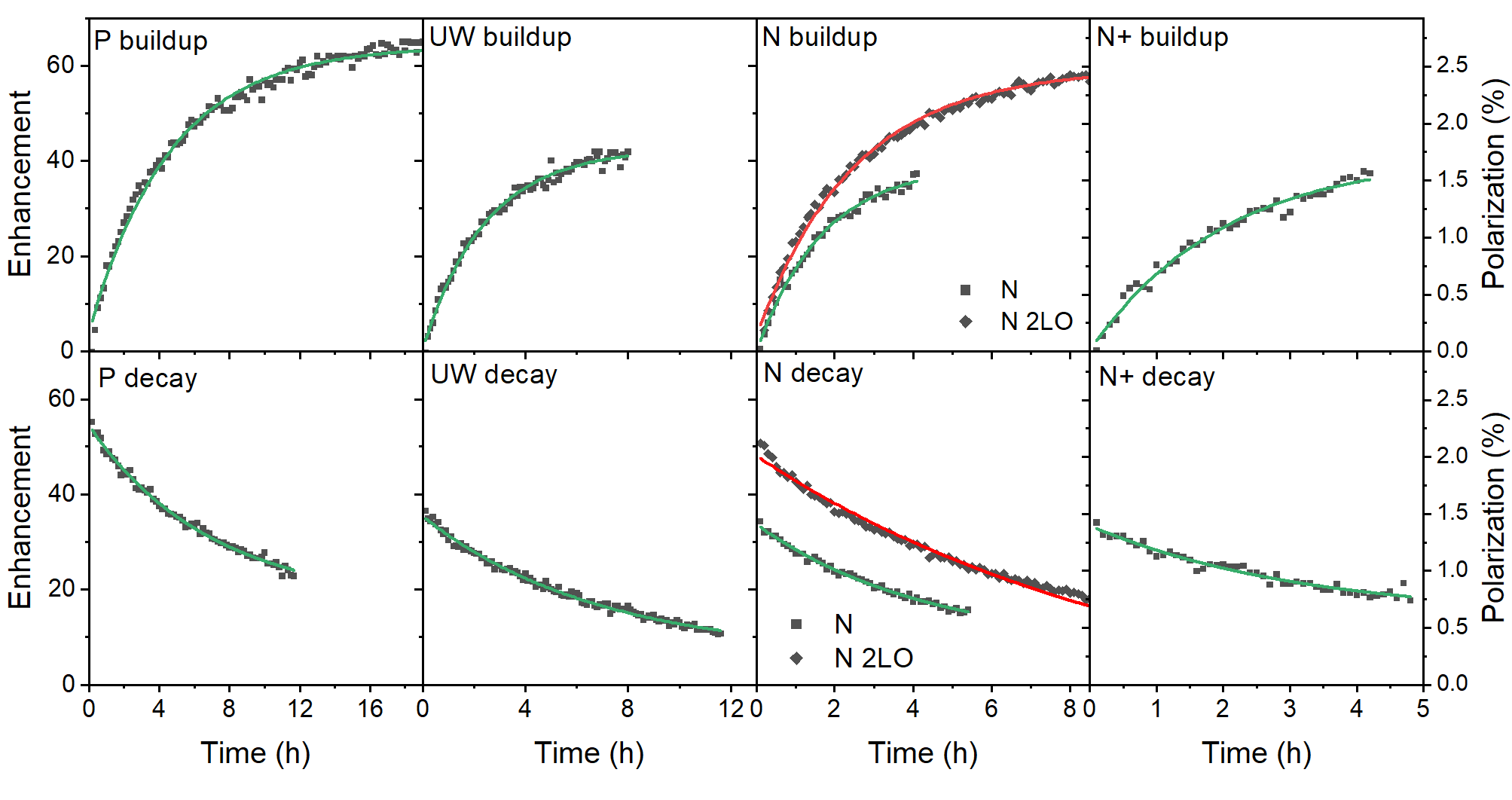

Supplement: NR-016-D4NR02603A-s001 [file NR-016-D4NR02603A-s001.zip › ESI Figs/Supp_BupDec_Grid7T.png]

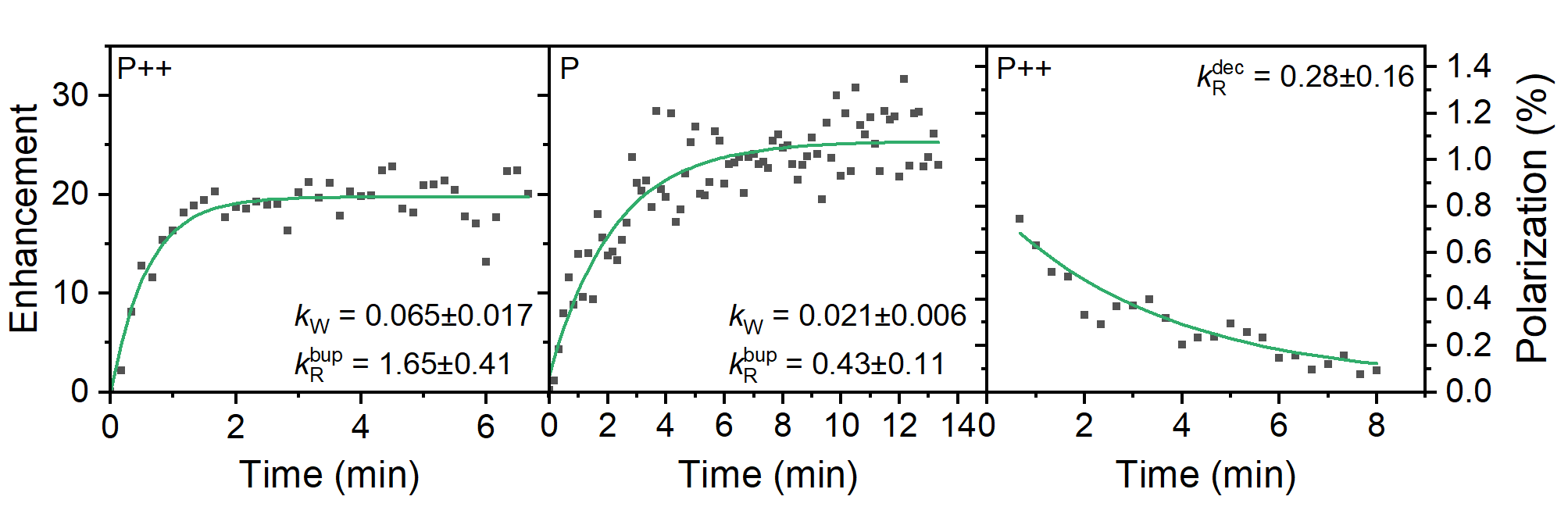

Supplement: NR-016-D4NR02603A-s001 [file NR-016-D4NR02603A-s001.zip › ESI Figs/Supp_BupDecay_3p35T.png]

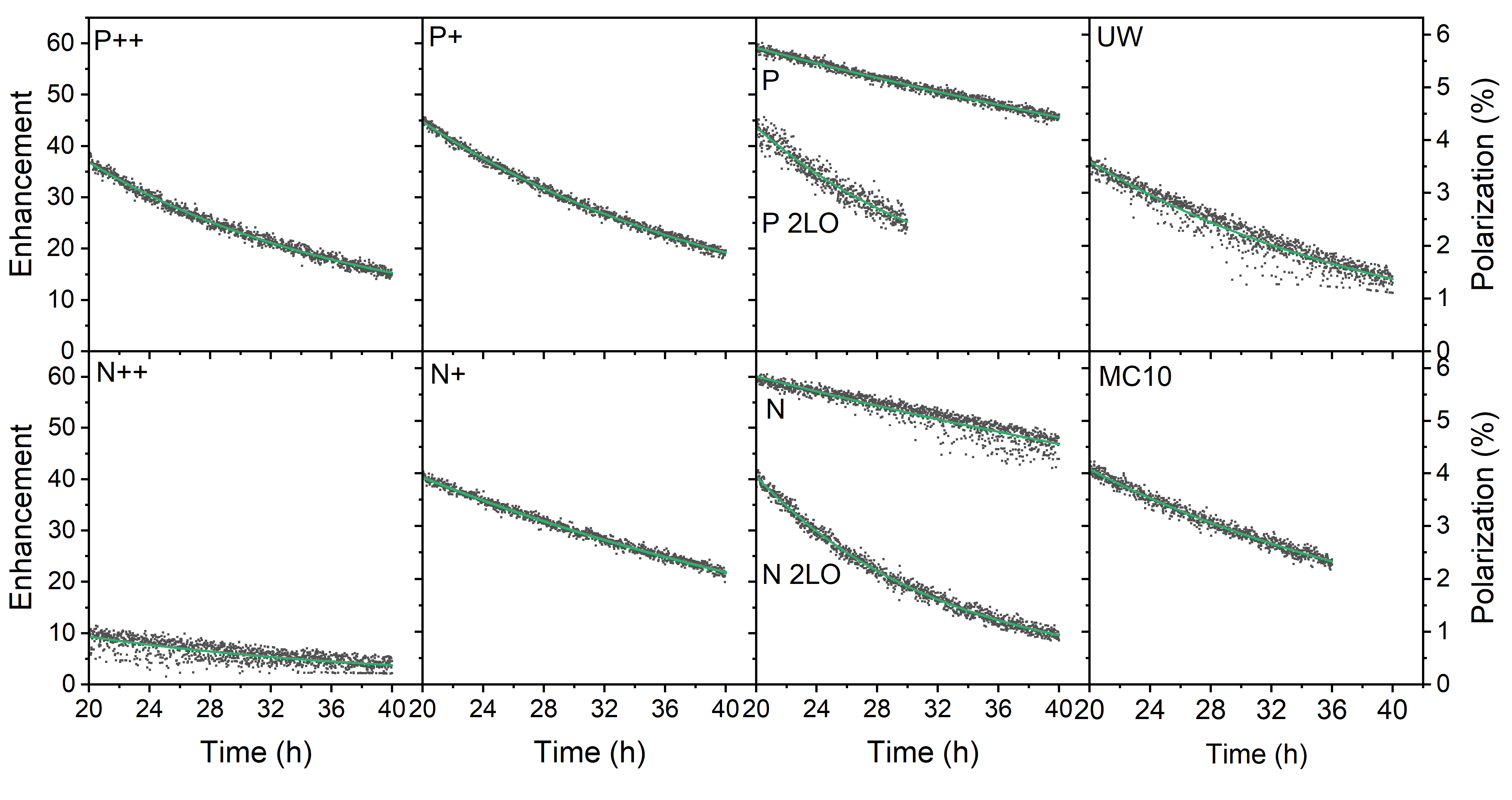

Supplement: NR-016-D4NR02603A-s001 [file NR-016-D4NR02603A-s001.zip › ESI Figs/Supp_Dec_Grid_Enh6p7T.png]

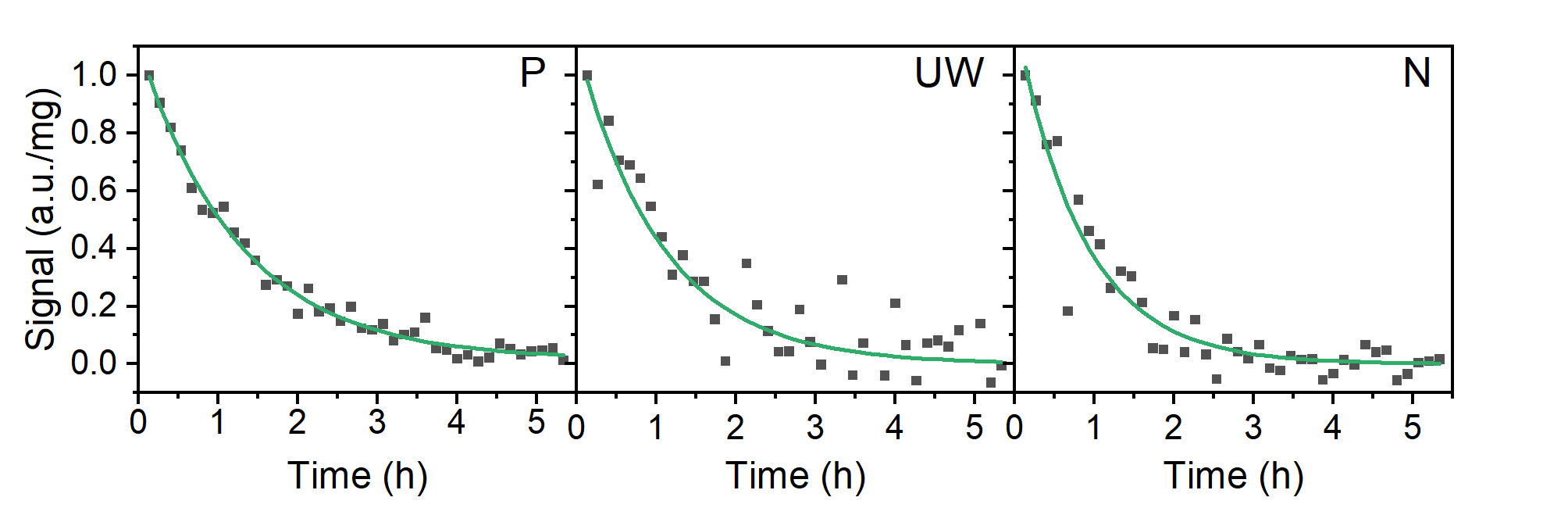

Supplement: NR-016-D4NR02603A-s001 [file NR-016-D4NR02603A-s001.zip › ESI Figs/Supp_Decay_RoomT.png]

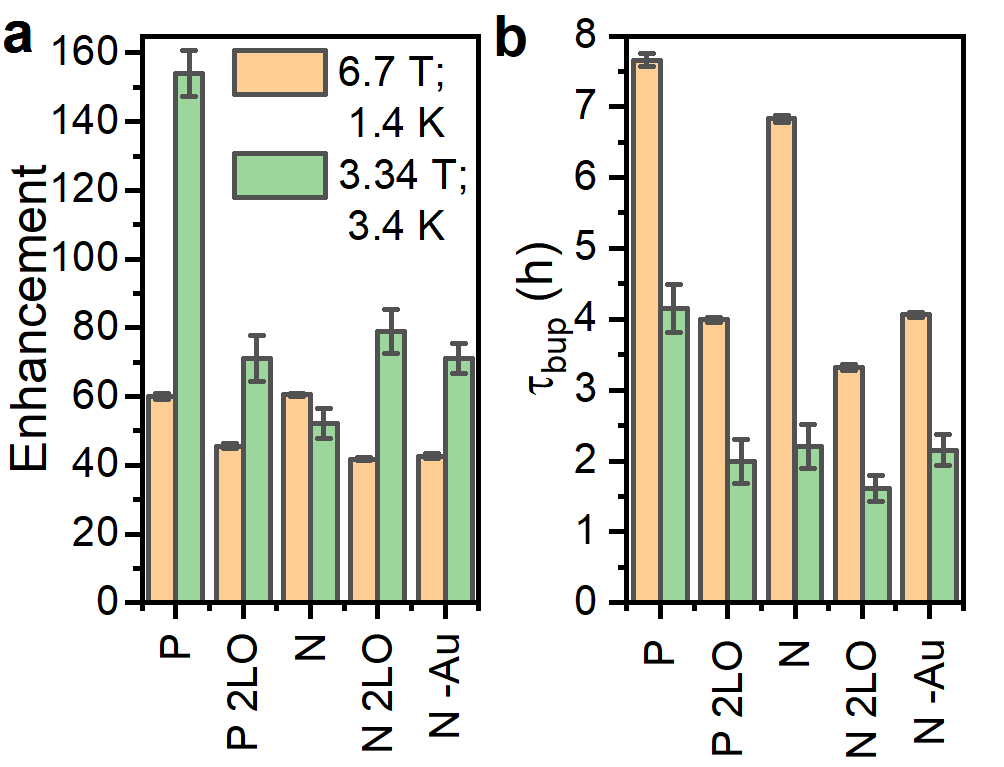

Supplement: NR-016-D4NR02603A-s001 [file NR-016-D4NR02603A-s001.zip › ESI Figs/Supp_Enh_Tbup_Oxy.png]

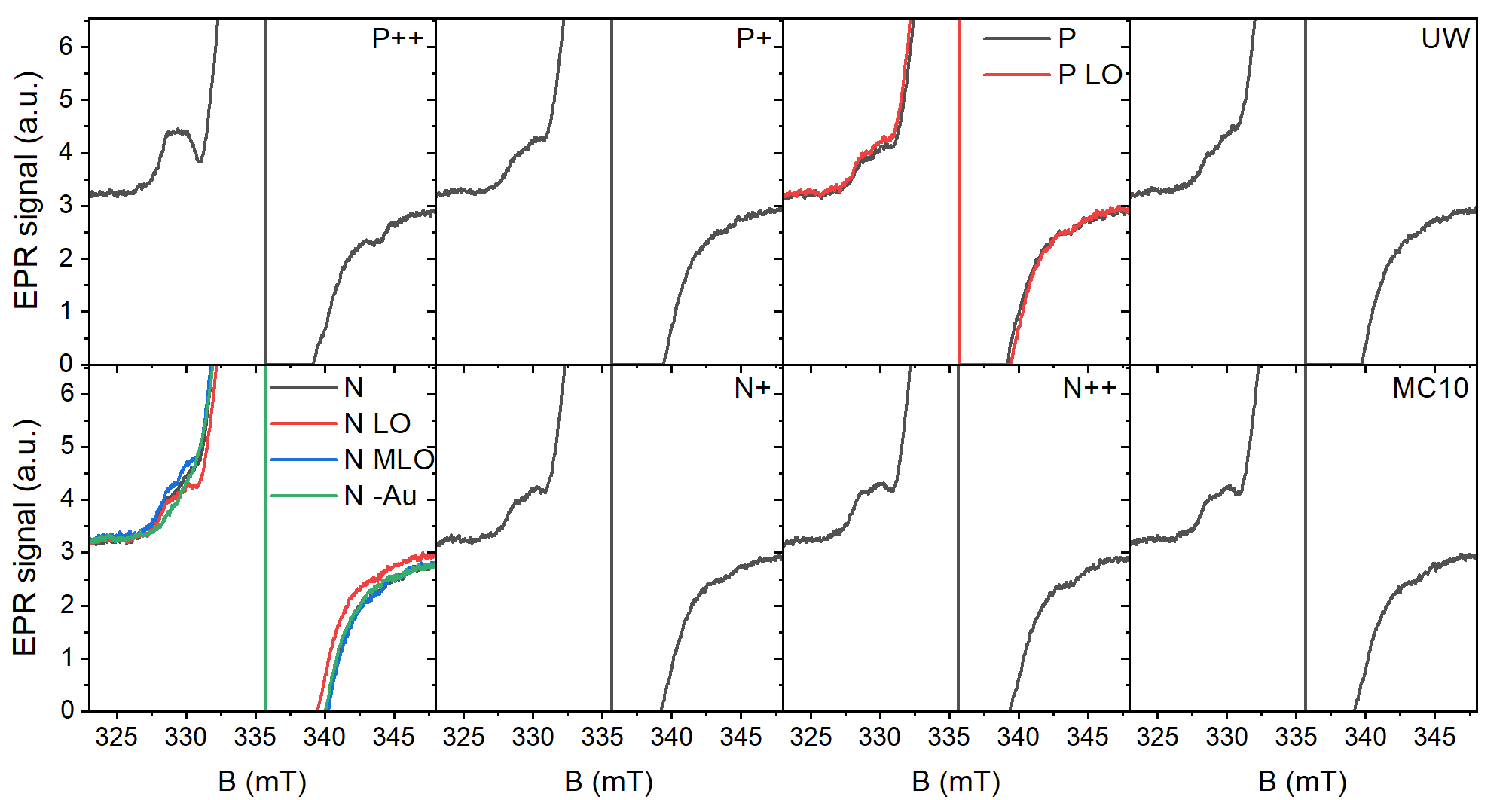

Supplement: NR-016-D4NR02603A-s001 [file NR-016-D4NR02603A-s001.zip › ESI Figs/Supp_EPR_HF_Grid.png]

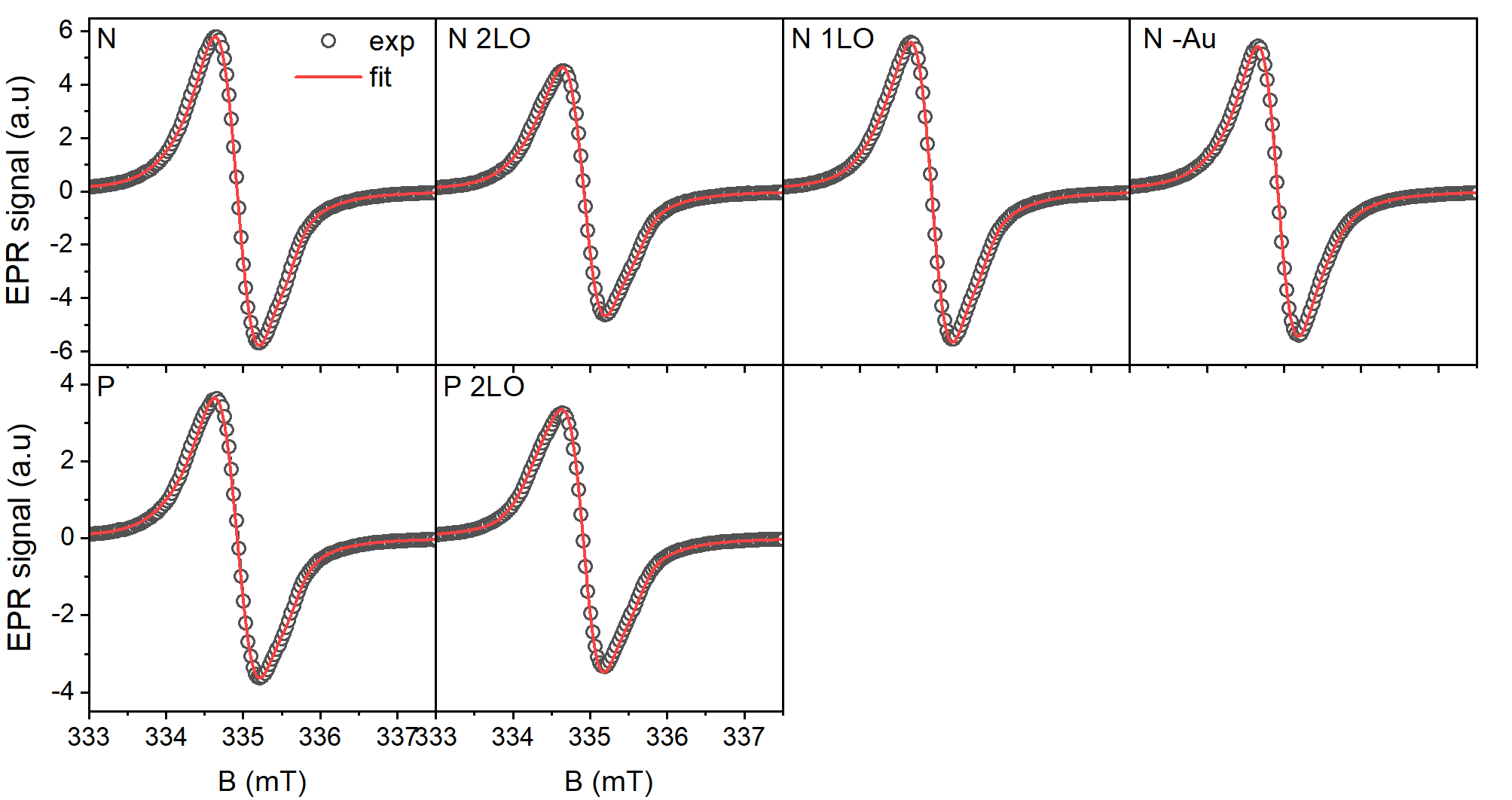

Supplement: NR-016-D4NR02603A-s001 [file NR-016-D4NR02603A-s001.zip › ESI Figs/Supp_EPR_Oxy_Grid.png]

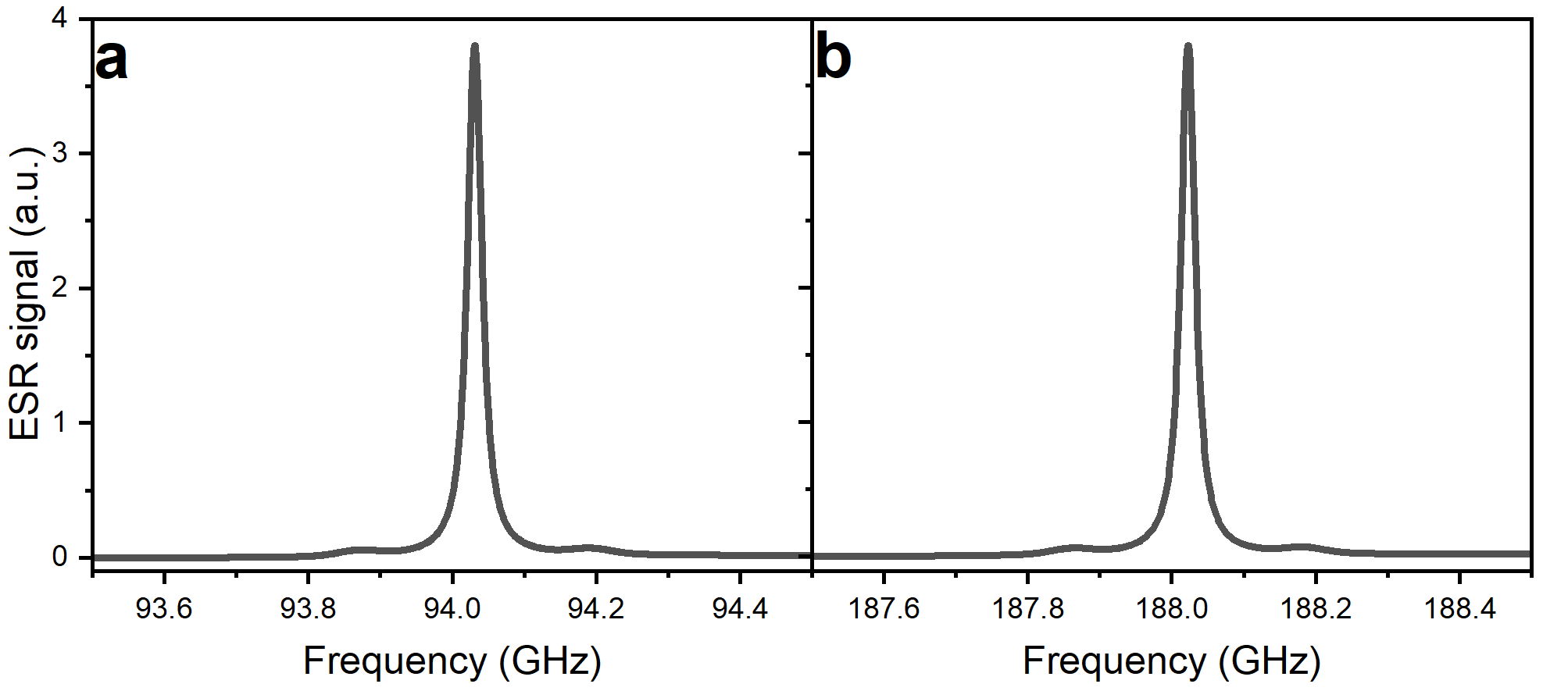

Supplement: NR-016-D4NR02603A-s001 [file NR-016-D4NR02603A-s001.zip › ESI Figs/Supp_EPR_simulation.png]

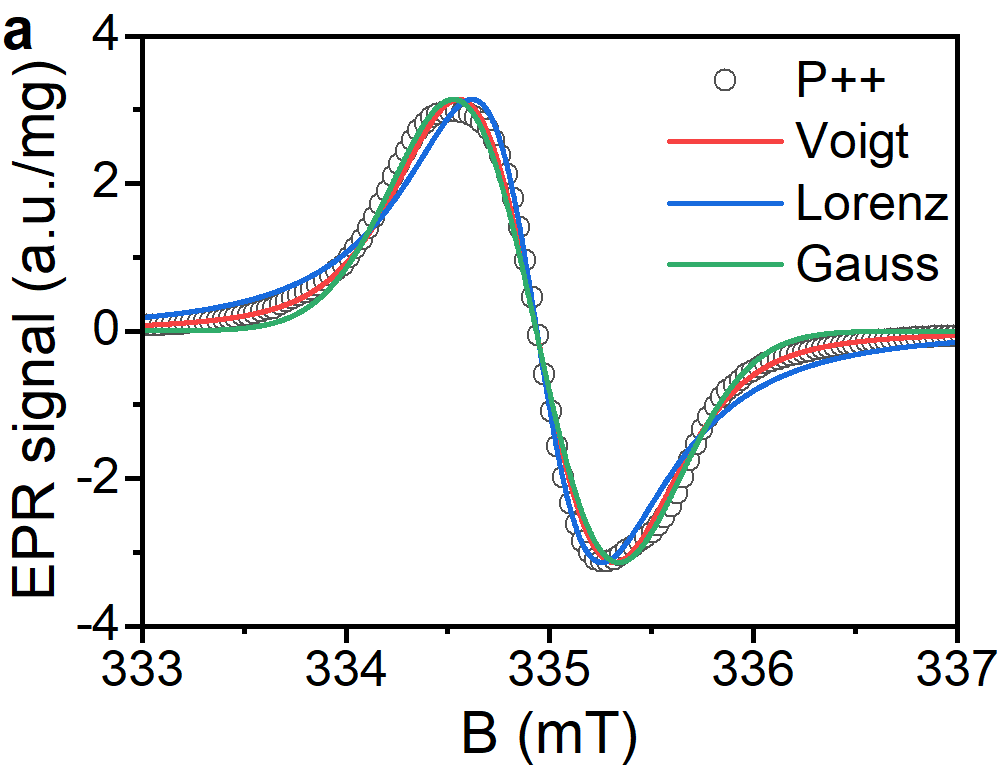

Supplement: NR-016-D4NR02603A-s001 [file NR-016-D4NR02603A-s001.zip › ESI Figs/Supp_EPR_spectrum_compare_fits.png]

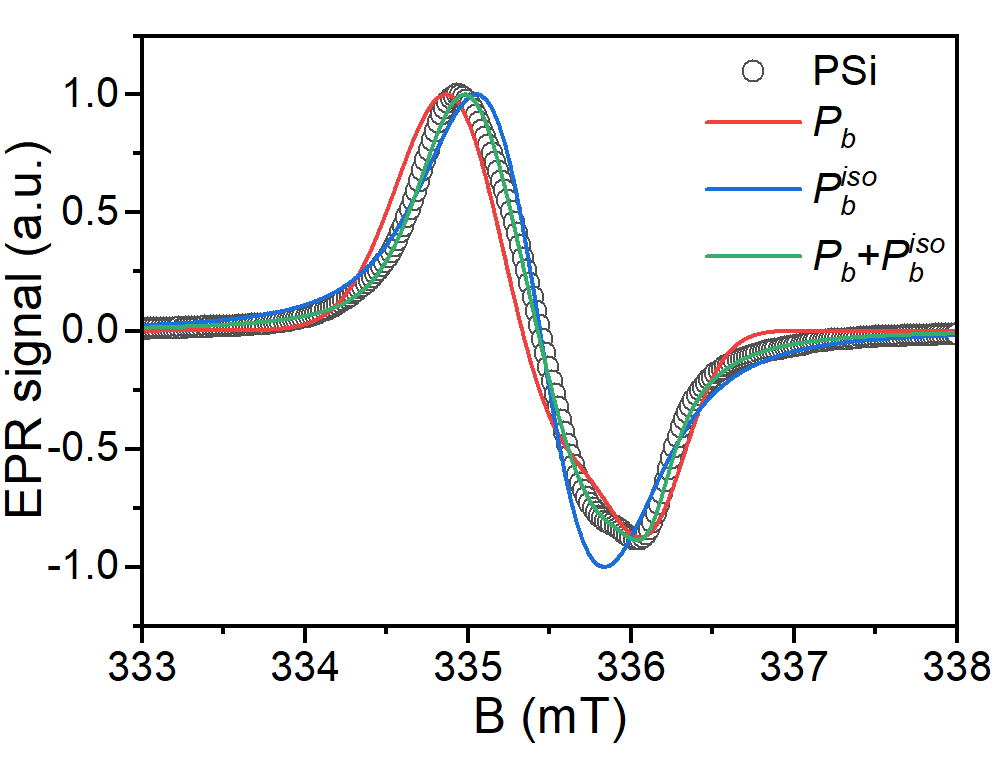

Supplement: NR-016-D4NR02603A-s001 [file NR-016-D4NR02603A-s001.zip › ESI Figs/Supp_EPR_TOPSi.png]

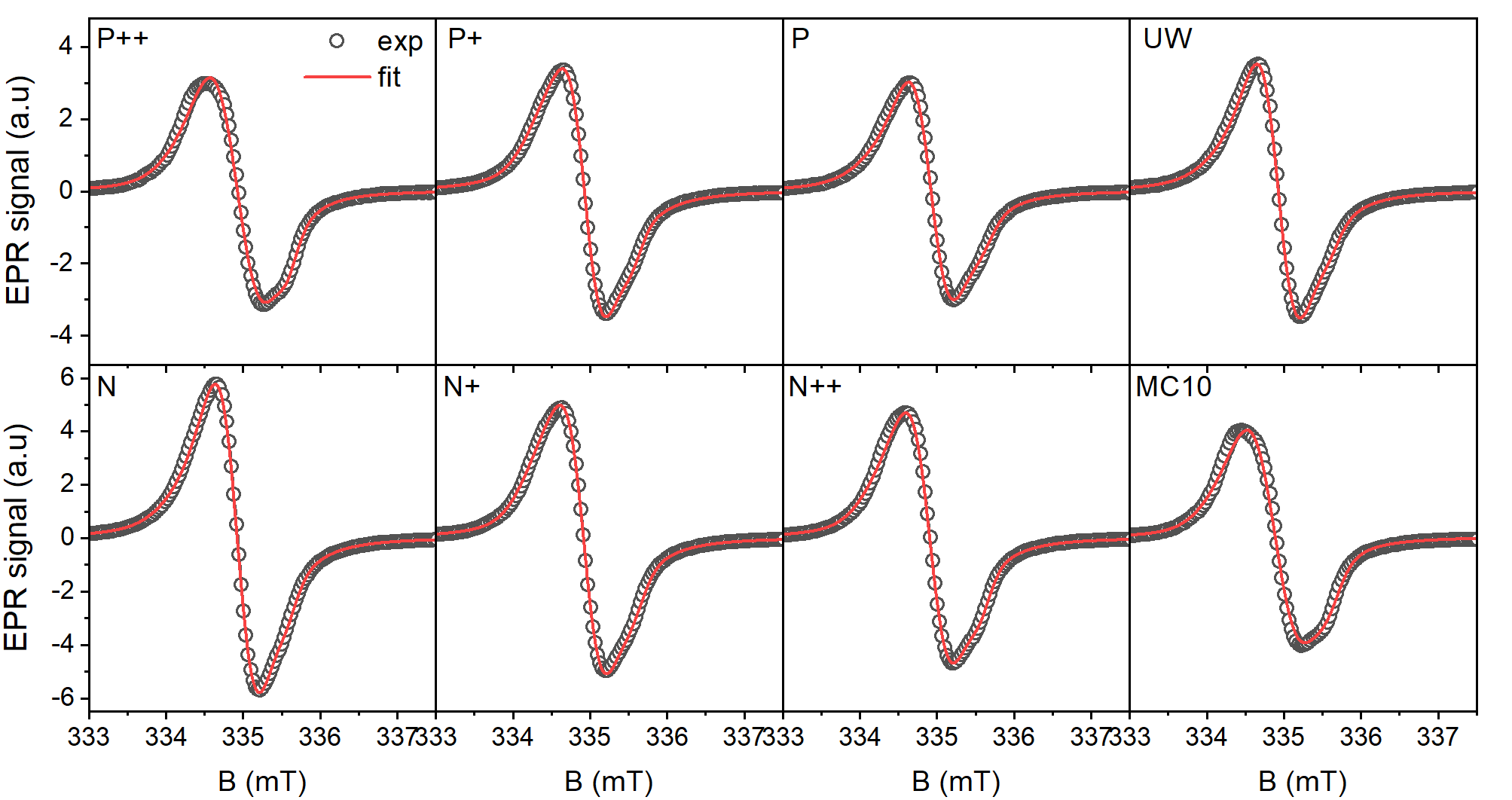

Supplement: NR-016-D4NR02603A-s001 [file NR-016-D4NR02603A-s001.zip › ESI Figs/Supp_EPR_Type_Grid.png]

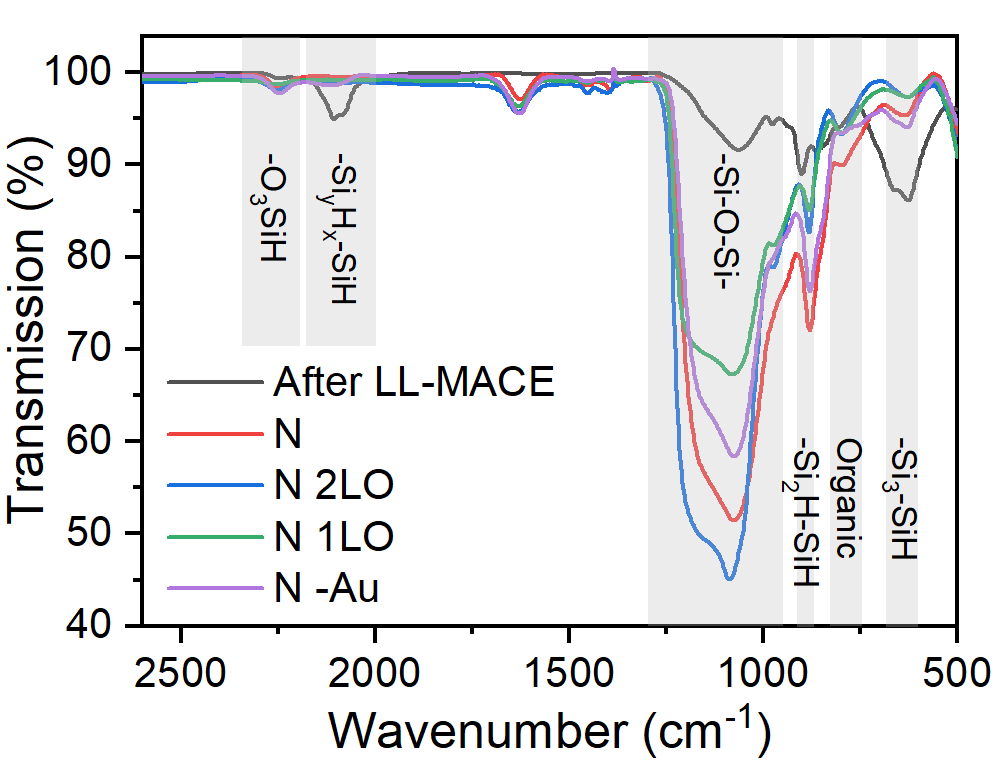

Supplement: NR-016-D4NR02603A-s001 [file NR-016-D4NR02603A-s001.zip › ESI Figs/Supp_FTIR_N_oxidation.png]

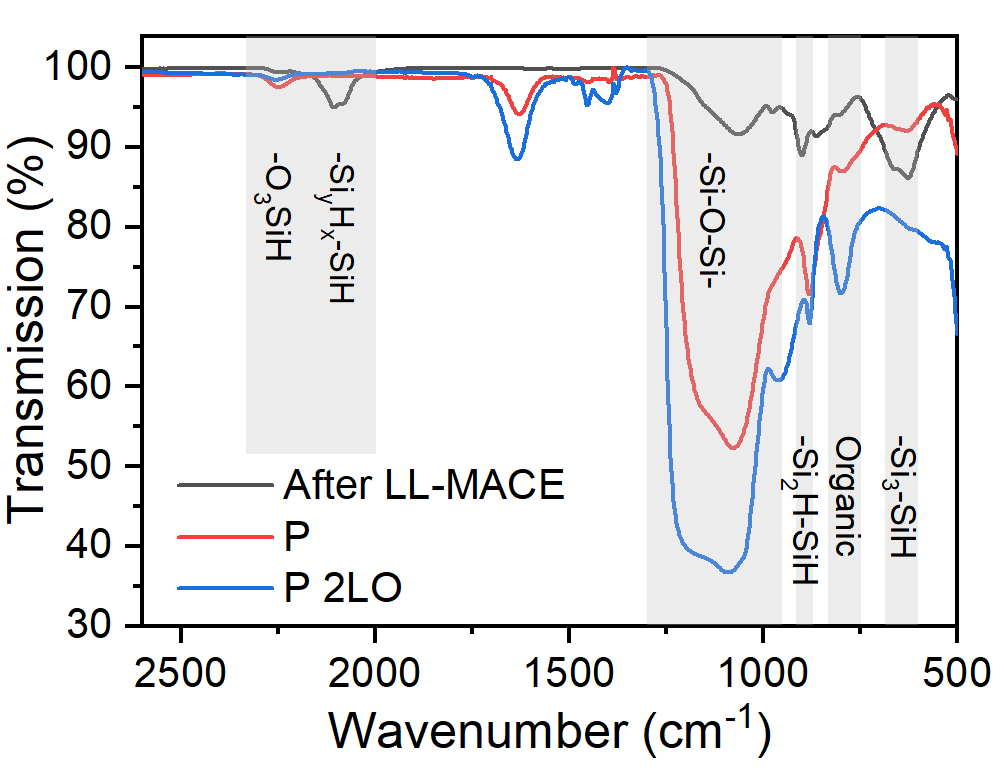

Supplement: NR-016-D4NR02603A-s001 [file NR-016-D4NR02603A-s001.zip › ESI Figs/Supp_FTIR_P_oxidation.png]

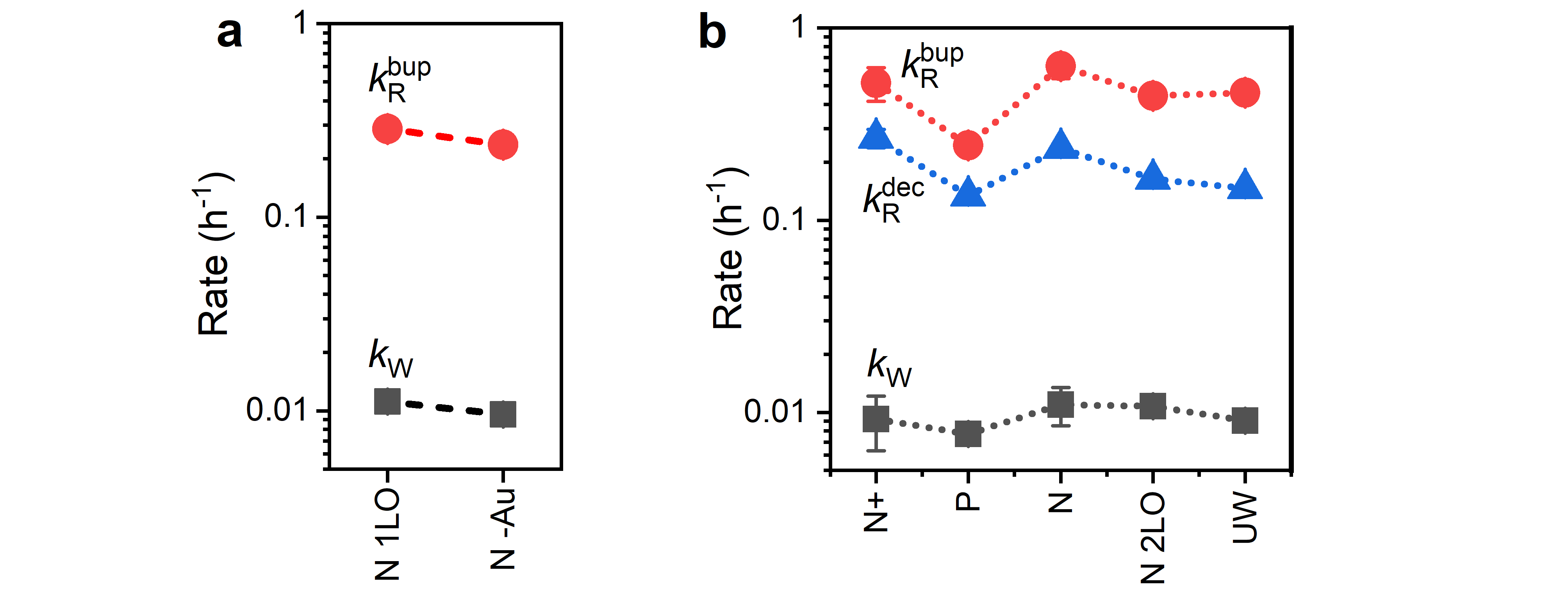

Supplement: NR-016-D4NR02603A-s001 [file NR-016-D4NR02603A-s001.zip › ESI Figs/Supp_LeftoverRates_6p7T_7T.png]

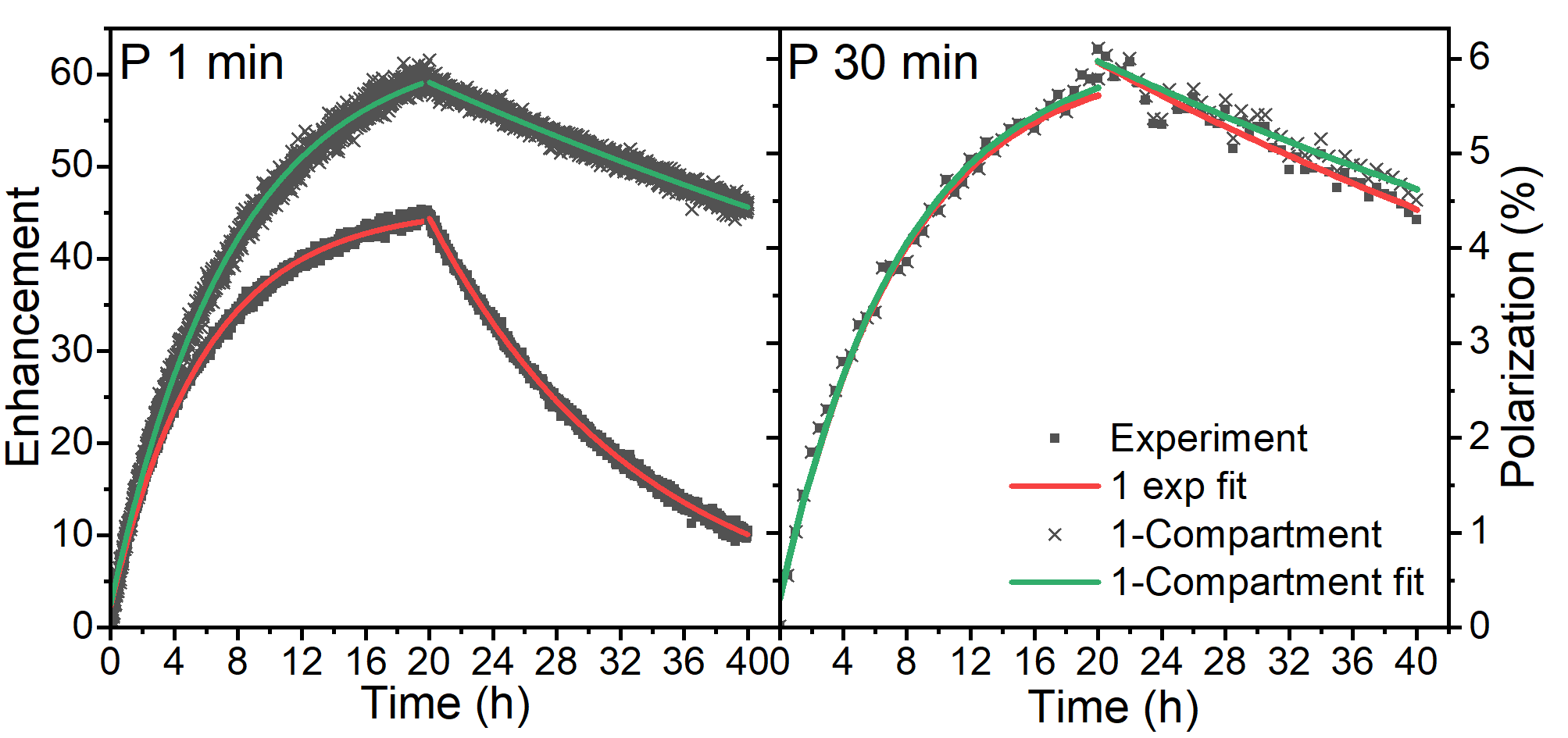

Supplement: NR-016-D4NR02603A-s001 [file NR-016-D4NR02603A-s001.zip › ESI Figs/Supp_One_Compartment_Evaluation.png]

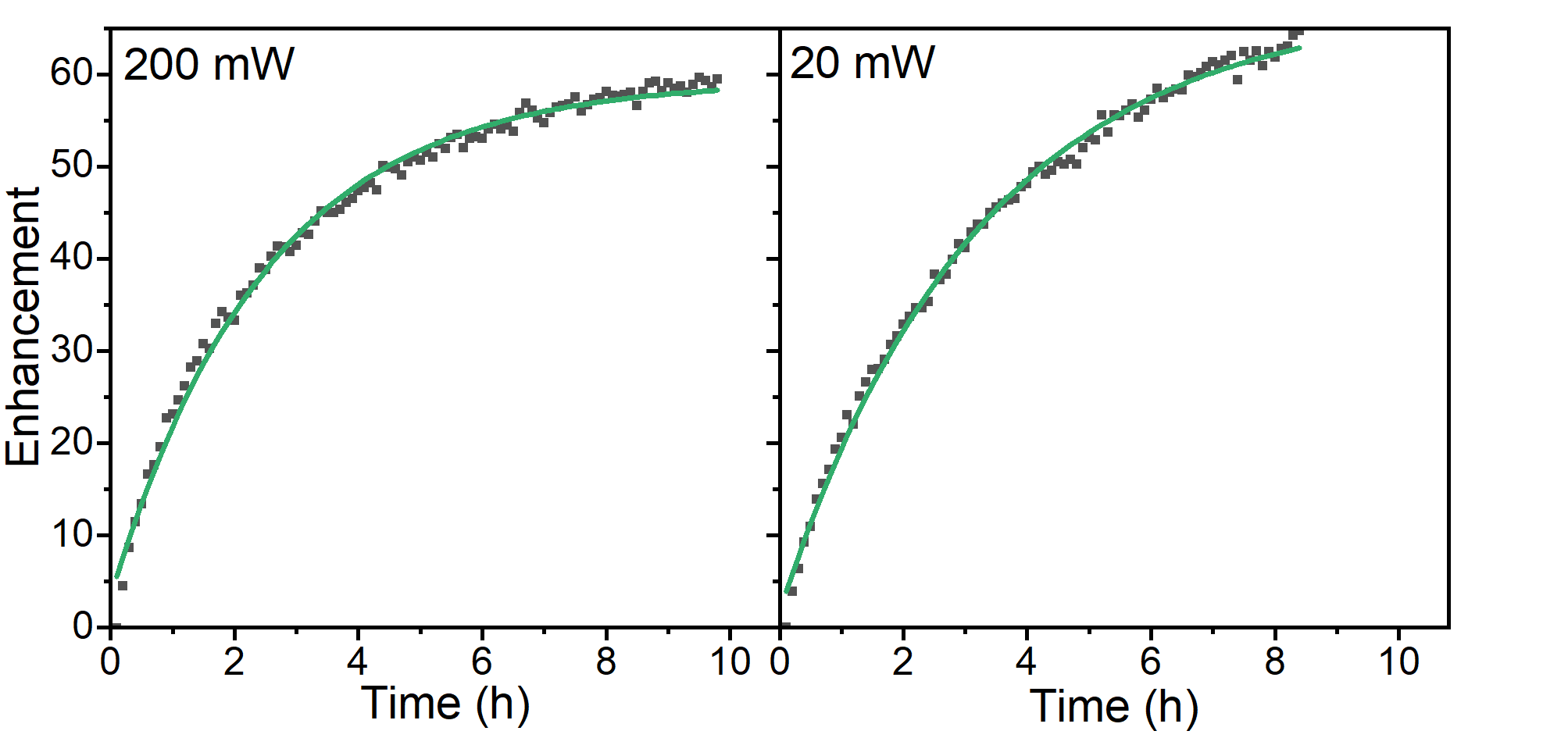

Supplement: NR-016-D4NR02603A-s001 [file NR-016-D4NR02603A-s001.zip › ESI Figs/Supp_Power_7T.png]

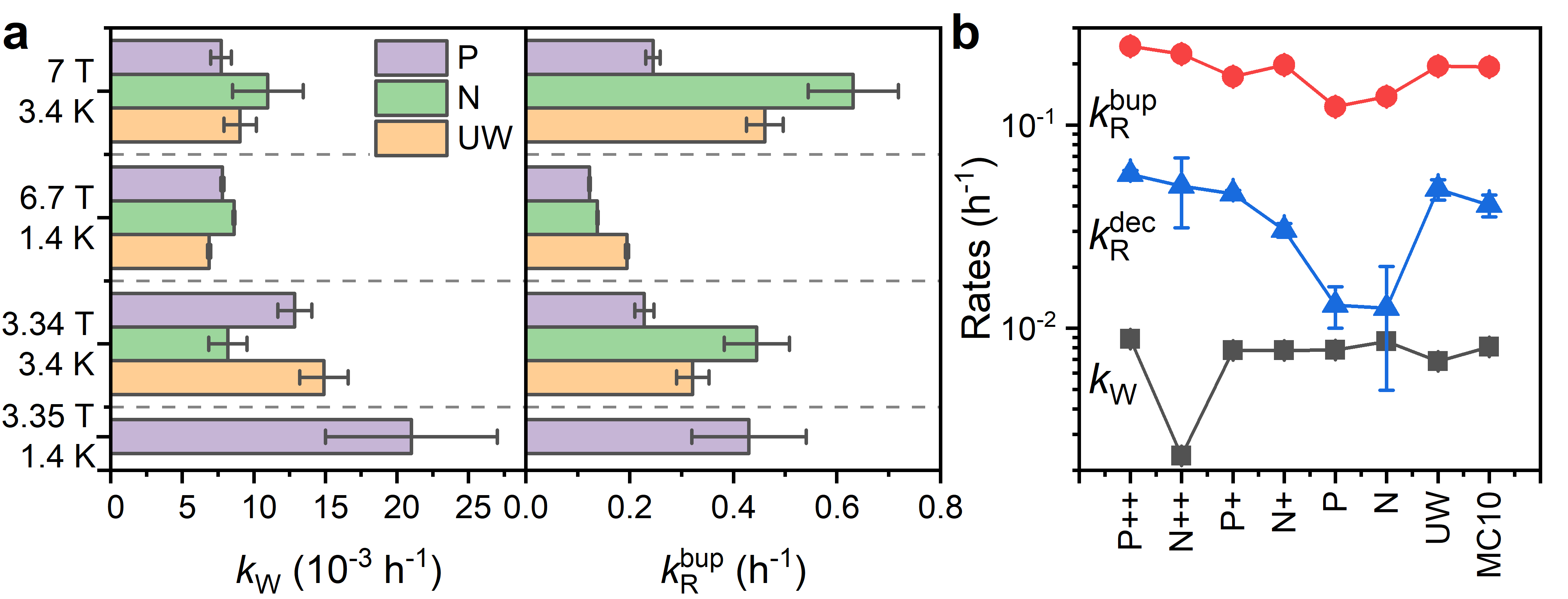

Supplement: NR-016-D4NR02603A-s001 [file NR-016-D4NR02603A-s001.zip › ESI Figs/Supp_Rates_6p7T.png]

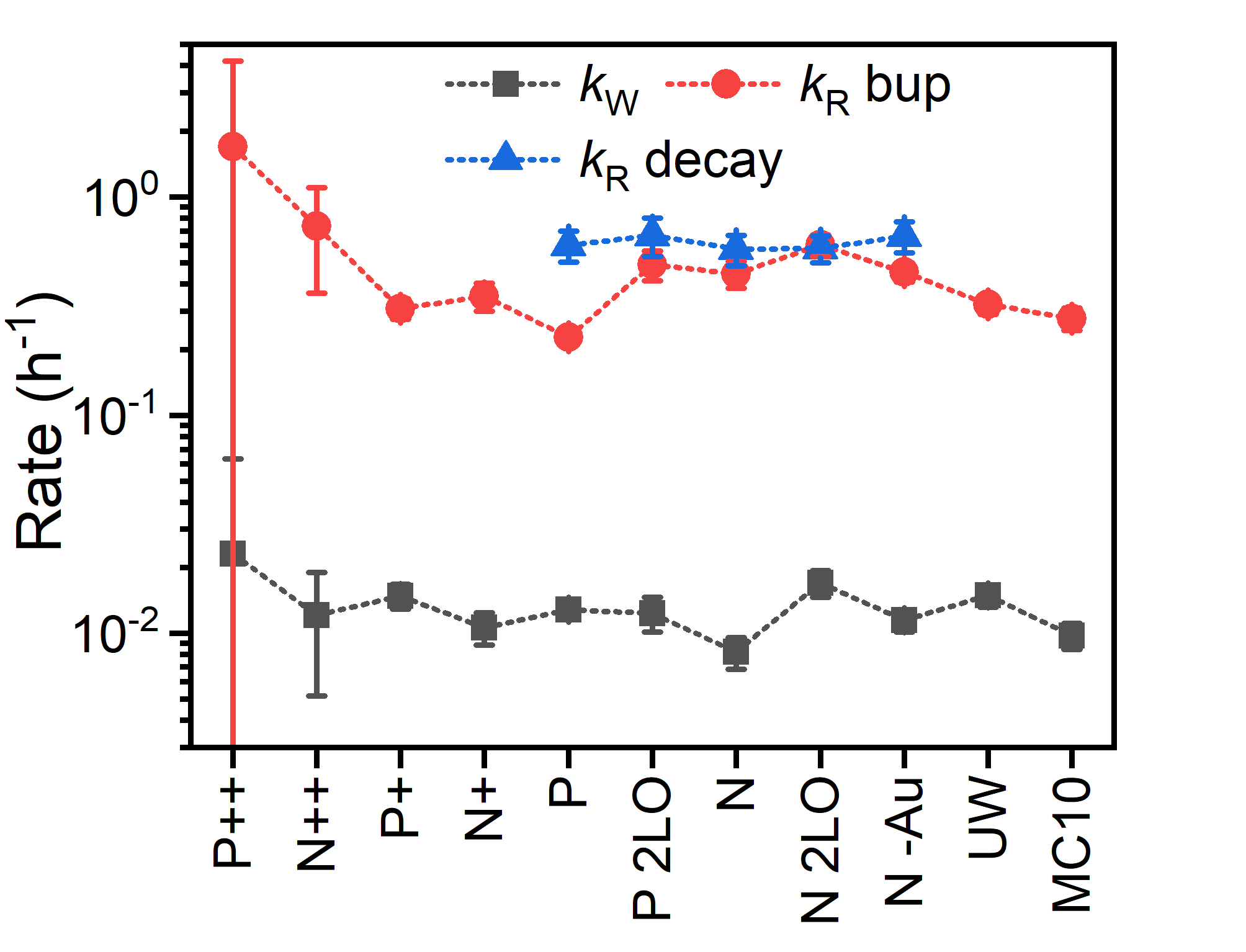

Supplement: NR-016-D4NR02603A-s001 [file NR-016-D4NR02603A-s001.zip › ESI Figs/Supp_Rates3p4T.png]

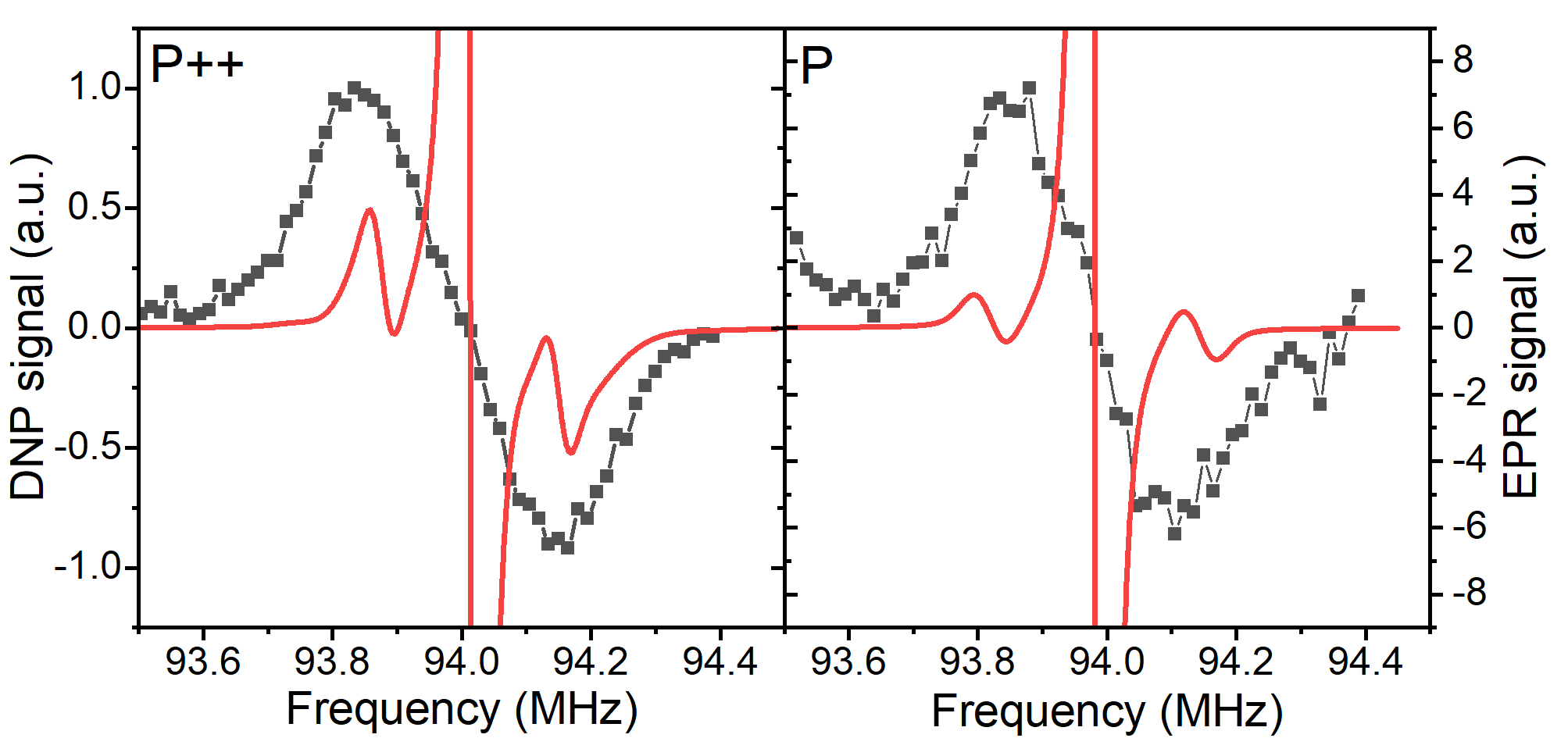

Supplement: NR-016-D4NR02603A-s001 [file NR-016-D4NR02603A-s001.zip › ESI Figs/Supp_Sweep_3p35T.png]

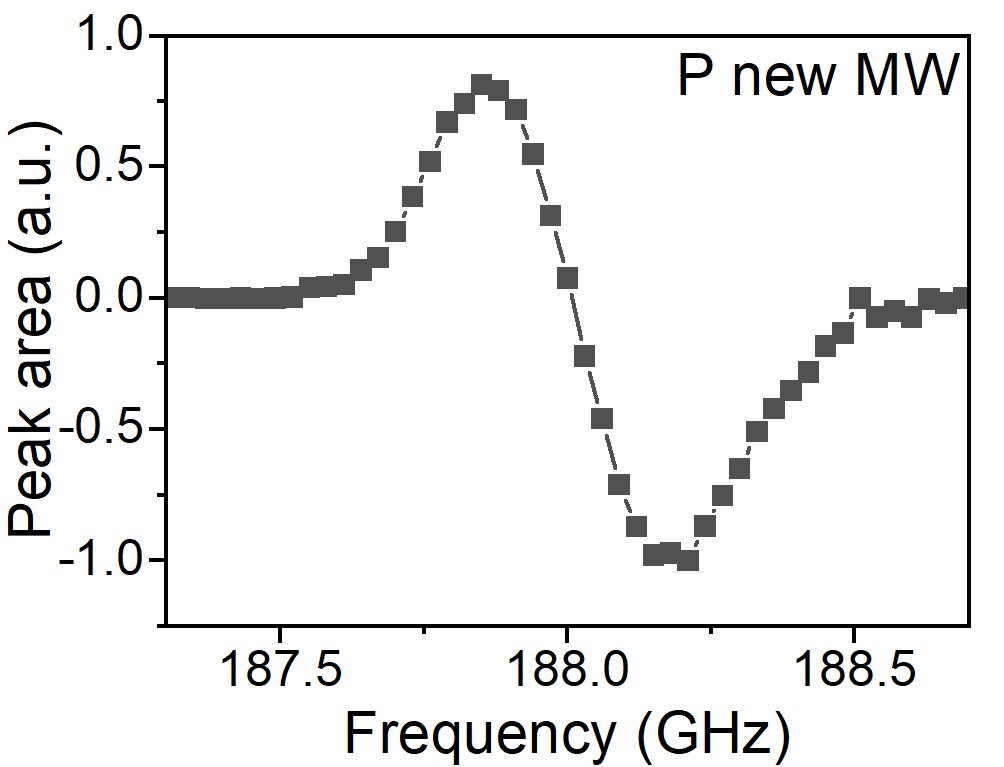

Supplement: NR-016-D4NR02603A-s001 [file NR-016-D4NR02603A-s001.zip › ESI Figs/Supp_Sweep_6p7T_NewMW.png]

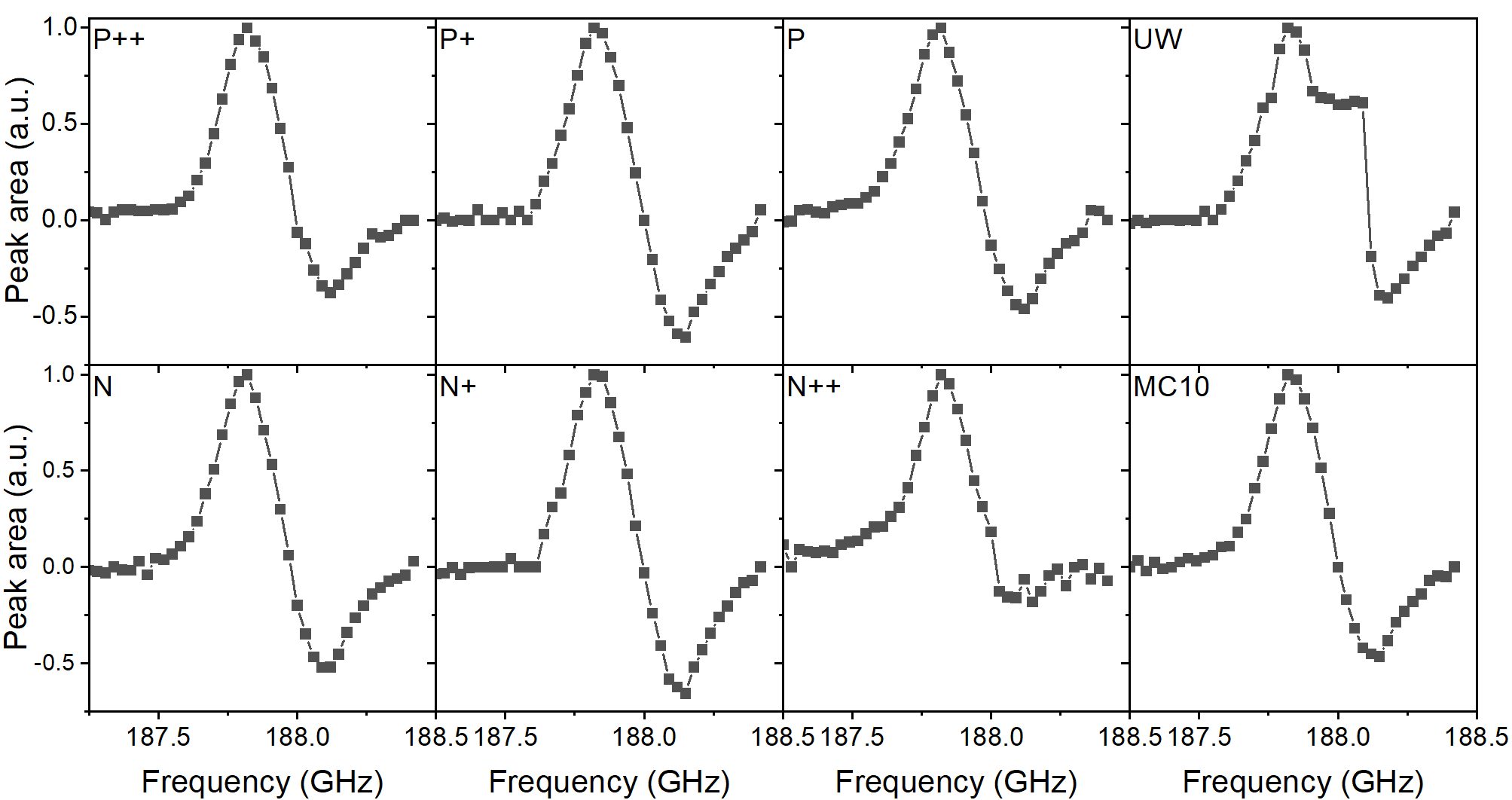

Supplement: NR-016-D4NR02603A-s001 [file NR-016-D4NR02603A-s001.zip › ESI Figs/Supp_Sweep_Grid_6p7T.png]

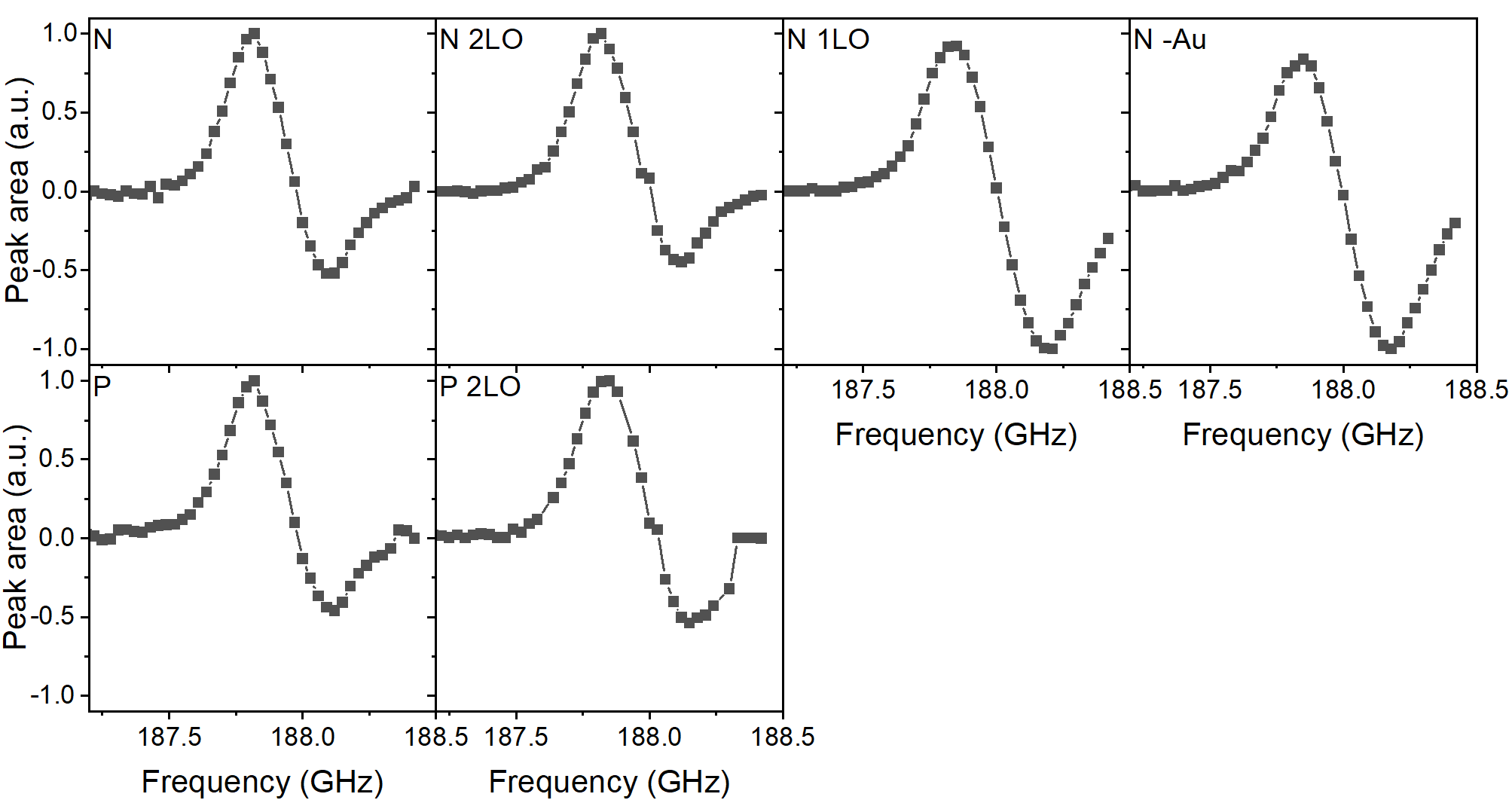

Supplement: NR-016-D4NR02603A-s001 [file NR-016-D4NR02603A-s001.zip › ESI Figs/Supp_Sweep_Oxy_6p7T.png]

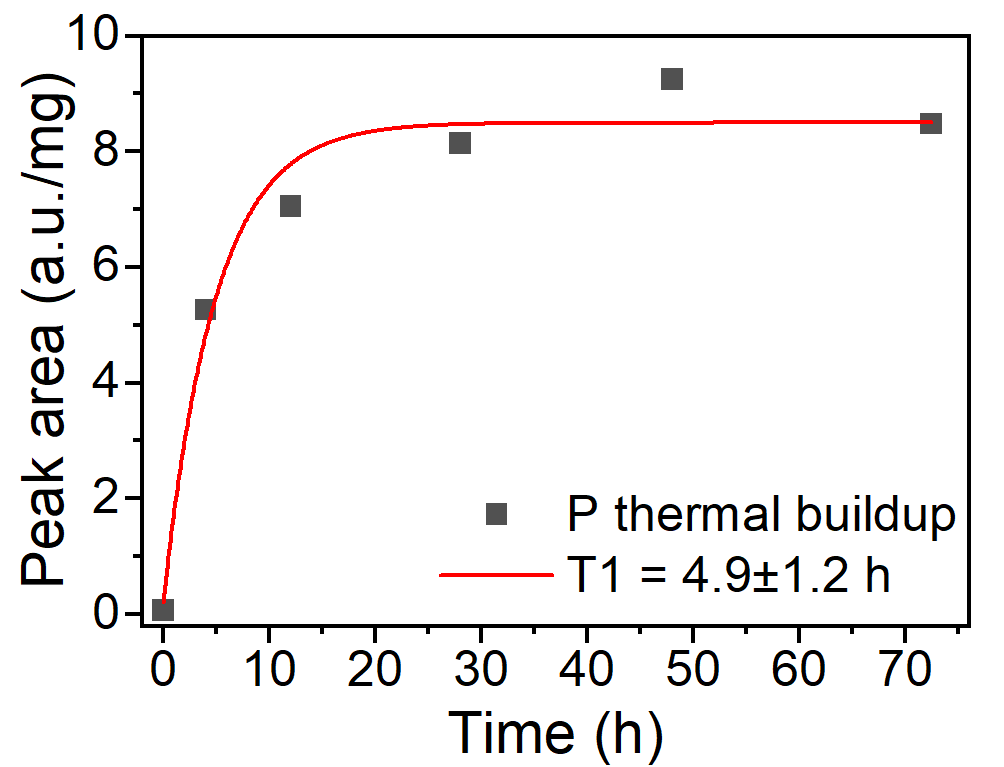

Supplement: NR-016-D4NR02603A-s001 [file NR-016-D4NR02603A-s001.zip › ESI Figs/Supp_Thermal_Bup_6p7T.png]

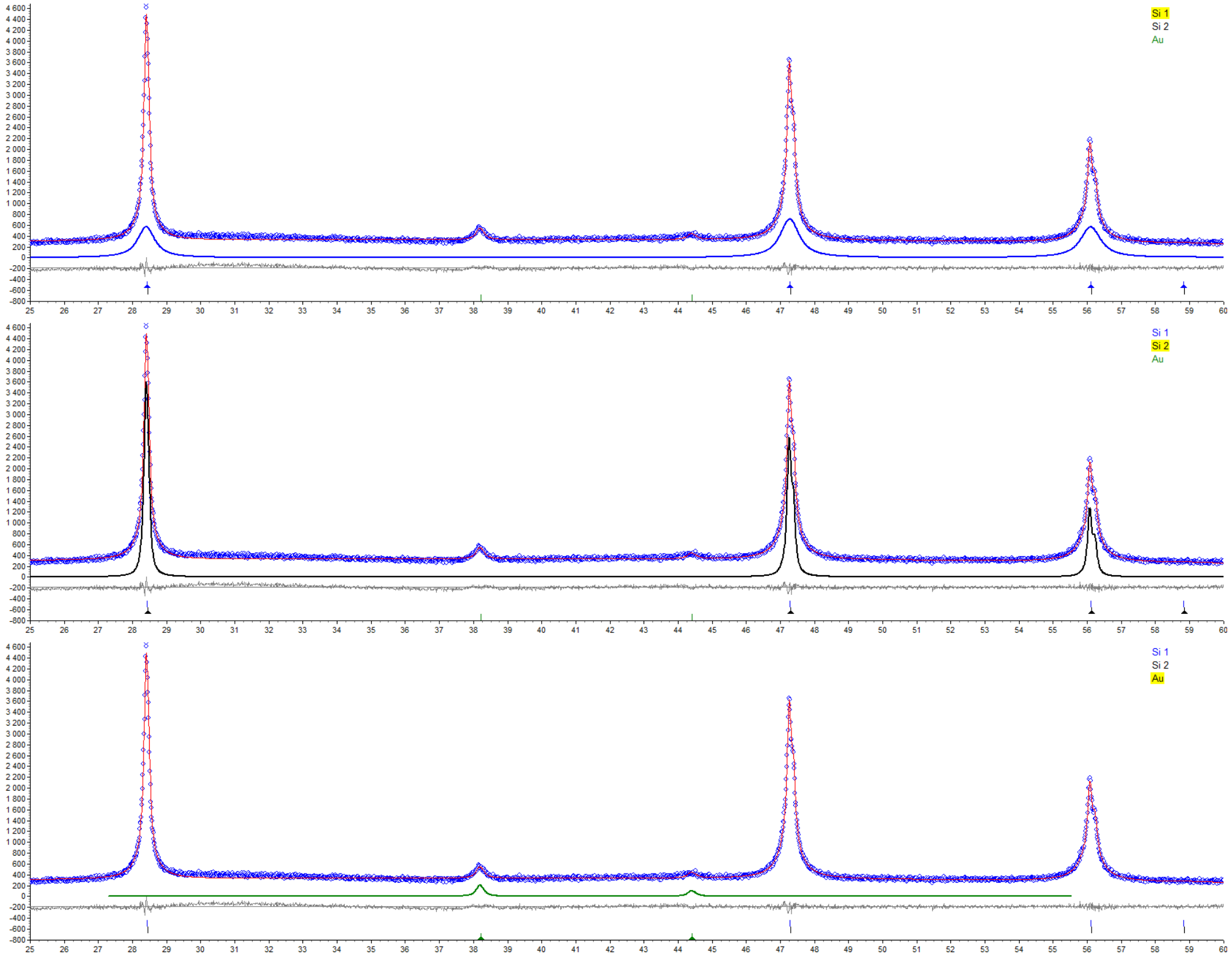

Supplement: NR-016-D4NR02603A-s001 [file NR-016-D4NR02603A-s001.zip › ESI Figs/Supp_XRD_TOPAS.png]
