# Supplementary material for: Controlled synthesis and characterization of porous silicon nanoparticles for dynamic nuclear polarization
Source: Nanoscale. 2024 Sep 18;16(41):19385–99. doi: 10.1039/d4nr02603a (PMC11430043; doi:10.1039/d4nr02603a)
Supplement: NR-016-D4NR02603A-s002 [file NR-016-D4NR02603A-s002.pdf]

Supplementary Information (SI) for Nanoscale.
This journal is © The Royal Society of Chemistry 2024

Supplementary Information for Controlled synthesis and characterization of porous silicon nanoparticles for dynamic nuclear polarization

Gevin von Witte[1,2], Aaron Himmler[2], Viivi Hyppönen[3], Jiri Jäntti[4], Mohammed M. Albannay[1], Jani O. Moilanen[5], Matthias Ernst[2], Vesa-Pekka Lehto[4], Joakim Riikonen[4], Sebastian Kozerke[1], Mikko I. Kettunen[3], and Konstantin Tamarov[4]

[1]Institute for Biomedical Engineering, University and ETH Zurich, Zurich, Switzerland.
[2]Institute of Molecular Physical Science, ETH Zurich, Zurich, Switzerland.
[3]Kuopio Biomedical Imaging Unit, A.I. Virtanen Institute, University of Eastern Finland, Kuopio, Finland.
[4]Department of Technical Physics, University of Eastern Finland, Kuopio, Finland.
[5]Department of Chemistry, Nanoscience Center, University of Jyväskylä, Jyväskylä, Finland.

September 5, 2024

Contents

S1 Experimental **2**
S1.1 Liquid-phase oxidation 2

S2 Characterization of PSi NPs **2**
S2.1 Dynamic light scattering 2
S2.2 Fourier-transform infrared spectroscopy 3
S2.3 X-ray powder diffraction 4
S2.4 Electron paramagnetic resonance 5
S2.5 P_b centers in silicon 11

S3 Dynamic nuclear polarization **12**
S3.1 Thermal polarization buildup 12
S3.2 DNP profiles 13
S3.3 Dynamic nuclear polarization buildup and decay data 16
S3.4 Quantitative analysis of DNP injection and relaxation 21

S4 Density functional theory (DFT) simulations **24**

References **25**

S1 Experimental

S1.1 Liquid-phase oxidation

Two-step and one-step oxidation were performed for PSi NPs (*i.e.*, after milling of thermally oxidized PSi powders) according to the procedure described in ref [1]. In the first step of oxidation, about 100 mg of PSi NPs stored in ethanol suspension were first redispersed in deionized water by repeating two times the following sequence: centrifugation of PSi NP suspension, supernatant removal, redispersion in water in an ultrasound bath (Elmasonic S10). The final redispersion used only 10 ml of water. Next, 10 ml of NH_4OH (7 wt.%, VWR Chemicals) solution was slowly added under stirring followed by slow pouring of 2 ml H_2O_2 (35 wt. %, Acros Organics, Thermo Fisher GmbH). The suspension was then sonicated for 1 min and placed on heating plate and the oxidation reaction proceed for 15 min at 90 °C under stirring. The reaction was then slowed down by diluting the suspension with about 30 ml of water, and the PSi NPs were washed with water by repeating centrifugation-redispersion cycle three times. Again, the final redispersion used only 10 ml of water. In the second step of oxidation, 10 ml of 2 M HCl is added to the NPs under stirring, into which subsequently 2 ml of H_2O_2 (35 wt. %) is poured. The reaction is then carried out at 90 °C for 15 min. Finally, washing is performed as in the first step with the final replacement of water with ethanol for NP storage. One-step oxidation employed only the second step of the two-step liquid-phase oxidation.

S2 Characterization of PSi NPs

S2.1 Dynamic light scattering

Dynamic light scattering (DLS, Zetasizer Nano ZS, Malvern Panalytical) is a simple and fast method to monitor the integral distribution of hydrodynamic sizes as opposed to the direct (and subjective) observation by transmission electron microscopy. Hydrodynamic size is an important parameter to provides the behavior of NPs in biologically relevant media. Herein, DLS was used to follow the hydrodynamic size distributions of PSi NPs during and after the milling of NPs described in Section 2.3 of the main text. Figure S1 shows the size distributions after 1 hour of milling demonstrating the similar hydrodynamic sizes of all the types of PSi NPs used for hyperpolarization.

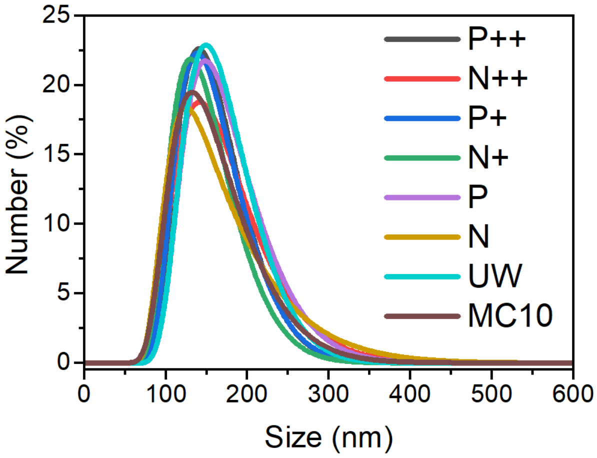

Figure S1: Hydrodynamic size distribution of all the samples after 1 hour milling (see Section 2.3 of the main text).

The top-down approach allows to flexibly alter size distributions by additional milling and centrifugation cycles as required by a specific application. Particularly, for biomedical applications the sizes below 100 nm are preferable[2]. In Figure S2, size distributions of P PSi NPs after centrifugation at 2500 rcf for 20 min show that most of the NPs have sizes below 100 nm. Note that we do not expect any change in the DNP properties of the NPs since the Si crystalline sizes are determined by the pore walls which are

significantly smaller than the hydrodynamic sizes. The use of suspension with relatively large sizes (Figure 1b) made it easier to collect hundreds of milligrams of PSi NPs for the DNP measurements.

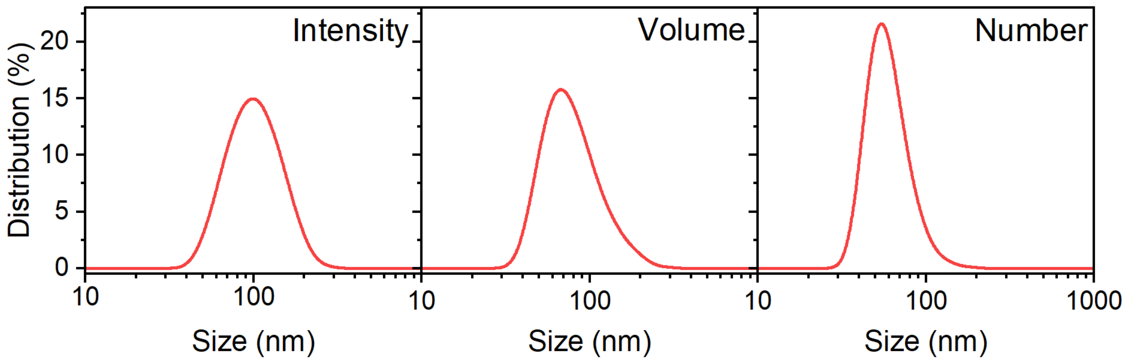

Figure S2: Hydrodynamic size distribution of P PSi NPs after centrifugation with 2500 rcf for 20 min. Intensity is the raw measured signal $\sim r^6$, where r is particle hydrodynamic diameter. Volume ($\sim r^3$) and number ($\sim r^0$) size distributions are calculated from the intensity distribution using the Zetasizer Nano ZS software.

S2.2 Fourier-transform infrared spectroscopy

Surface oxidation of PSi NPs was characterized using Fourier-transform infrared spectroscopy (FTIR, Thermo Nicolet iS50) of KBr tablets. The tablets were prepared by grinding 200 mg of dried KBr powder together with (1–2) mg of dried PSi NPs in a mortar until the powder color became homogeneous. Manual hydraulic press converted the powder into a uniformly colored KBr tablet. FTIR measurements were then performed in transmission mode.

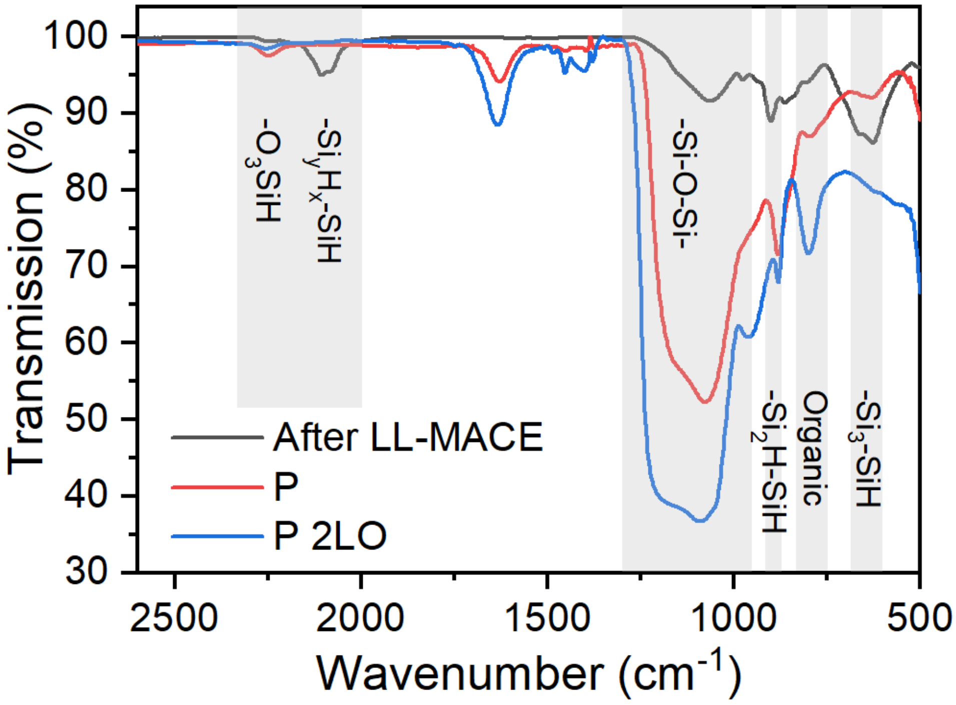

Figure S3: Transmission FTIR spectra of hydrogen-terminated P PSi powder after LL-MACE (dark line), thermally oxidized P PSi NPs (red line), and P PSi NPs after liquid and thermal oxidation (blue line). Grey shaded squares and labels assign FTIR peaks.

Figures S3 and S4 depict the difference between the hydrogen-terminated sample after LL-MACE, milled PSi NPs prepared from thermally oxidized PSi powders, and the liquid-phased oxidized PSi NPs. Hydrogen-terminated sample shows strong IR absorption peaks at $(615–625)\,cm^{-1}$, $948\,cm^{-1}$, and $(2050–2160)\,cm^{-1}$ that correspond to various silicon hydride species on PSi surfaces[1]. The wide peak at $(1000–1250)\,cm^{-1}$ and the peak at $2248\,cm^{-1}$ demonstrate the native oxidation process during overnight drying in an oven at 65 °C. Thermal oxidation with subsequent milling to NPs created the strong Si−O−Si oxide peak with almost complete disappearance of -Si_yH_x-SiH hydride species. However, an appearance of hydrogen bound to backbone oxidized Si was observed ($-O_3SiH$ species) indicating that not all the hydrogen was removed from PSi surfaces. The presence of hydrogen can impede surface functionalization based on reaction with silanes (for example, PEG-silanes[3] or amine-silanes[4]). Therefore, additional liquid-phase oxidation was applied to PSi NPs to further reduce hydride species on NP surfaces, and the influence of oxidation on hyperpolarization was studied. Both two-step and one-step liquid-phase oxidations efficiently reduced the number of -Si_yH_x-SiH although decreasing the gain in ^{29}Si hyperpolarization (see the main text).

Au removal involved highly oxidative iodine solution applied to the N PSi NPs after LL-MACE. The solution effectively oxidized Si surfaces as it can be seen in Fig. S4. The Si oxidative action of the Au etchant was found to be similar to other oxidation types and resulted in nearly full removal of the surface Si-H groups followed by Si backbond oxidation[1].

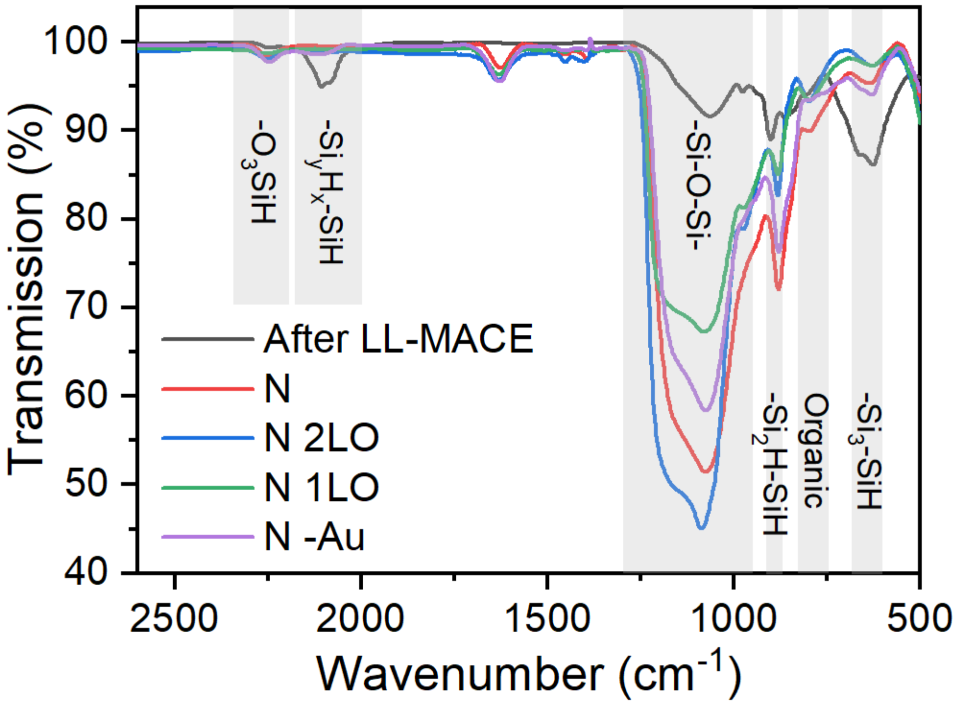

Figure S4: Transmission FTIR spectra of hydrogen-terminated N PSi powder after LL-MACE (dark line), thermally oxidized N PSi NPs (red line), N PSi NPs after two-step liquid and thermal oxidation (blue line), N PSi NPs after one-step liquid and thermal oxidation (green line), and N PSi NPs with Au removed (magenta line). Grey shaded squares and labels assign FTIR peaks.

S2.3 X-ray powder diffraction

Crystalline sizes of PSi particles after LL-MACE were calculated using Retvield refinement in TOPAS® 4.6 software. Typically, three phases were needed to correctly fit a spectrum: two Si phases and one Au phase (Fig. S5). The Si phases corresponded to the pore walls between etch track pores produced by Au NP movement, and to the pore walls between pores produced by remote etching[5, 6].

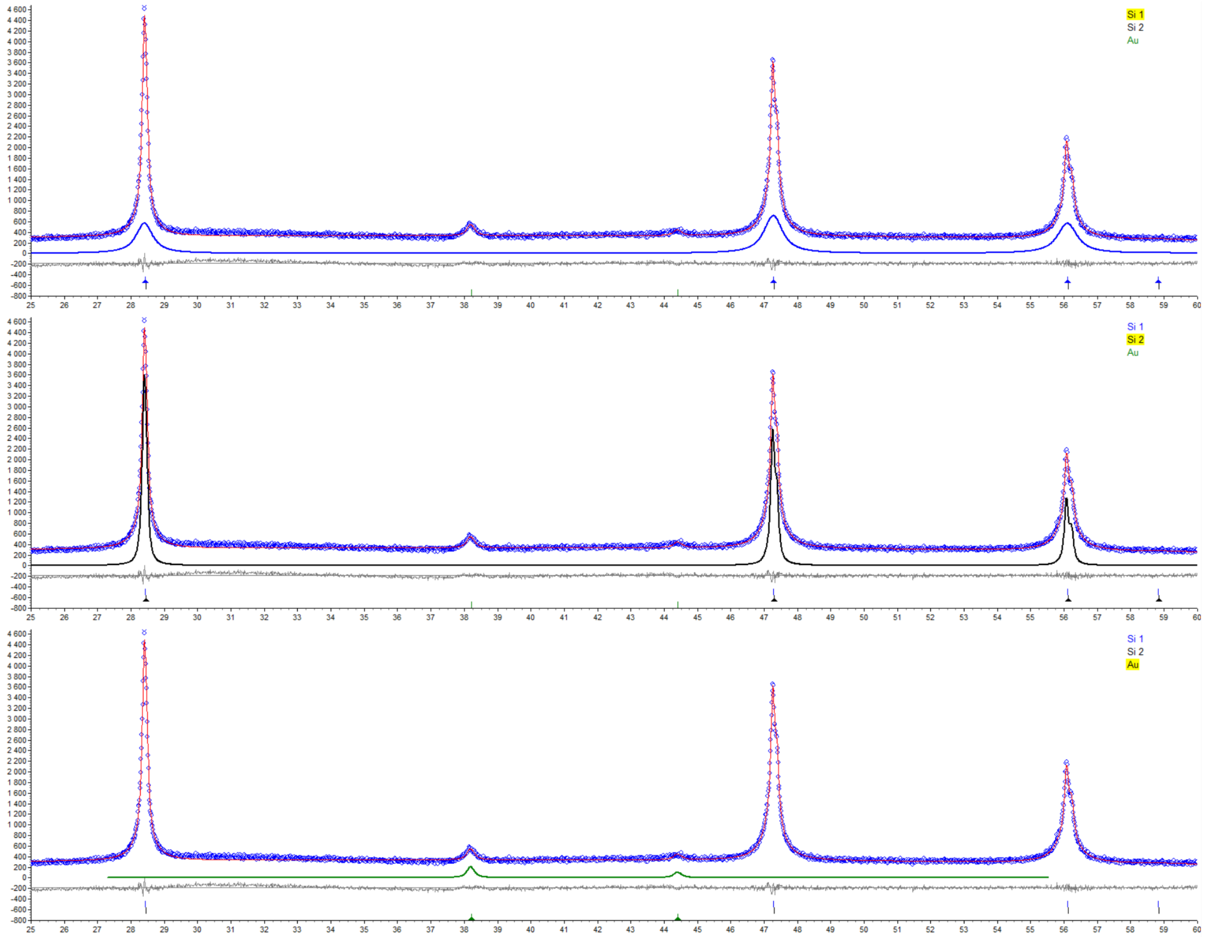

Figure S5: Fitting of XRPD spectrum of P sample with two Si phases and one Au phase. All the samples were processed in the same way.

S2.4 Electron paramagnetic resonance

Electron paramagnetic resonance (EPR) studies were performed using X-band Magnettech MiniScope MS5000 spectrometer operating at room temperature. The same volume of PSi NPs powder was placed in an EPR tube and the tube was placed at the same height for each measurement. The mass and surface number of paramagnetic centers was calculated by double integration of spectra with subsequent comparison with a TEMPO sample with a known number of radicals. The mass and surface amount of the paramagnetic centers is summarized in Table S1. The concentration of the centers per unit of mass was comparable for all the samples and ranged from $(4.4\pm0.4)\cdot10^{15}$ mg^{-1} for N+ to $(6.3\pm0.6)\cdot10^{15}$ mg^{-1} for N 1LO. The surface density for P++ and N++ samples was about 3 times smaller than for other samples due to their higher surface area (Fig. 1d) while the number of centers per unit mass remained roughly the same.

EPR spectra of thermally oxidized PSi NPs of different Si types and different additional liquid oxidations are presented in Figs. S6 and S7, respectively. All spectra represent a powder average of paramagnetic dangling bond P_b centers that are in turn randomly oriented and located at different Si crystalline planes in Si/SiO_2 interface[7]. Hyperfine satellite peaks at $\Delta B = (5.8\text{–}7.7)$ mT unambiguously demonstrated the presence of ^{29}Si nuclei at the central P_b position (Fig. S8). The hyperfine constants $A = (325\text{–}431)$ MHz coincide well with $A_{\|} = 210$ MHz and $A_{\perp} = 417$ MHz for the (111) P_b center[8]. The superhyperfine interaction typically observable for planar (111) P_b center at about 0.8 mT or 45 MHz[9] could not be resolved due to high peak broadening in our samples but was assumed to be present.

Following the discussion of P_b centers in the main text, the measured EPR spectra were fitted with EasySpin 5.2.35[10] using a combination of P_b^{iso} and (111) P_b, since these centers are assumed to be the dominant ones in thermally oxidized porous Si[11]. The inclusion of the (111) P_b center in the fitting was the most obvious for the standard thermally oxidized porous Si sample prepared by the

Table S1: Summary of the experimental EPR data. The number of P_b centers was calculated from the known paramagnetic center concentration of TEMPO radical and additionally confirmed using thermally oxidized electrochemically etched PSi sample[1].

Sample	Calculated from TEMPO sample: P_b centers, $\cdot 10^{15}$ mg^{-1}	Calculated from TEMPO sample: P_b centers, $\cdot 10^{12}$ cm^{-2}	Experimental data: EPR lwpp[a], mT	Experimental data: EPR FWHM[b], mT
PSi	4.6 ± 0.4	1.9 ± 0.1	1.10	1.13
P++	5.4 ± 0.3	2.7 ± 0.1	0.76	1.03
P+	4.5 ± 0.4	5.0 ± 0.5	0.55	0.92
P	4.8 ± 0.3	5.3 ± 0.3	0.57	0.93
P 2LO	5.3 ± 0.3	5.9 ± 0.4	0.55	0.90
UW	5.2 ± 0.3	4.9 ± 0.3	0.55	0.90
N	5.6 ± 0.4	6.1 ± 0.5	0.55	0.90
N 2LO	5.4 ± 0.2	5.8 ± 0.3	0.55	0.92
N 1LO	6.3 ± 0.6	6.8 ± 0.7	0.55	0.90
N -Au	4.9 ± 0.4	5.2 ± 0.4	0.53	0.87
N+	4.4 ± 0.4	4.1 ± 0.4	0.55	0.94
N++	4.7 ± 0.4	1.8 ± 0.1	0.60	0.95
MC10	5.5 ± 0.3	3.1 ± 0.2	0.60	0.96

[a] peak-to-peak linewidth [b] full width at half maximum

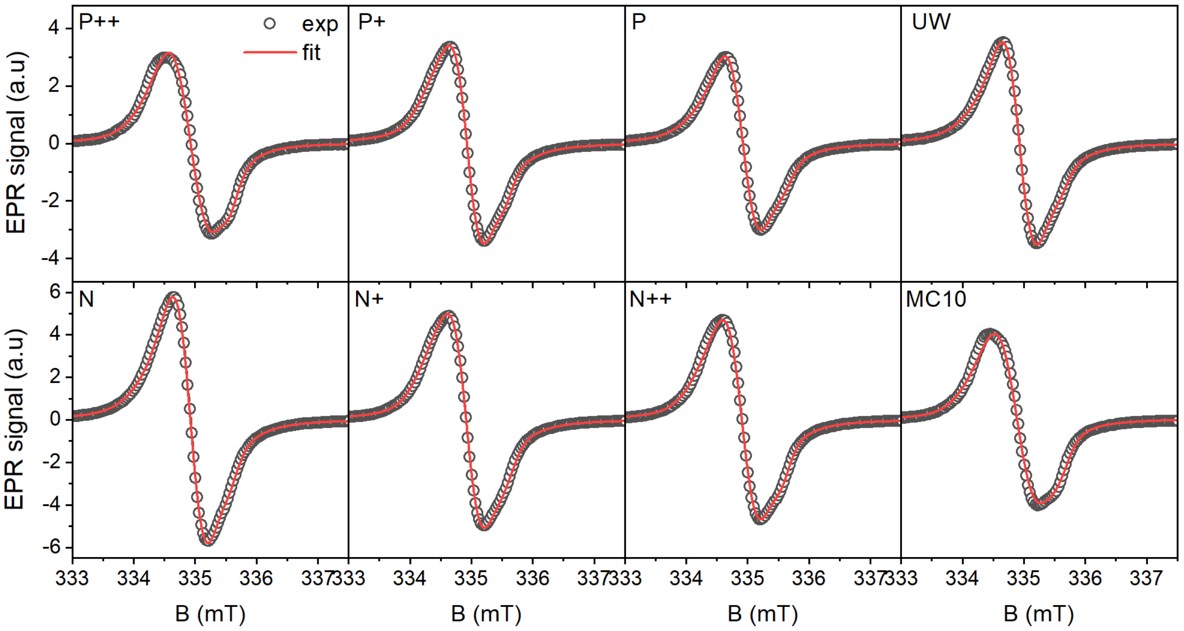

Figure S6: Electron paramagnetic resonance spectra of thermally oxidized PSi NPs of different Si types. The experimental data (dark lines) was fitted (red lines) as discussed in the text.

conventional electrochemical anodization of (100) P++ Si wafer (Fig. S9)[12] with subsequent thermal oxidation. During the electrochemical anodization, the etched pores are formed normal to the (100) surface, which results in more pronounced signal from (111) P_b centers compared to much less ordered pores in the LL-MACE samples. Nevertheless, even in the LL-MACE samples the anisotropy of the EPR spectrum at about 336 mT is due to the presence of (111) P_b centers (Fig. S6 and S7).

The resulting fitting parameters for the anodized PSi sample give reasonable values. The weights for the P_b and P_b^{iso} components are 0.37 and 0.63, respectively, which show the presence of relatively high fraction of well-defined (111) P_b centers. As it is expected, the g-factor strain for $\mathbf{B} \perp [111]$ is much higher than for $\mathbf{B} \parallel [111]$ with the strain values close to the ones measured for planar (111) P_b

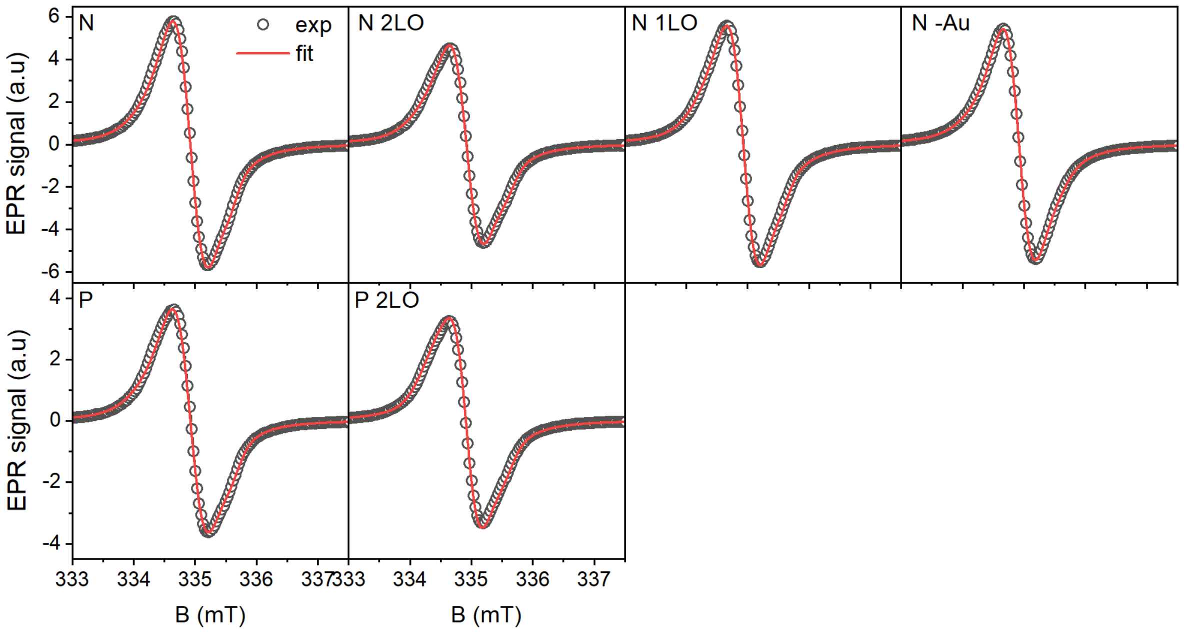

Figure S7: Electron paramagnetic resonance spectra of thermally oxidized P and N PSi NPs, after additional two-step (2LO) or one-step (1LO) liquid oxidation, or PSi NPs prepared by milling N PSi powder oxidized by gold etchant. The experimental data (dark lines) was fitted (red lines) as discussed in the text.

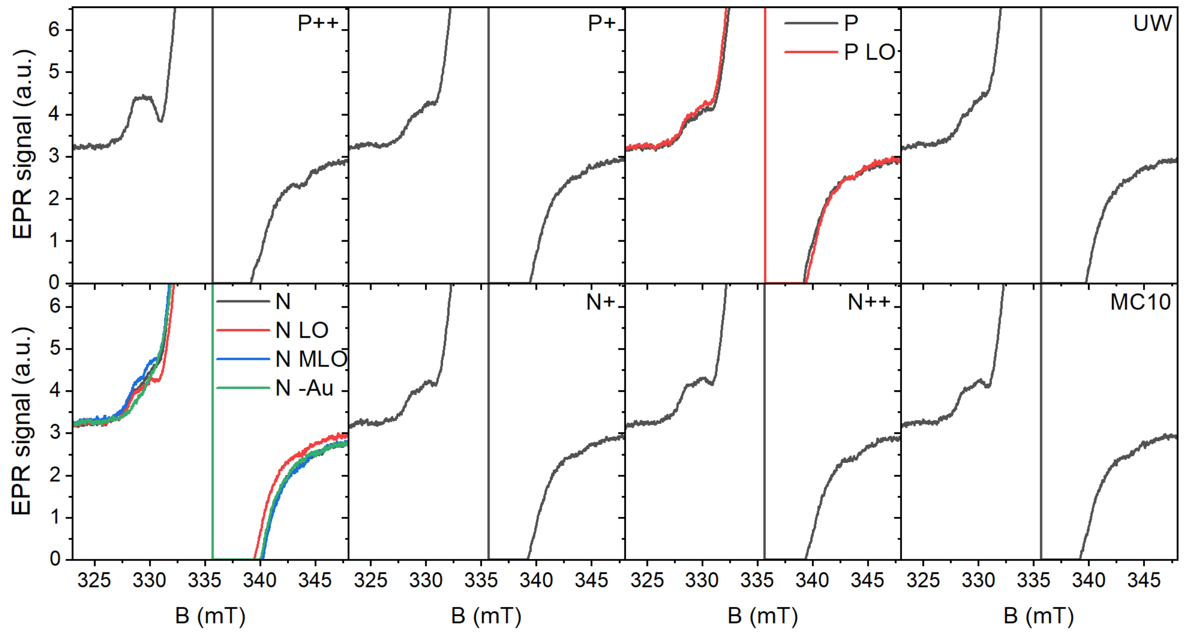

Figure S8: Electron paramagnetic resonance spectra of all the samples depicting peaks for hyperfine interaction of electron spin with the central ^{29}Si nuclei. The superhyperfine interaction with the backbond ^{29}Si nuclei is invisible due to large width of P_b^{iso} spectra.

center[13]. The Gaussian and Lorentzian peak-to-peak linewidths for the P_b are $\Delta B_{pp}^{G} = 0.045\,\text{mT}$ and $\Delta B_{pp}^{L} = 0.16\,\text{mT}$, respectively [14, 15] (Table S2). These linewidths closely match the values evaluated by Stesmans et al.[14, 15] during their study of dipolar interaction between (111) P_b and its influence on the low-temperature EPR spectra. Indeed, they found $\Delta B_{pp}^{L} \approx 0.16\,\text{mT}$ for $[P_b] \approx 7 \cdot 10^{12}\,\text{cm}^{-2}$. With the weight decrease of the P_b centers in LL-MACE samples, the P_b fitting becomes less straightforward and the fitting parameters start to deviate from the ones for the planar P_b centers. This is expected due to high structural irregularity of the samples' porous surfaces.

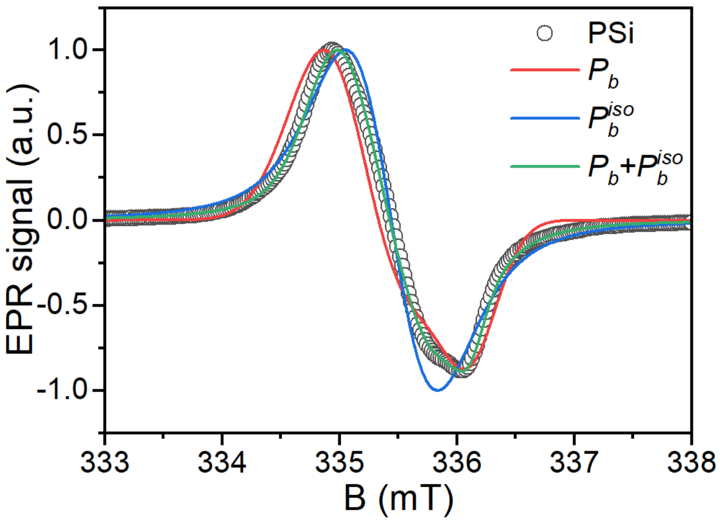

Figure S9: Fitting of the experimental EPR spectra of TOPSi sample (open dark circles) with powder pattern of (111) P_b centers (red line), P_b^{iso} centers (blue line), and combination of the two centers (green line). Obtained weights for P_b and P_b^{iso} were 0.37 and 0.63, respectively. The g strain for P_b defect was $\Delta g_{\perp} = 0.00296$ and $g_{\|} = 0.0005$.

Compared to P_b centers, P_b^{iso} was described by phenomenological Gaussian ΔB_{pp}^{G} and Lorentzian ΔB_{pp}^{L} peak-to-peak linewidths. They were found to be of the same order of magnitude in the range of $(0.1–0.5)$ mT, which constituted the total linewidth of ≈ 0.6 mT and corresponded to the average linewidth of randomly oriented P_b centers found on different crystalline planes[13, 16]. The Gaussian part in the linewidth was assumed to come from the g-factor strain that was not included as an additional fitting parameter for P_b^{iso}, while the Lorentzian part showed even larger values than the ones that take into account dipolar interaction induced broadening [14, 15] (Table S2). We were not able to find a feasible explanation for such a large broadening from the porous Si literature. One possible explanation is the clustering of P_b centers due to the irregularity of the porous surface in a similar way it was demonstrated by LOD-EPR for partially amorphous Si sample[17]. Overall, it is reasonable to assume the presence of dipolar interaction in our samples with similar $[P_b]$ or higher concentrations compared to Stesmans et al.[14, 15] (Table S1).

The results of EasySpin simulation of the EPR spectra were then used to calculate the EPR spectra at the DNP conditions. For this, the best fit models for each sample were fed to EasySpin to simulate powder pattern structure representing the high-field frequency-swept experimental conditions. All the high field spectra looked similar and, therefore, only the ones for P sample are presented (Fig. S10). Similar to the X-band EPR, the high-field spectra show the strong central peak and the two weak satellite peaks, which correspond to the P_b centers located on the central ^{28}Si and ^{29}Si atoms, respectively. We highlight the slight shift towards higher frequency of the strongest EPR peak compared to zero DNP frequency (Fig. S3.2) possibly due to slightly lower experimental magnetic field strength than 6.7 T used for the EPR simulation.

As a final remark, conduction band electrons with $g = 1.9995$ have been observed in heavily doped n-type porous Si and p-type porous Si under illumination at 4.2 K [18, 19]. It is, however, not possible to identify conduction band electrons in our samples. Although the fitting of EPR does give the g-factor close to 1.9995 (Fig. S11), the peak width is at least three times larger than 0.1 mT measured by Young et al.[18, 19] Thus, it is concluded that neither conduction band electrons nor the phosphorus donor electrons could be identified.

Table S2: EasySpin simulation results of the experimental EPR data. The fitting was performed according to the mixture of anisotropic $P_b^{(111)}$ and isotropic P_b^{iso} centers. Anisotropic centers were fitted with $g_{\|} = 2.00185$, $A_{\|} = 230 \pm 25$ MHz and $g_{\perp} = 2.0081$, $A_{\perp} = 420 \pm 15$ MHz with g-strain[8] and Lorentzian line broadening[15] to include g-factor stain and P_b dipolar interaction, respectively. P_b^{iso} centers were fitted with Voigtian lineshape to include homogeneous and inhomogeneous line broadening effects due to strain and dipolar interaction.

Sample	$P_b^{(111)}$ $g_{\perp} \cdot 10^{-3}$ strain	$g_{\|} \cdot 10^{-3}$ strain	ΔB_{pp}^{L}, mT	weight, %	P_b^{iso} g-factor	ΔB_{pp}^{G}, mT	ΔB_{pp}^{L}, mT	weight, %
PSi	3.0	0.1	0.0012	13	2.0055	0.69	0.28	87
P++	5.4	1.3	0.0041	11	2.0054	0.46	0.37	89
P+	3.7	1.3	0.0001	9	2.0054	0.13	0.49	91
P	5.9	1.7	0.0009	11	2.0055	0.24	0.45	89
P 2LO	3.1	0.1	0.0014	11	2.0054	0.00	0.49	89
UW	5.9	1.6	0.0014	8	2.0055	0.21	0.45	92
N	5.6	1.4	0.0006	8	2.0054	0.23	0.45	92
N 2LO	5.2	1.8	0.0017	9	2.0055	0.17	0.47	91
N 1LO	4.3	1.6	0.0018	7	2.0054	0.12	0.48	93
N -Au	5.5	1.9	0.0027	4	2.0054	0.13	0.47	96
N+	3.6	0.1	0.0003	13	2.0054	0.19	0.47	87
N++	4.7	1.6	0.0009	12	2.0055	0.26	0.45	88
MC10	5.2	0.6	0.0022	15	2.0056	0.47	0.37	85

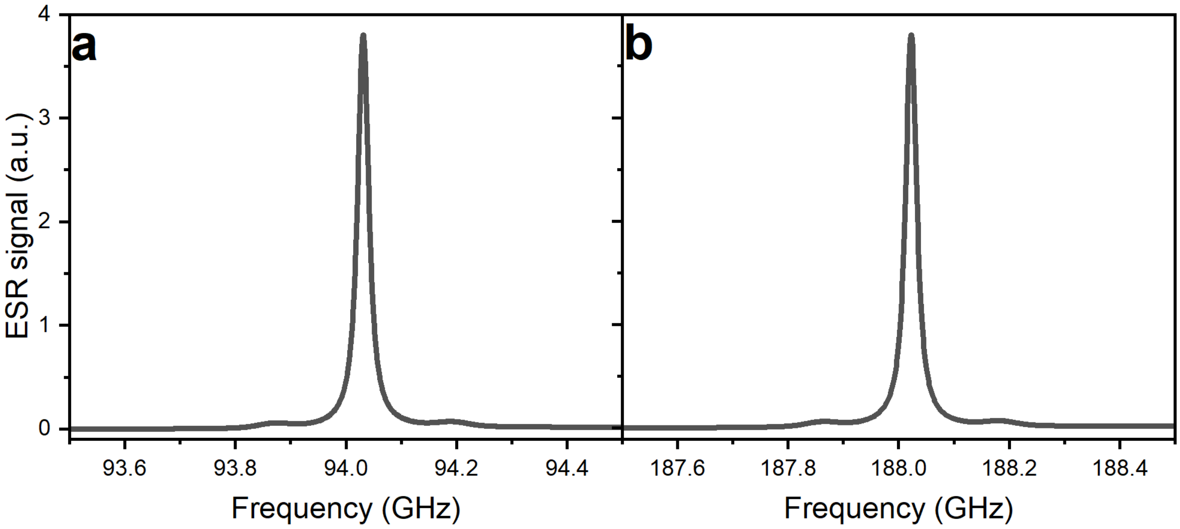

Figure S10: Simulation of EPR spectra for P sample at 3.3451 T (a) and at 6.6919 T (b). Simulation has been done using EasySpin after the best (111) P_b and P_b^{iso} system was obtained from fitting of the experimental EPR data (Fig. S6). The magnetic field values were calculated from the spectrometer frequency.

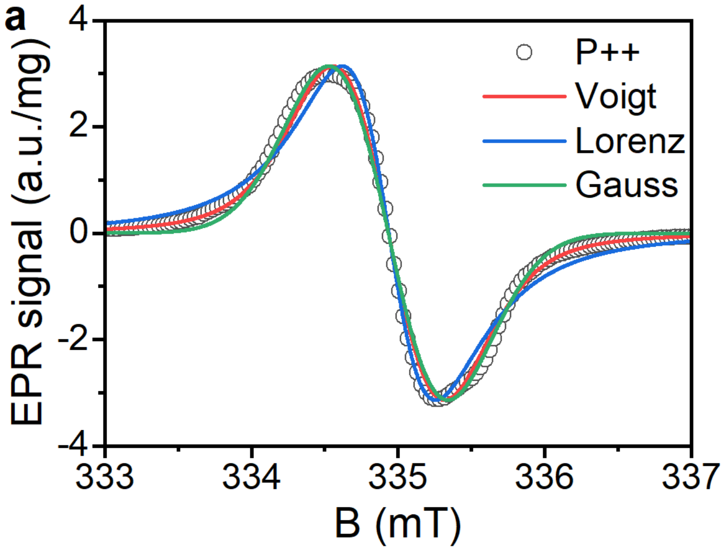

Figure S11: Fitting of the EPR spectrum of P++ PSi NPs with two pseudo-Voigt lines (red line). The first and second Voigt lines give g-factors of 2.0054 and 1.9991, respectively. The EPR spectrum was obtained by integrating the corresponding EPR spectrum. The FWHM for $g = 1.9991$ peak is 0.4 mT.

S2.5 P_b centers in silicon

P_b centers have been widely investigated by EPR both on atomically flat specific crystalline planes and in porous Si because of their importance in metal-oxide-semiconductor devices[20, 21] and to elucidate their influence on photoluminescent properties of porous Si[7, 22–24]. Different types of P_b centers have been identified with some ambiguity in their naming. Following Brower[9], a P_b center is a localized dangling bond of a Si atom backbonded to three Si atoms at the Si/SiO_2 interface on the (111) crystalline plane ($Si_3 \equiv Si\cdot$). We denote this center as $P_b^{(111)}$ for clarity. P_{b0} and P_{b1} centers are two distinct centers on the oxidized (100) plane with axial and rhombic symmetries[25], respectively. Due to structural similarity of $P_b^{(111)}$ and P_{b0} centers, quite commonly these centers are interchangeably denoted as P_b or P_{b0} in the published literature *e.g.*, in Refs. [13, 20].

The trigonal symmetry of $P_b^{(111)}$ centers dictates the trigonal symmetry of the g-factor and HF tensor resulting in $g_{\|} = 2.00185$, $A_{\|} = 230 \pm 25\,\text{MHz}$ and $g_{\perp} = 2.0081$, $A_{\perp} = 420 \pm 15\,\text{MHz}$ as determined by angular resolved EPR[13]. The line shape has been found to vary depending on the orientation of the [111] crystalline direction with respect to the external magnetic field. The EPR line has Lorentzian shape with a peak-to-peak line width $\Delta B_{pp}^{L} = 0.22 \pm 0.015\,\text{mT}$ for $\mathbf{B} \parallel [111]$ and Gaussian shape with $\Delta B_{pp}^{G} = 0.82 \pm 0.05\,\text{mT}$ for $\mathbf{B} \perp [111]$ indicating the presence of $g_{\perp}$ strain with $\Delta g \approx 0.0045$. Hyperfine (HF) interaction with the nearest (backbonded) neighbor ^{29}Si nuclei has also been resolved with a HF constant $A_{2\text{n}} = 41.5 \pm 0.5\,\text{MHz}$. Note, that in the literature $A_{2\text{n}}$ is denoted as superhyperfine interaction in some cases[26].

The analysis of the HF tensor in terms of one-electron molecular orbitals[9] gave a 12% s-like and 88% p-like wave function character with around 80% of the total spin-density localized on the central Si· atom. The spin density distribution together with the large HF interaction results in a large Fermi-contact interaction compared to the dipolar part of the HF interaction: The dipolar HF interaction is up to $\sim 65\,\text{MHz}$ and $\sim 1.5\,\text{MHz}$ for the central and the nearest neighbor nuclei, respectively. Further from the nearest neighbors, the HF interaction is supposed to be governed by the dipolar part, which decreases rapidly with distance. For a ^{29}Si at a distance of two lattice constants away from the P_b center, the estimated dipolar HF interaction is $\sim 10\,\text{kHz}$.

In oxidized porous Si films, X-band EPR (9 GHz) performed at room temperature[11, 20, 22, 24, 27, 28] and $(4\text{–}20)$ K [16, 18, 29] found two general classes of P_b centers depending on the oxidation conditions. The first class has been observed in both (100)- and (111) crystallographic planes of porous Si oxidized under controlled oxygen, hydrogen and moisture content. It is reminiscent of $P_b^{(111)}$, P_{b0}, P_{b1} centers found on the corresponding oxidized crystalline planes[9, 13–15, 20, 21, 30]. Among these, the dominant center is the $P_b^{(111)}$ due to the simultaneous presence of four possible interfaces (111), $(1\bar{1}1)$, $(1\bar{1}\bar{1})$, $(\bar{1}\bar{1}1)$[11]. This center exhibits similar axial symmetry, g-factors and HF constants, s and p spin densities as the $P_b^{(111)}$ center on the corresponding crystalline plane[11, 27]. Highlighted difficulties to detect (100) P_{b0} and P_{b1} centers[11, 31, 32] have been attributed to the dominance of $P_b^{(111)}$ center and to the reconstruction of (100) P_{b0} centers in porous Si[24] (reconstruction is not efficient on a planar (100) Si surface[22]). Therefore, the measured EPR spectra in controllably oxidized porous Si closely follows the features of crystalline Si samples including angular dependence of g-factors and line widths[11]. Furthermore, EPR spectra from oxidized porous Si are comparable with the spectra obtained at K- (24 GHz) and Q-band (35 GHz) at room and $(1.4\text{–}20)$ K[9, 13–15] on the planar Si surfaces if spectra were acquired under non-saturating conditions. At liquid He temperatures, low MW powers are required due to strong saturability and long $T_{1\text{e}}$ times up to approximately 80 ms[33].

The second class of P_b centers in porous Si develops under uncontrolled native[22] or thermal oxidation in air[18, 23, 28], and during thermal annealing[7, 27, 28]. This P_b^{iso} center is characterized by isotropic $g = 2.0055$. Despite the g-factor is isotropic, the linewidth can retain anisotropy which follows the trigonal structure similar to the $P_b^{(111)}$ center with the smallest value of $0.6\,\text{mT}$ for $B \parallel [100]$ and the largest value of for $1.2\,\text{mT}$ $B \parallel [111]$[16, 22]. Compared to P_b^{iso}, the P_b centers formed on a corresponding Si plane thus have much narrower line widths. Electron spin relaxation times of P_b^{iso} have received less attention and, therefore, are compared to commercial samples previously investigated for DNP[34], although they might have substantial amount of paramagnetic amorphous Si centers[35]: The measured $T_{1\text{e}}^{\text{slow}}$ and $T_{2\text{e}}$ show rather similar values to $P_b^{(111)}$ equal to $(10\text{–}70)$ ms and $(0.1\text{–}2)$ µs at 10 K, respectively.

The measured EPR spectra of our samples are represented by the relatively broad lines with noticeable asymmetry (Fig. 1f and S6, Suppl. Inf.). The total surface densities of the P_b centers are in the range

of $(1.8–6.8) \cdot 10^{12}\,\mathrm{cm}^{-2}$ (Fig. 1g) leading to a $1.9 - 3.7\,\mathrm{nm}$ average distance between them. According to EasySpin[10] fitting (Section S2.4, Suppl. Inf.), P_b centers in the oxidized LL-MACE samples are represented by high number of P_b^{iso} and few $P_b^{(111)}$ centers. For planar $P_b^{(111)}$ centers, the fitted line broadening values indicate the presence of g-strain typical for such centers ($\Delta g \approx 0.0047$)[13] . This strain contributes to the Gaussian peak-to-peak line width $\Delta B_{pp}^{G} \approx 0.8\,\mathrm{mT}$, similar to planar $P_b^{(111)}$ strain. The Lorentzian part below $3\,\mu\mathrm{T}$ is much lower than obtained by Stesmans and Gorp[14, 15] ($\Delta B_{pp}^{L} \approx 0.16\,\mathrm{mT}$, Table S2, Suppl. Inf.), indicating the possible isolation of the $P_b^{(111)}$ centers.

P_b^{iso} centers are fitted with a phenomenological Voigtian lineshape, which gave $\Delta B_{pp}^{G} = (0.12–0.47)\,\mathrm{mT}$ and $\Delta B_{pp}^{L} = (0.37–0.49)\,\mathrm{mT}$. The smaller ΔB_{pp}^{G} is possibly due to less strain for the P_b^{iso} than for the $P_b^{(111)}$, while the high value ΔB_{pp}^{L} could indicate stronger dipolar coupling compared to $P_b^{(111)}$. $\Delta B_{pp}^{L}(P_b^{\mathrm{iso}})$ corresponds to $T_2(P_b^{\mathrm{iso}}) \approx 30\,\mathrm{ns}$. It is possible that the large ΔB_{pp}^{L} for the P_b^{iso} may indicate the clustering of P_b^{iso} centers with orders of magnitude faster electron spin-lattice relaxation rate than for the standalone centers. The investigation of clusters requires further (pulsed) EPR studies to detect the spin-lattice relaxation.

HF interaction with the central ^{29}Si atom was also clearly identified in our X-band EPR measurements (Fig. S8, Suppl. Inf.). The HF constants are in the range of $(11.6–15.4)\,\mathrm{mT}$ or $(325–431)\,\mathrm{MHz}$ and correspond to the typical values of the (111) P_b center with $A_{\parallel} = 210\,\mathrm{MHz}$ and $A_{\perp} = 417\,\mathrm{MHz}$[13]. HF coupling with the nearest neighbor (backbonded) ^{29}Si nuclei was not observed due to the large broadening of P_b^{iso} but could be assumed to be present with $A_{2\mathrm{n}} \approx 42\,\mathrm{MHz}$[9]. On the other hand, it could be possible that the HF interaction with backbonded ^{29}Si is diminished in our samples due to the backbond oxidation[1] of the central ^{28}Si atom. In the case of backbond oxidation, HF interaction with a distant ^{29}Si nuclei can be assumed to be of a purely dipolar nature and scale as r^{-3} with r the distance from a P_b center.

The average distance between the P_b centers assuming their uniform distribution is $d_{\mathrm{ee}} = (1.9–3.7)\,\mathrm{nm}$ deduced from their amount per surface area. This distance gives the estimated dipolar coupling D_{ee} on the order of $D_{\mathrm{ee}} = (1.0–7.4)\,\mathrm{MHz}$, which is about $(1 - 10)$ times lower than the homogeneous line broadening calculated from ΔB_{pp}^{L} for the P_b^{iso} centers (Tbl. S2, Suppl. Inf.). Such a discrepancy between D_{ee} and ΔB_{pp}^{L} may indicate clustering of the P_b centers on the ridges and edges of the irregular pore walls and pore openings. Nevertheless, ΔB_{pp}^{L} clearly correlates with the D_{ee} and surface density of P_b centers with the correlation coefficient of ~ 0.63 demonstrating a consistent increase of dipolar interaction with the decrease of their mutual distance. For the $P_b^{(111)}$ centers, $\Delta B_{pp}^{L} \approx (0.001–0.004)\,\mathrm{mT}$, which is more than two orders of magnitude smaller than ΔB_{pp}^{L} for P_b^{iso} centers, might indicate a relative isolation of the $P_b^{(111)}$ centers from other P_b centers.

S3 Dynamic nuclear polarization

S3.1 Thermal polarization buildup

The polarization enhancements and absolute polarizations were calculated by integrating the pseudo-Voigt fits of FFT-processed FID data. The integrated values were then divided by the thermal polarization signal processed the same way and taken after 72 h of polarization inside a polarizer with microwave radiation switched off (Fig. S12).

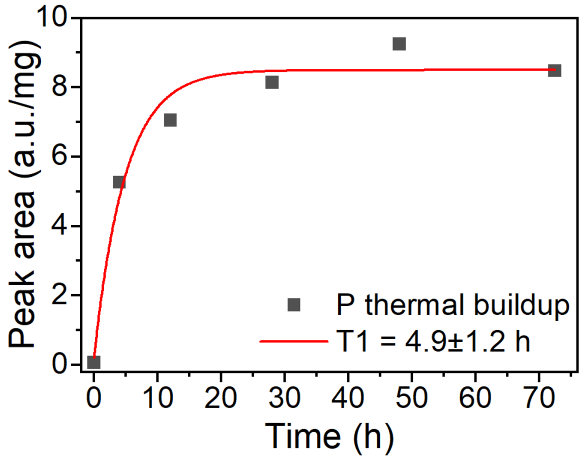

Figure S12: Thermal polarization buildup for the P sample at 6.7 T and 1.4 K.

S3.2 DNP profiles

The normalized DNP profiles (sweep spectra) at 6.7 T and 1.4 K for thermally oxidized PSi NPs are depicted in Fig. S13. There are minor differences between the Si types in the asymmetry of positive and negative peak values. This asymmetry was attributed to the slight difference of surface area induced by increased remote etching for highly doped Si[6], and the corresponding possible change in the structure of P_b centers.

In all the spectra, however, the absolute value of the negative peak is smaller than the positive of the peak. The main reason was the non-uniform output power dependence of the microwave generator, which decreased for higher frequencies. When the microwave generator was upgraded, the typical shape of the sweep curve for DNP with P_b centers was observed (Fig. S14, Fig. S15 N 1LO and N -Au samples). Nevertheless, most of the data was obtained with the old microwave generator, and, therefore, the positive peak was selected to study buildup, in agreement with our data at 3.4 T. Almost complete absence of the negative peak for N++ PSi NPs can at least partially be attributed to its generally low polarization combined with the decrease of MW power.

Similar to Fig. 6, for 3.35 T (1.6 K), the extrema of the DNP profiles with MW modulation correspond to the $m_I = \pm 1/2$ hyperfine lines of the simulated EPR spectra (Fig. S16.

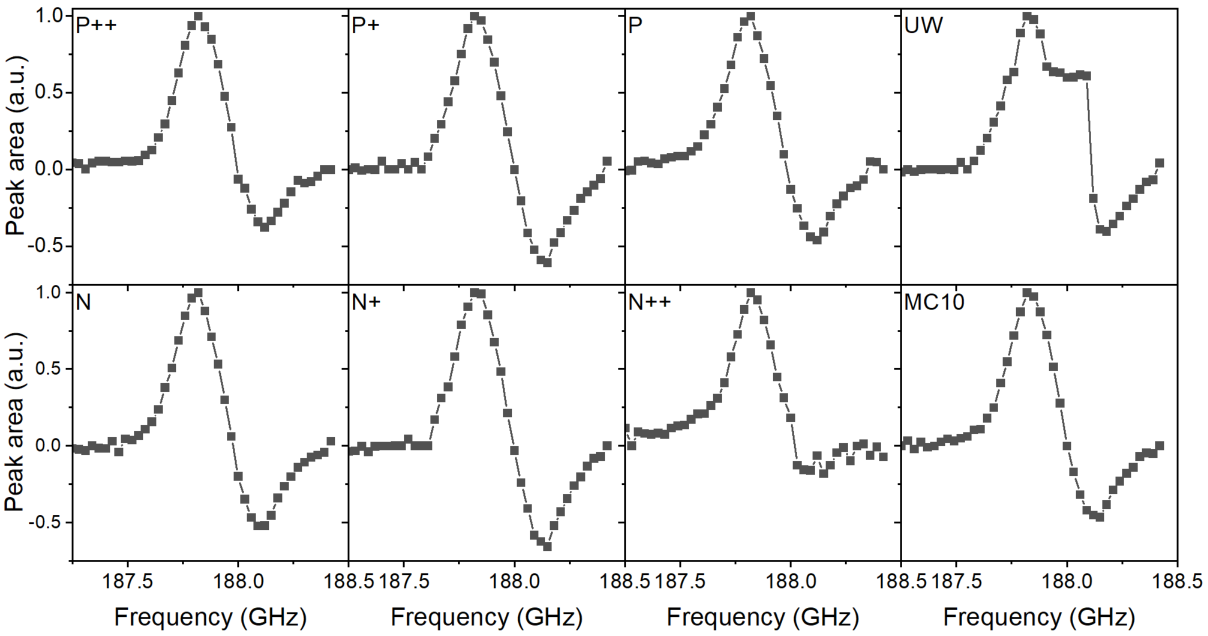

Figure S13: Microwave sweep spectra for thermally oxidized samples of different doping types at 6.7 T and 1.4 K. Each point of a spectrum includes microwave modulation with a frequency of 3 kHz and bandwidth of 150 MHz[34, 36]. Significant decrease of amplitude of the negative peak can be due to decrease of the microwave power with the increase of frequency.

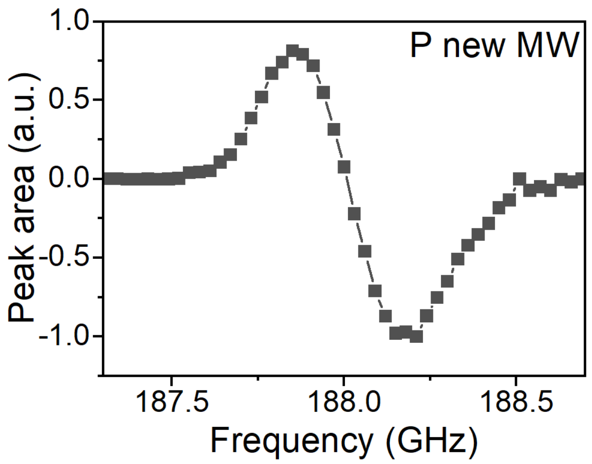

Figure S14: Microwave sweep spectrum of P PSi NPs at 6.7 T and 1.4 K after replacement of microwave generator. The spectrum shows typical asymmetry for DNP of Si using P_b centers[34, 36, 37].

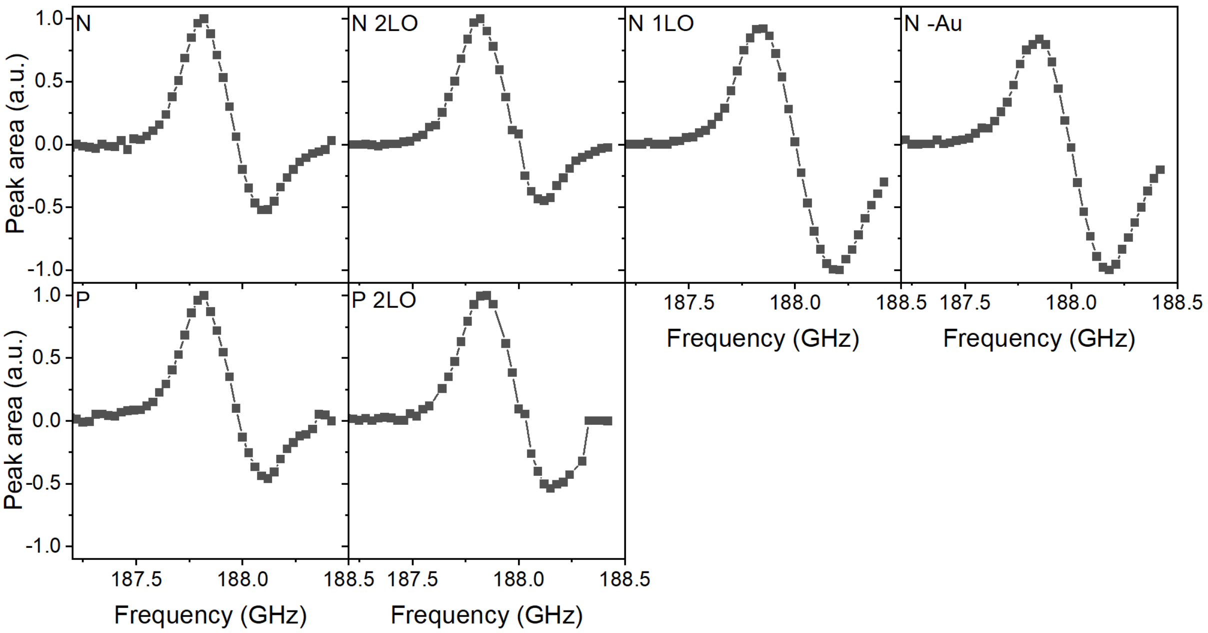

Figure S15: Microwave sweep spectra for differently oxidized N and P PSi NPs at 6.7 T and 1.4 K. Each point of a spectrum includes microwave modulation with a frequency of 3 kHz and bandwidth of 300 MHz[34, 36].

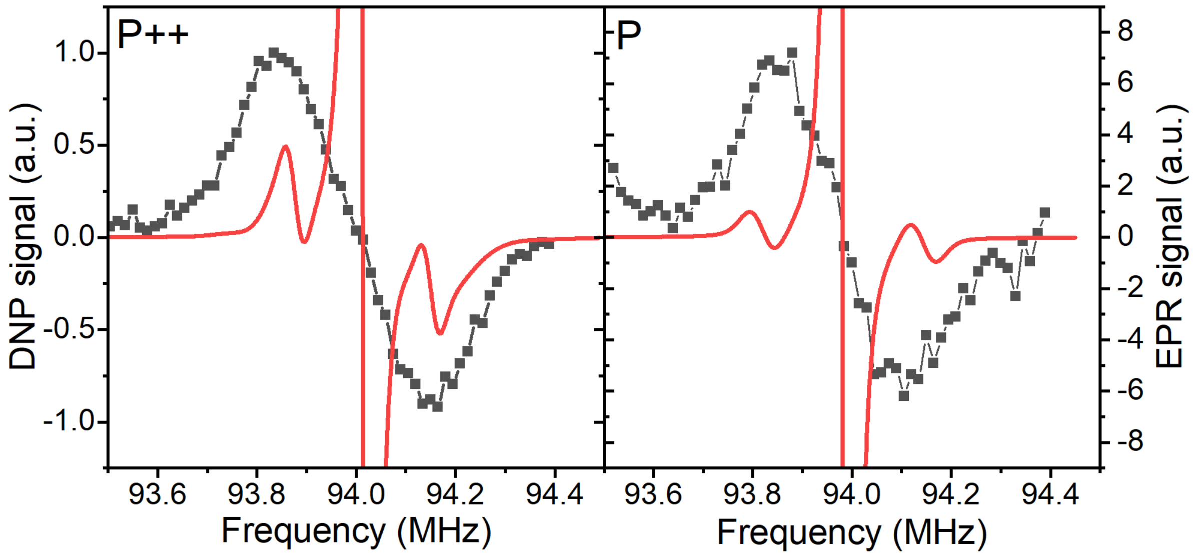

Figure S16: The sweep spectra overlapped with the simulated EPR spectra for P++ and P PSi NPs at 3.35 T and 1.6 K. The sweep was recorded with 100 MHz frequency modulation, 1 kHz sweep rate and 80 mW power.

S3.3 Dynamic nuclear polarization buildup and decay data

Fig. S17 compares different pulse delays (1 and 30 min) and demonstrates that the perturbations be the monitoring RF pulses can be accurately corrected for [38]. Figs. S18 and S19 compare the buildups for the different samples at 3.34 T (3.4 K) and 7 T (3.4 K), respectively. The effects of different oxidation on the build-ups are depicted in Figs. S20 and S21.

Since most of the experiments presented in the current work were recorded with the full MW power available at a given setup, it needs to be investigated if this has a strong influence on the DNP performance. At 7 T (3.4 K), reducing the MW power by a factor of ten (20 instead of 200 mW output power) leads to a minor increase of the steady-state polarization (enhancement) at the expense of a longer buildup time (Fig. S22). This is consistent with the discussion of relaxation enhancement by MW irradiation[39] in Sec. S3.4.

Figs. S19 and S24 compare the hyperpolarization decays at 7 T (3.4 K) and 6.7 T (1.4 K) with a summary of the fitted decay times given in Tab. S3. The decay data is later used to calculated the decay rate constants for the rate-equation model (Secion S3.4). The temperature decrease from 3.4 K to 1.4 K causes the increase in thermal electron polarization (88 % and 99.7 %, respectively) and leads to a drastic reduction in paramagnetic relaxation of nuclei. Relaxation is reduced since virtually all electrons are polarized such that triple spin flips can no longer relax the nuclear polarization[40].

Fig. S25 compares the room temperature decays (at 7 T) of the three most promising samples (P, UW, N). The samples were polarized for around 20 h at 3.34 K (3.4 K) and transferred to the nearby 7 T magnet. Since both magnets were unshielded, we avoided using a strong permanent magnet carrier device for the shuttling which might result in different relaxation behaviors during the transfer for different samples. Furthermore, as we learned later, the 7 T set-up at the time of these measurements had problems with a poor electrical contact, eventually causing increased noise floors in certain measurements.

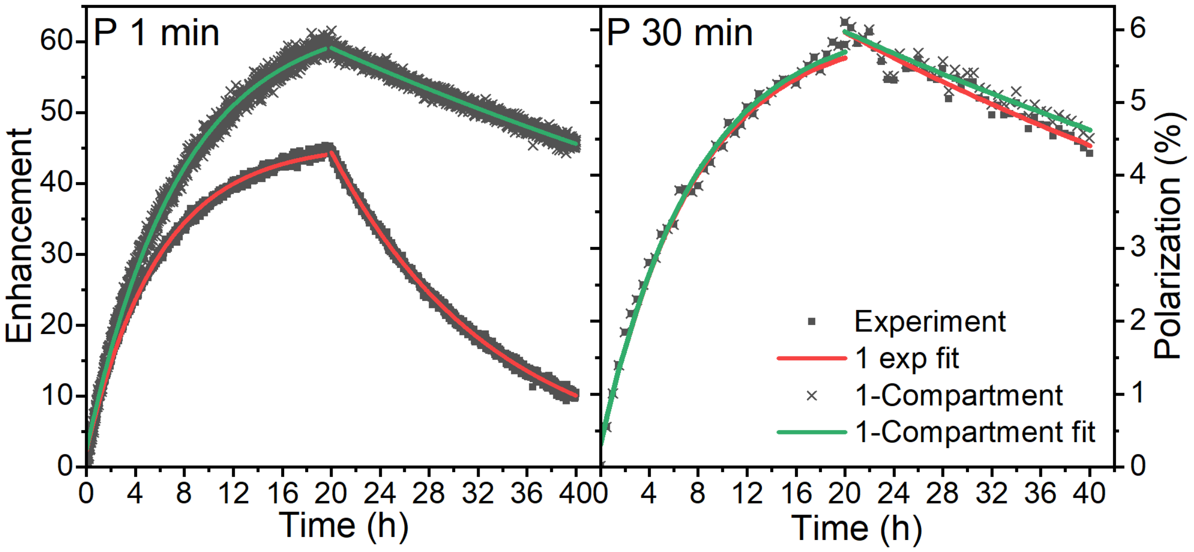

Figure S17: Evaluation of the RF pulse correction and one-compartment model using different sampling rate for the P PSi NPs sample: NMR measurement each 1 min (left) and each 30 min (right) with flip angle $\sim 3°$. The RF pulse correction accurately predicts the polarization buildup and decay for the high sampling rate compared to the low sampling rate provided that NMR flip angle was correctly estimated.

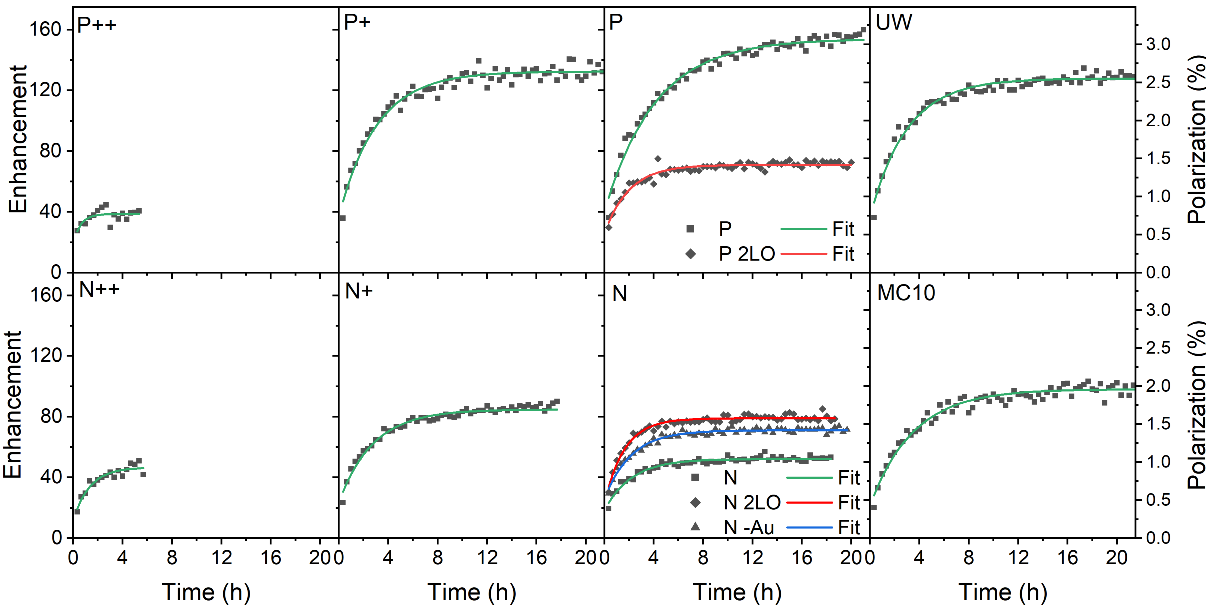

Figure S18: Dynamic nuclear polarization of thermally oxidized PSi NPs with different dopants (dark squares) and single exponential fit with RF pulse correction according to the one-compartment model[38] (green lines). Magnetic field is 3.34 T, temperature is 3.4 K, microwave frequency is 93.83 GHz with around 200 MHz modulation, microwave power is 200 mW.

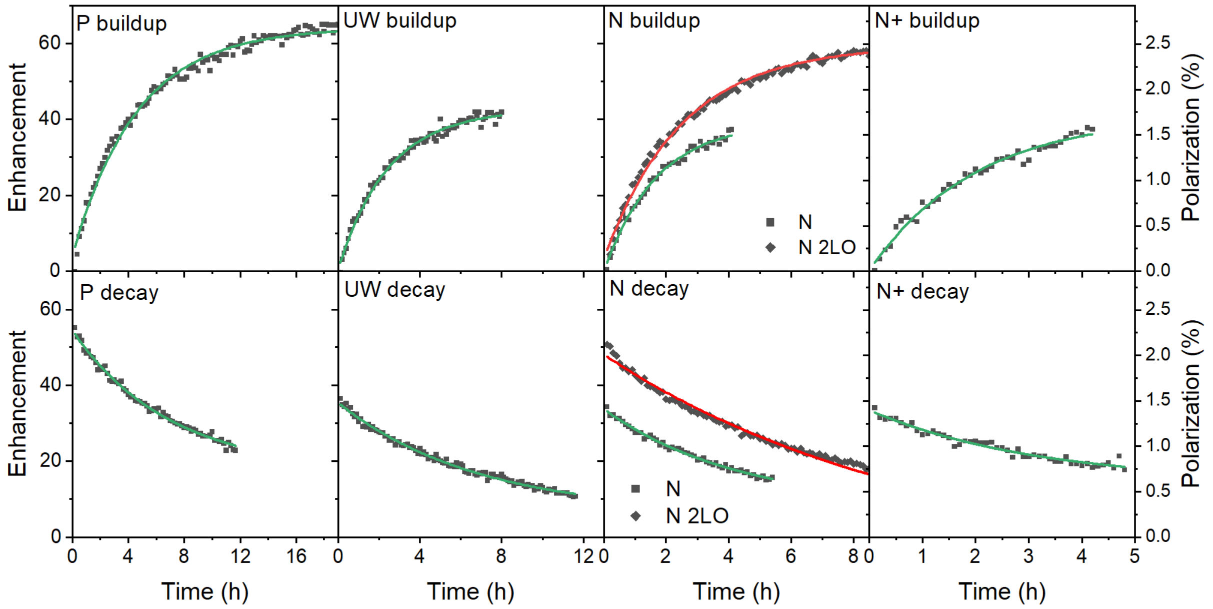

Figure S19: Dynamic nuclear polarization buildup and polarization decay of thermally oxidized PSi NPs with different dopants (dark squares) and single exponential fit with RF pulse correction according to the one-compartment model[38] (green lines). Magnetic field is 7 T, temperature is 3.4 K. The buildup microwave frequency is 197.025 GHz with 300 MHz modulation, microwave power is 200 mW.

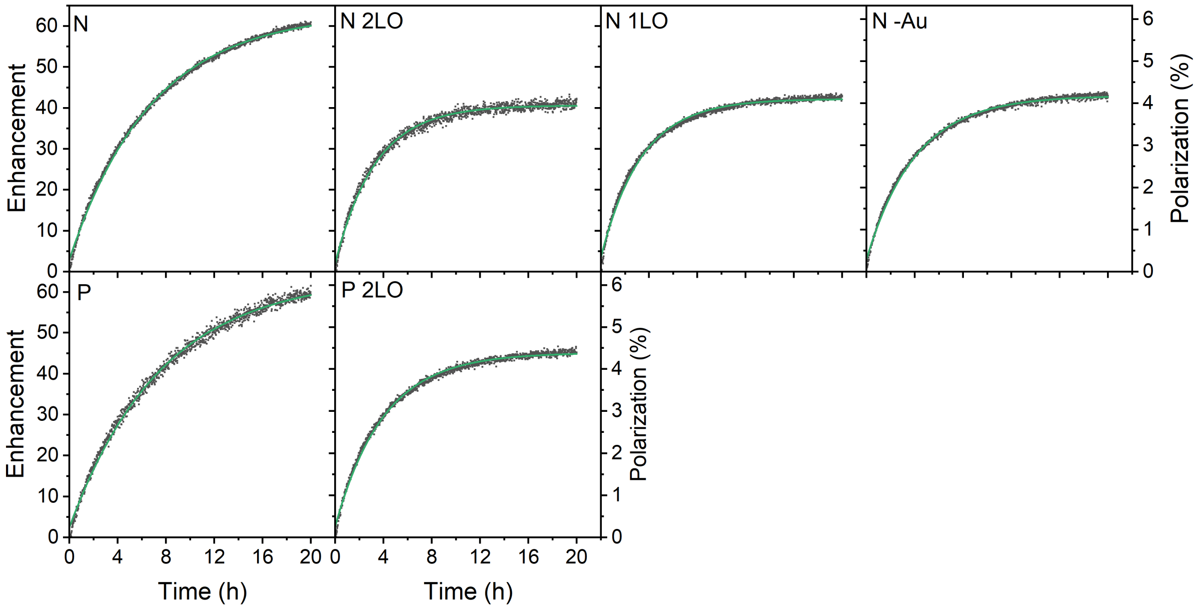

Figure S20: Dynamic nuclear polarization of differently oxidized N and P PSi NPs (dark squares) and single exponential fit with RF pulse correction according to the one-compartment model[38] (green lines). Magnetic field is 6.7 T, temperature is 1.4 K, microwave frequency is 187.82 GHz with 200 MHz modulation, microwave power is 30 mW.

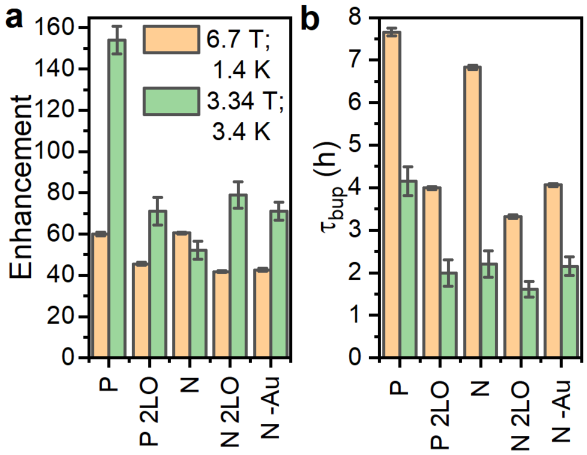

Figure S21: Oxidation induced change of the enhancement (a) and buildup time (b) for the P and N samples at 6.7 T (1.4 K) (orange bars) and 3.34 T (3.4 K) (green bars). The 2LO demotes the two-step liquid oxidation applied after the thermal oxidation either to P or to N sample (Section S1.1). The N -Au sample is the N sample with dissolved Au NPs after LL-MACE, for which the dissolution medium performed the surface oxidation (no thermal oxidation applied).

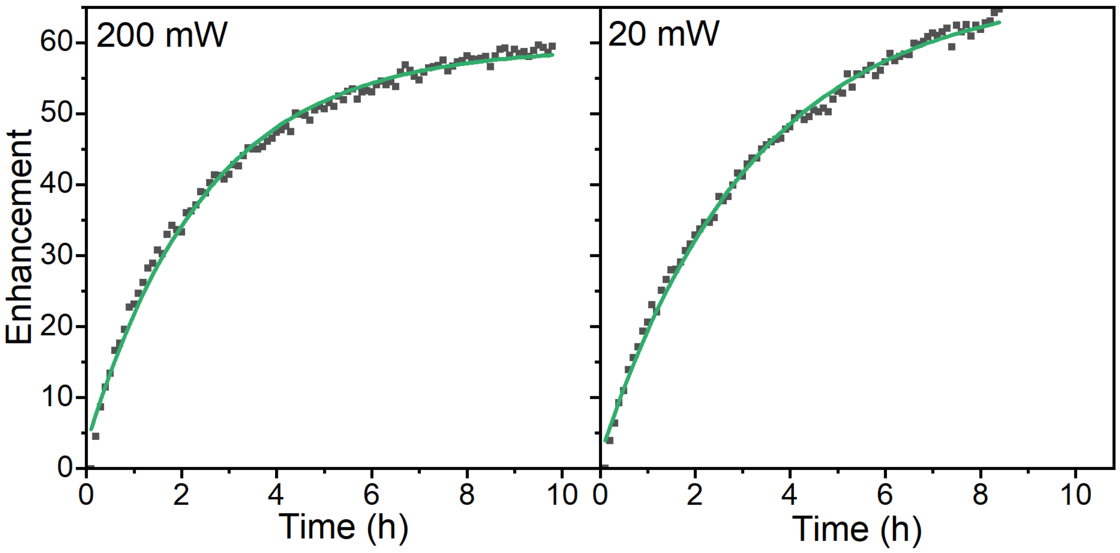

Figure S22: Microwave power dependence of the ^{29}Si polarization buildup at 7 T (3.4 K). The microwave powers were 200 mW (left) and 20 mW (right). The N 2LO sample was used. The DNP buildup times calculated using the one-compartment model were 2.6 ± 0.1 h and 3.4 ± 0.1 h for the 200 mW and 20 mW power, respectively. $k_W = 10.7 \cdot 10^{-3}\,h^{-1}$ and $8.9 \cdot 10^{-3}\,h^{-1}$; $k_R^{bup} = 0.44\,h^{-1}$ and $0.36\,h^{-1}$ for 200 mW and 20 mW, respectively.

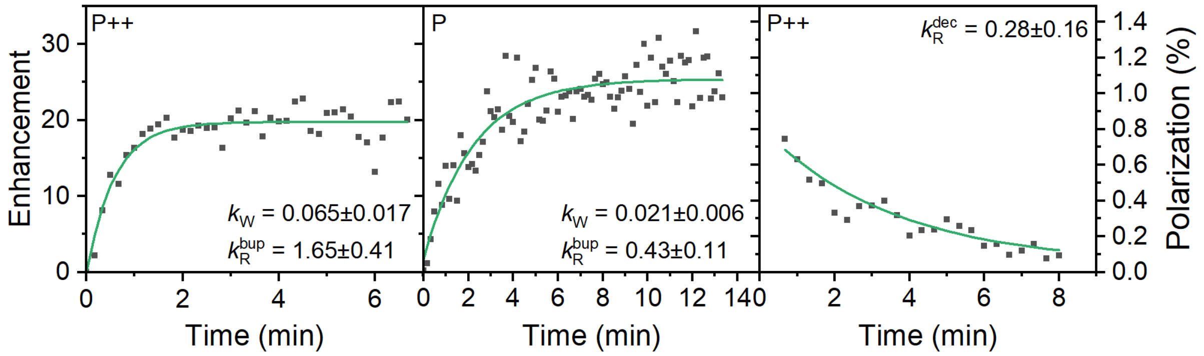

Figure S23: The polarization buildups for P++ and P PSi NPs and the polarization for P++ sample. The buildup was performed with 100 MHz frequency modulation, 1 kHz sweep rate and 80 mW power. The calculated rate constants from the one-compartment model are depicted in the graphs.

Table S3: Relaxation time of the selected PSi NPs at various DNP conditions and switched off microwave radiation.

Abbreviation	τ_{dec}, h	
	6.7 T (1.4 K)	7 T (3.4 K)
P++	17.5 ± 0.8	-
P+	21.8 ± 0.8	-
P	76.9 ± 17.9	7.4 ± 0.2
P 2LO	13.6 ± 3.3	-
UW	20.7 ± 2.4	6.8 ± 0.1
N	79.5 ± 48.2	4.2 ± 0.2
N 2LO	12.3 ± 0.4	-
N -Au	-	3.7 ± 0.4
N+	32.9 ± 2.6	-
N++	20.0 ± 7.5	-
MC10	24.8 ± 3.1	-

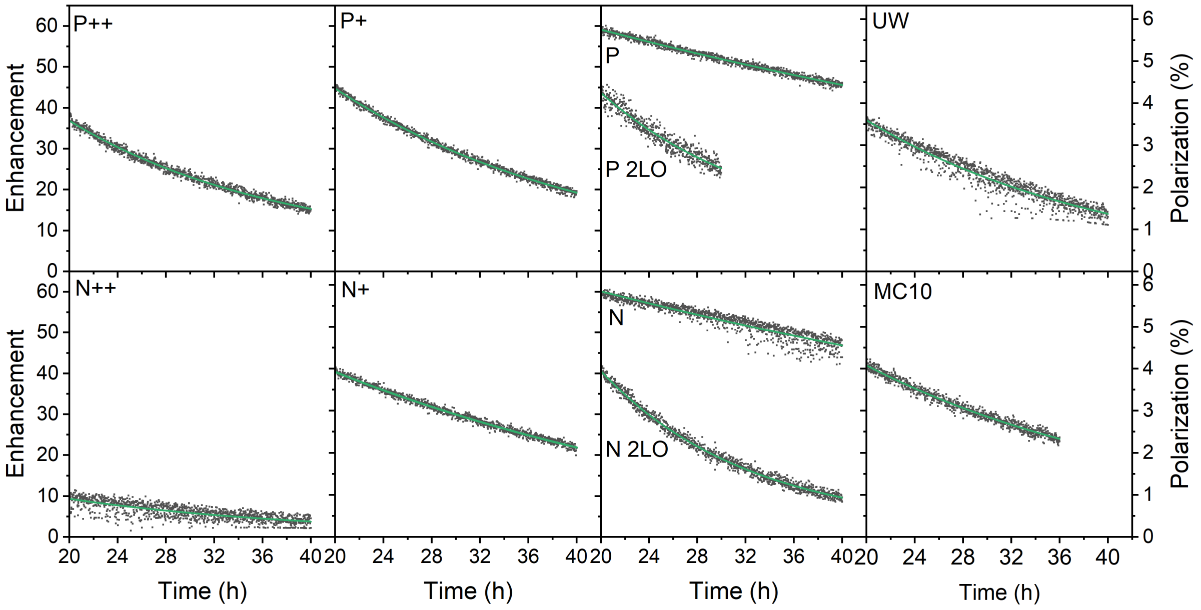

Figure S24: Relaxation of the ^{29}Si polarization for different PSi NPs (dark squares) at 6.7 T and 1.4 K. Single exponential fits (green lines) with RF pulse correction are according to the one-compartment model.

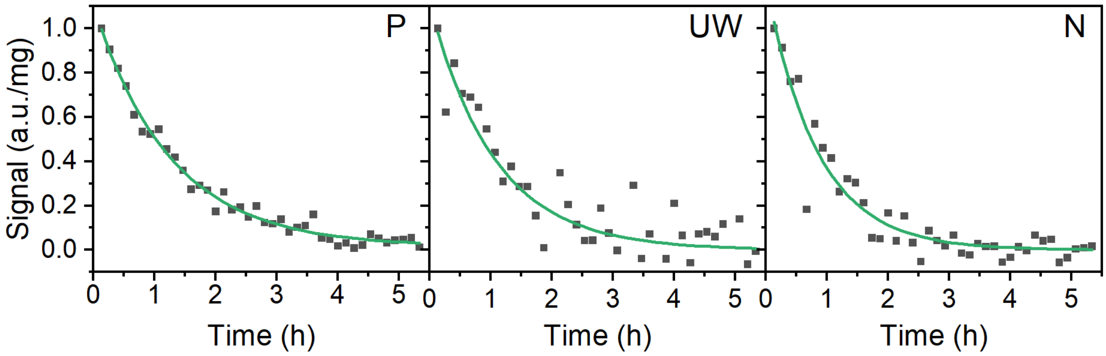

Figure S25: Relaxation of the ^{29}Si polarization for different PSi NPs (dark squares) at 7 T and room temperature (300 K). The decay times are listed in Table 3 in the main text.

S3.4 Quantitative analysis of DNP injection and relaxation

The complex interplay between experimental conditions and structural properties of PSi NPs makes it hard to identify the important parameters that affect the hyperpolarization enhancement and buildup time. On the macroscopic level, it is possible to disentangle enhancements and buildup times using a rate-equation model provided the DNP process can be described with a single time constant[38]. Applying the model, for example, the limited benefit of lower temperatures (1.4 K instead of 3.4 K) can be traced back to a relaxation enhancement by MW irradiation[39].

In the rate-equation model the buildup time τ_{bup} consists of the two competing processes: the nuclear polarization injection rate constant, k_{W}, and the nuclear relaxation rate constant $k_{\mathrm{R}}^{\mathrm{bup}}$. Together with the thermal electron polarization $P_{0\mathrm{e}}$, we can describe the buildup as[38]:

$$\frac{\mathrm{d}P_{\mathrm{n}}(t)}{\mathrm{d}t} = (P_{0\mathrm{e}} - P_{\mathrm{n}})k_{\mathrm{W}} - k_{\mathrm{R}}^{\mathrm{bup}} P_{\mathrm{n}}, \tag{S1a}$$

$$\tau_{\mathrm{bup}}^{-1} = k_{\mathrm{W}} + k_{\mathrm{R}}^{\mathrm{bup}}, \tag{S1b}$$

$$P_{1\mathrm{n}} = P_{0\mathrm{e}} \frac{k_{\mathrm{W}}}{k_{\mathrm{W}} + k_{\mathrm{R}}^{\mathrm{bup}}}\ , \tag{S1c}$$

where $P_{1\mathrm{n}}$ is the steady-state nuclear polarization reached by the end of the DNP process. Eqs. S1b,S1c can be rewritten to[38] $k_{\mathrm{W}} = \tau_{\mathrm{bup}}^{-1} P_{1\mathrm{n}}/P_{0\mathrm{e}}$ and $k_{\mathrm{R}}^{\mathrm{bup}} = \tau_{\mathrm{bup}}^{-1}(1 - P_{1\mathrm{n}}/P_{0\mathrm{e}})$. Since the steady-state nuclear polarizations are low with respect to the electron thermal polarization ($P_{1\mathrm{n}} \ll P_{0\mathrm{e}}$), we can simplify Eqs. S1 to

$$\tau_{\mathrm{bup}}^{-1} \overset{P_{1\mathrm{n}} \ll P_{0\mathrm{e}}}{\approx} k_{\mathrm{R}}^{\mathrm{bup}}, \tag{S2a}$$

$$P_{1\mathrm{n}} = P_{0\mathrm{e}} k_{\mathrm{W}} \tau_{\mathrm{bup}} \overset{P_{1\mathrm{n}} \ll P_{0\mathrm{e}}}{\approx} P_{0\mathrm{e}} \frac{k_{\mathrm{W}}}{k_{\mathrm{R}}^{\mathrm{bup}}}. \tag{S2b}$$

For the decays, the model is much simpler than for the buildups since due to the absence of a polarization injection, *i.e.* the decay time and relaxation rate constant during decay are thus simply related as $k_{\mathrm{R}}^{\mathrm{dec}} = \tau_{\mathrm{dec}}^{-1}$. The decay rate constant $k_{\mathrm{R}}^{\mathrm{dec}}$ is then directly obtained from the single exponential fit of the polarization decay data (Fig. S24, Suppl. Inf.). For the buildup, rate constants are calculated from the maximum thermal electron polarization $P_{0\mathrm{e}}$ at the DNP conditions using the fitted values of τ_{bup} and steady-state DNP polarization $P_{0\mathrm{n}}$. In the following, we discuss the effects of experimental conditions and sample doping along the calculated rate constants.

We start by comparing the influence of experimental conditions on the buildup rate constants (k_{W} and $k_{\mathrm{R}}^{\mathrm{bup}}$) for a limited set of samples with high enhancements (Fig. S26a and S23, Suppl. Inf.). We find the highest injection rates k_{W} to be at 3.4 K and 3.34 T except for the N sample, which has the highest k_{W} at 7 T. For $B_0 \approx 7$ T, k_{W} are similar at 1.4 K and 3.4 K despite different MW powers available (see Methods). The single experiment with 20 mW microwave power at 7 T (3.4 K) gives similar results compared to the full MW power of 200 mW (Fig. S22, Suppl. Inf.). Contrary to $B_0 \approx 7$ T, decreasing the temperature at 3.35 T from 3.4 to 1.4 K severely reduces the enhancements despite similar k_{W} compared to ~ 7 T. To explain such a large difference in enhancements we turn from k_{W} to $k_{\mathrm{R}}^{\mathrm{bup}}$.

The relaxation rate constants during buildup $k_{\mathrm{R}}^{\mathrm{bup}}$ significantly differ between the experimental conditions and strongly affect the achieved enhancements and nuclear polarizations. $k_{\mathrm{R}}^{\mathrm{bup}}$ rates are typically several times lower at 1.4 K (6.7 T) than at 3.4 K (Fig. S26a) consistent with the decrease of electron spin lattice relaxation time[41]. Contrary to 6.7 T, at 3.35 T and 1.4 K $k_{\mathrm{R}}^{\mathrm{bup}}$ is the highest, which detrimentally affects the enhancements (Fig. S23, Suppl. Inf.). At 3.4 K, $k_{\mathrm{R}}^{\mathrm{bup}}$ is similar at 3.34 and 7 T.

The relaxation rate constants during the decay $k_{\mathrm{R}}^{\mathrm{dec}}$ conformed to the following trend: slowest at 1.4 K (6.7 T) (Fig. S26b), intermediate at 7 T (3.4 K) and fastest at 3.34 T (3.4 K) (Figs. S28, S27 and Tbl. S3). This trend is consistent with the gradual increase of electron spin polarization from ~60 % at 3.34 T, 3.4 K up to ~99.7 % at 6.7 T, 1.4 K. The similar relaxation reduction at temperatures below ~2 K at ~7 T has been observed in our setups for ^{1}H glassy matrices[39] as well as for diamonds before (data not shown). These results are attributed to high electron polarization which strongly reduces thermal paramagnetic relaxation approximated by $1 - P_{0\mathrm{e}}^2$[42].

The discrepancy between relaxation rate constants during buildup ($k_{\mathrm{R}}^{\mathrm{bup}}$) and decay ($k_{\mathrm{R}}^{\mathrm{dec}}$) is the most pronounced at 1.4 K. During the buildup, relaxation rates are five to ten times faster at 6.7 T (Fig. S26b)

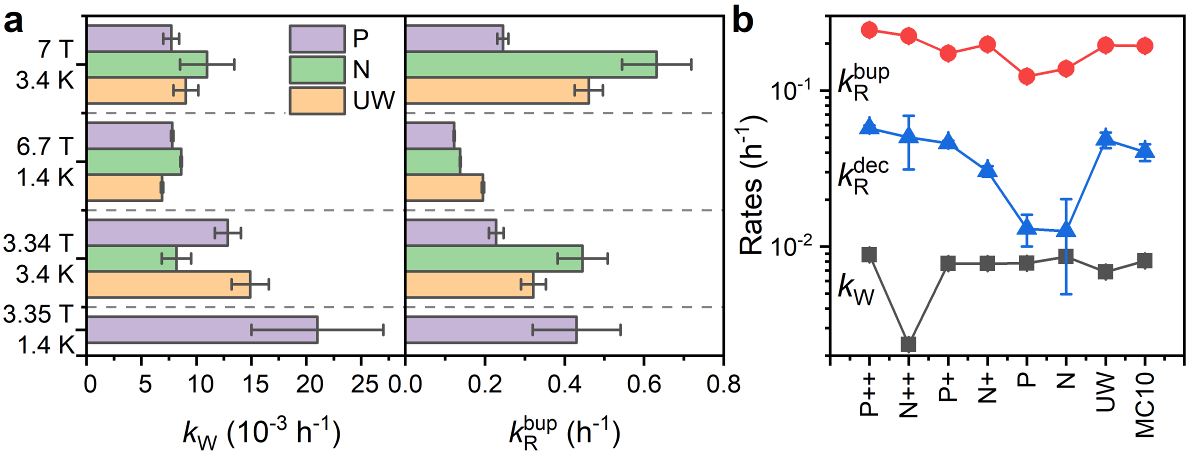

Figure S26: One-compartment model parameters calculated from the mono-exponential fitting of the experimental hyperpolarization buildups and decays. (a) Comparison of k_W and k_R^{bup} between three experimental conditions for the selected P, UW and N PSi NPs. Note the 10^{-3} scale for k_W. (b) Polarization buildup (dark squares and red circles) and decay (blue triangles) rates for the PSi NPs with different doping and oxidation. The data was acquired at 6.7 T, 1.4 K. Lines are a guide for the eye.

and more than an order of magnitude faster at 3.35 T (Fig. S23, Suppl. Inf.) compared to the MW-off decay. At 3.4 K and 7 T the k_R^{dec}/k_R^{bup} ratio is in the range of 2.5 ± 0.5 while at 3.34 T the ratio is close to 1 (Sec. S3.3, Suppl. Inf.). The observed strong increase of the k_R^{bup} relaxation rate either with the decrease of temperature at 3.35 T or with the decrease of magnetic field at 1.4 K is consistent with the results found in Ref. [39]. There, the relaxation enhancement during buildup is ascribed to the increase of the triple-spin flip rate induced by MW irradiation. At lower temperatures, on the one hand, paramagnetic relaxation of nuclei is reduced as a result of higher electron polarization ($1 - P_{0e}^2$). On the other hand, longer electronic relaxation times increase the saturation of the EPR line causing an increased relaxation by MW irradiation. With the increase of magnetic fields, the anisotropic EPR line is broadened, which reduces its spectral density and, consequently, the number of electron spin pairs that could cause an efficient three-spin nuclear paramagnetic relaxation. Thus, the higher relaxation enhancement at lower temperatures and lower fields increases k_R^{bup} and results in the lowest enhancements observed at 3.35 T (1.4 K).

Converting the analyzed rate constants back to the measured DNP parameters, it is now possible to trace the influence of experimental conditions on the buildup time τ_{bup} and enhancements (or nuclear polarization P_{1n}). The injection rates k_W vary relatively weakly between the utilized temperatures, magnetic fields and MW powers. In contrast, the relaxation rates k_R^{bup} are strongly affected by the experimental conditions which in turn strongly influence τ_{bup} and P_{1n}. The large k_R^{bup} prevent excessively long buildup at the expense of rather low achievable polarization levels (Eq. S1c). Conversely, the lowest observed k_R^{bup} and k_R^{dec} at 6.7 T (1.4 K) result in the highest polarization and longest buildup time. At 3.4 K, the relatively field independent k_W and k_R^{bup} result in similar steady-state polarizations and buildup times. However, the higher thermal nuclear polarization at 7 T means that the enhancements are approximately halved for the same gained nuclear polarization. Cooling to 1.4 K at 3.35 T results in a very large relaxation rate during the buildup reflected by a fast buildup time and low steady-state polarization. The field independent injection rate facilitates further study since typical DNP models for the electron-nuclear HF-mediated polarization transfer predict the decrease of triple spin flip transition rate with increasing magnetic field strength[40, 43].

Next, we compare the rate equation parameters at 6.7 T (1.4 K) measurements across the different samples (Fig. S26b). The DNP injection k_W is nearly identical for all samples except for the N++. The relaxation during the buildup (k_R^{bup}) shows a weak dependence on the doping level with lower doping levels having lower relaxation rate constants. The UW sample has higher relaxation and lower injection compared to P and N samples, resulting in its comparatively lower enhancement. This is even more pronounced for the relaxation during decay (k_R^{dec}) for which the UW sample has a nearly and order of magnitude faster relaxation than P and N with the latter two samples standing out among all samples with their smallest

k_R^{dec}. For all the samples, k_R^{bup}/k_R^{dec} is between 5 and 10, suggesting a strong relaxation enhancement by MW irradiation[39]. In contrast, increasing the temperature to 3.4 K (7 T) which corresponds to a reduction in thermal electron polarization from around 99.7 to 88%, reduces the relaxation enhancement to around two- or three-fold (Fig. S27b). Reducing the field to 3.34 K at 3.4 K (around 59% thermal electron polarization) leads to the absence of a relaxation enhancement (Fig. S28).

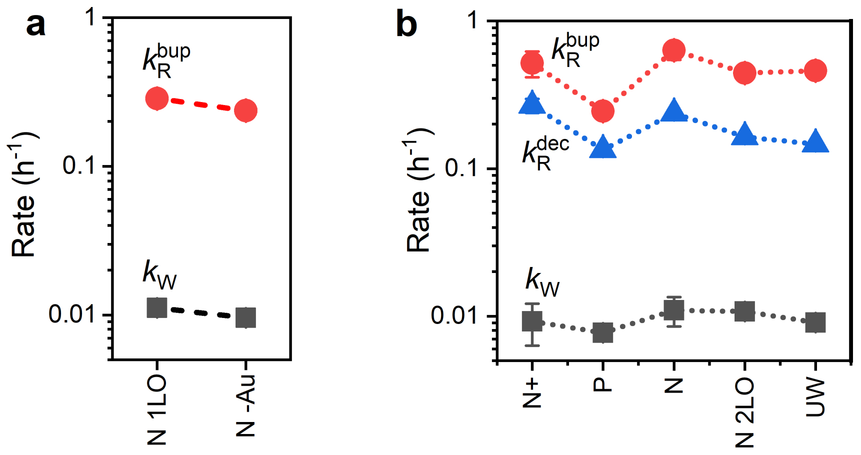

Figure S27: The polarization injection rates k_W and the decay rates during the buildup k_R^{bup} and decay k_R^{dec}. (a) Rates for the N 1LO and N -Au samples at 6.7 T (1.4 K). (b) Rates for the selected samples at 7 T (3.4 K). The rates were extracted from the one-compartment model[38]. Lines are guide to the eye.

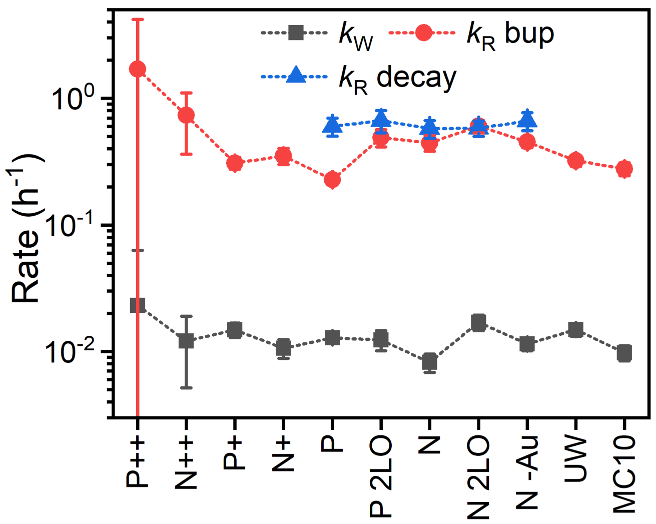

Figure S28: One-compartment model parameters calculated from the polarization build (dark squares and red circles) for the PSi NPs with different doping and oxidation. Decays were recorded only for around 5 hours only, resulting in underestimated decay relaxation rates. Magnetic field strength is 3.34 T, temperature is 3.4 K. Lines are guide to the eye.

At 6.7 T (1.4 K), the additional liquid oxidation and the oxidation induced by the Au removal increased the relaxation rates for the P and N samples (Fig. 4) with little difference between the two oxidation methods at 6.7 T (1.4 K) for the N sample (Fig. S27a). This increased relaxation turned into lower enhancements and faster buildup times compared to the thermally oxidized PSi NPs of the same doping density. The influence of additional liquid oxidation remains unclear provided that these oxidations yield little effect on the structure and number of the P_b centers (Fig. 1 and Sec. S2.2, Suppl. Inf.). In contrast, at 3.34 T (3.4 K), the additional liquid oxidation and oxidation from the gold removal increased the achievable enhancements for N samples without causing a prolonged buildup. We note that the removal of the

gold nanoparticle catalyst has little effect on the DNP suggesting that the nuclear polarization inside the particle is well protected from surface modifications including metallic NPs.

To quantify the analysis of all samples at 6.7 T (1.4 K), we performed a correlation analysis (see Methods) between different sample properties and the experimentally measured DNP parameters (buildup time, polarization and rate constants). Below, we summarize the most significant correlations found:

- The specific surface area and, correspondingly, the density of P_b centers is controlled by the doping density through its known influence on the outcome of LL-MACE (correlation coefficient equals to 0.9)[5, 6]. However, the role of doping is more complicated because high doping would lead to a large number of quenched nuclear spins which Larmor frequencies that are shifted by HF interaction with the spatially extended wave functions of shallow dopants (phosphorous and boron). This might be the reason of exceptionally small k_{W} in the N++ sample. With the increase of temperature, the presence of delocalized thermally excited charge carriers would lead to fast room temperature relaxation for high doping density[44]. On the other hand, depletion space-charge layers formed due to Fermi level pinning by charged P_b centers may almost completely deplete the pore walls from the charge carriers for the moderate and low doping densities[6] *i.e.*, for all the PSi NPs except N++ and P++. Indeed, no correlation is found between the doping densities and the enhancements or the rate-equation model rates.
- k_{W} shows a correlation of 0.59 and -0.32 with the steady-state polarization in P_0 and the buildup time τ_{bup}. For $k_{\mathrm{R}}^{\mathrm{bup}}$ these correlations are -0.48 and 0.97. Together, this is in good agreement with the expectation of Eq. S2 in which τ_{bup} is inversely proportional to $k_{\mathrm{R}}^{\mathrm{bup}}$ while P_0 depends on the ratio $k_{\mathrm{W}}/k_{\mathrm{R}}^{\mathrm{bup}}$.
- The injection rate k_{W} seems to depend mainly on the density of P_b centers with a positive correlation coefficient of 0.74.
- The $k_{\mathrm{R}}^{\mathrm{bup}}$ and $k_{\mathrm{R}}^{\mathrm{dec}}$ relaxation rates show almost no correlation with the density of P_b centers (absolute value below 0.1) and only weak correlation with the ΔB_{pp}^{L} (absolute values up to 0.18).
- All the rates depend moderately on the values of HF constant A_{iso} and its strain as well on the Lorenzian line width of the $P_b^{(111)}$ centers.

These results might suggest that the DNP injection k_{W} and relaxation $k_{\mathrm{R}}^{\mathrm{bup}}$ can be controlled separately in the NP synthesis as k_{W} is sensitive to the overall P_b density while $k_{\mathrm{R}}^{\mathrm{bup}}$ and $k_{\mathrm{R}}^{\mathrm{dec}}$ depend on the local properties of the P_b centers.

Overall, DNP injection appears rather uniform across the samples while the relaxation rates during the buildup govern the buildup time and polarization enhancement. Furthermore, the DNP injection varies much less with experimental conditions than the relaxation rate. However, suppressing the relaxation with lower temperatures (1.4 K instead of 3.4 K) shows at best only a modest improvement as relaxation enhancement by MW irradiation[39] becomes more pronounced. Combining lower temperatures with higher fields partially suppresses the relaxation enhancement by MW irradiation[39] and results in the highest nuclear polarizations at 6.7 T and 1.4 K.

S4 Density functional theory (DFT) simulations

Spin polarized density functional theory (DFT) simulations to calculate the HF and SHF interaction from first principles were performed with the *CP-PAW* code (http://www2.pt.tu-clausthal.de/paw/), employing the projector augmented wave (PAW) approach[45] and Perdew-Burke-Ernzerhof (PBE) exchange functional[46]. The plane-wave cutoffs were set to 40 Ry for the wave functions and to 80 Ry for the charge density. The silicon lattice constant was set to 0.5431 nm. The simulation box consisted of five conventional eight-atomic unit cells in each spatial direction, resulting in 1000 lattice sites. A single silicon atom in the centre of unit cell was replaced by either a boron or a phosphorous atom. The isotropic Fermi-contact HF interaction of the P dopant was calculated to 91.1 MHz - in agreement with the experimental value of 117.5 MHz[47] considering the finite unit cell of the simulation and PBE functional[48]. For the B dopant, the calculated isotropic Fermi-contact HF interaction is 1.4 MHz for an applied strain of 4 kbar as employed in previous DNP experiments[49]. The largest computed Si SHF

for the P dopant is 7.3 MHz, which is close to the measured 6 MHz[47]. The values for the P dopant are more than an order of magnitude larger than the computed 0.5 MHz in the B case.

References

[1] Joakim Riikonen, Mikko Salomäki, Jessica Van Wonderen, Marianna Kemell, Wujun Xu, Ossi Korhonen, Mikko Ritala, Fraser MacMillan, Jarno Salonen, and Vesa Pekka Lehto. Surface chemistry, reactivity, and pore structure of porous silicon oxidized by various methods. *Langmuir*, 28(28): 10573–10583, 2012. ISSN 07437463. doi: 10.1021/la301642w.

[2] Konstantin Tamarov, Julie Tzu Wen Wang, Juuso Kari, Emilia Happonen, Ilkka Vesavaara, Matti Niemelä, Paavo Perämäki, Khuloud T. Al-Jamal, Wujun Xu, and Vesa Pekka Lehto. Comparison between Fluorescence Imaging and Elemental Analysis to Determine Biodistribution of Inorganic Nanoparticles with Strong Light Absorption. *ACS Applied Materials and Interfaces*, 13(34):40392–40400, 2021. ISSN 19448252. doi: 10.1021/acsami.1c11875.

[3] Tuomo Nissinen, Simo Näkki, Hanne Laakso, Dalius Kučiauskas, Algirdas Kaupinis, Mikko I. Kettunen, Timo Liimatainen, Mervi Hyvönen, Mindaugas Valius, Olli Gröhn, and Vesa Pekka Lehto. Tailored Dual PEGylation of Inorganic Porous Nanocarriers for Extremely Long Blood Circulation in Vivo. *ACS Applied Materials & Interfaces*, 8(48):32723–32731, dec 2016. ISSN 19448252. doi: 10.1021/acsami.6b12481.

[4] Wujun Xu, Joakim Riikonen, Tuomo Nissinen, Mika Suvanto, Kirsi Rilla, Bojie Li, Qiang Wang, Feng Deng, and Vesa Pekka Lehto. Amine surface modifications and fluorescent labeling of thermally stabilized mesoporous silicon nanoparticles. *Journal of Physical Chemistry C*, 116(42):22307–22314, oct 2012. ISSN 19327447. doi: 10.1021/JP303199S/SUPPL_FILE/JP303199S_SI_001.PDF. URL https://pubs.acs.org/doi/abs/10.1021/jp303199s.

[5] K. Tamarov, Joseph D. Swanson, Bret A. Unger, Kurt W. Kolasinski, Alexis T. Ernst, Mark Aindow, Vesa-Pekka Lehto, and Joakim Riikonen. Controlling the nature of etched Si nanostructures: High versus low load metal-assisted catalytic etching (MACE) of Si powders. *ACS Applied Materials & Interfaces*, 12(4):4787–4796, 2020. doi: 10.1021/acsami.9b20514. URL https://doi.org/10.1021/acsami.9b20514.

[6] K. Tamarov, R. Kiviluoto, J.D. Swanson, B.A. Unger, A.T. Ernst, M. Aindow, J. Riikonen, V.-P. Lehto, and K.W. Kolasinski. Low-Load Metal-Assisted Catalytic Etching Produces Scalable Porosity in Si Powders. *ACS Applied Materials & Interfaces*, 12(43):48969–48981, 2020. ISSN 19448252. doi: 10.1021/acsami.0c13980.

[7] E. A. Konstantinova. Characterization of Porous Silicon by EPR and ENDOR. In Leigh T. Canham, editor, *Handbook of Porous Silicon*, pages 627–654. Springer International Publishing, 2nd edition, 2018. ISBN 978-3-319-71379-3.

[8] A. Stesmans and V. V. Afanas'ev. Electron spin resonance features of the P¡sub¿b1¡/sub¿ interface defect in thermal (100)Si/SiO¡sub¿2¡/sub¿. *Materials Science Forum*, 258-263(9993):1713–1718, 1997. ISSN 02555476. doi: 10.4028/www.scientific.net/msf.258-263.1713.

[9] K. L. Brower. Electron paramagnetic resonance studies of Si-SiO2 interface defects. *Semiconductor Science and Technology*, 4(12):970–979, 1989. ISSN 02681242. doi: 10.1088/0268-1242/4/12/002.

[10] Stefan Stoll and Arthur Schweiger. EasySpin, a comprehensive software package for spectral simulation and analysis in EPR. *Journal of Magnetic Resonance*, 178(1):42–55, 2006. ISSN 10907807. doi: 10.1016/j.jmr.2005.08.013.

[11] H. J. Von Bardeleben, M. Schoisswohl, and J. L. Cantin. Electron paramagnetic resonance study of defects in oxidized and nitrided porous Si and Si(1-x)Ge(x). *Colloids and Surfaces A: Physicochemical and Engineering Aspects*, 115:277–289, 1996. ISSN 09277757. doi: 10.1016/0927-7757(96)03604-7.

[12] Tuomo Nissinen, Timo Ikonen, Mejor Lama, Joakim Riikonen, and Vesa-Pekka Lehto. Improved production efficiency of mesoporous silicon nanoparticles by pulsed electrochemical etching. *Powder Technology*, 288:360–365, 2016. ISSN 00325910. doi: 10.1016/j.powtec.2015.11.015. URL http://www.sciencedirect.com/science/article/pii/S0032591015301583.

[13] A. Stesmans and V. V. Afanas'ev. Electron spin resonance features of interface defects in thermal (100) Si/SiO2. *Journal of Applied Physics*, 83(5):2449–2457, 1998. ISSN 00218979. doi: 10.1063/1.367005.

[14] A. Stesmans and G. Van Gorp. Observation of dipolar interactions between Pb0 defects at the (111) Si/SiO2 interface. *Physical Review B*, 42(6):3765–3768, 1990. doi: 10.1103/PhysRevB.42.3765.

[15] Van Gorp and A. Stesmans. Dipolar interaction between [111] Pb defects at the (111)Si/SiO2 interface revealed by electron-spin resonance. *Physical Review B*, 45(8):4344–4371, 1992.

[16] B. K. Meyer, V. Petrova-Koch, T. Muschik, H. Linke, P. Omling, and V. Lehmann. Electron spin resonance investigations of oxidized porous silicon. *Applied Physics Letters*, 63(14):1930–1932, 1993. ISSN 00036951. doi: 10.1063/1.110652. URL `http://dx.doi.org/10.1063/1.110652`.

[17] Aaron Himmler, Mohammed M. Albannay, Gevin von Witte, Sebastian Kozerke, and Matthias Ernst. Electroplated waveguides to enhance DNP and EPR spectra of silicon and diamond particles. *Magnetic Resonance*, 3(2):203–209, 2022. ISSN 26990016. doi: 10.5194/mr-3-203-2022.

[18] C F Young, E H Poindexter, and G J Gerardi. Electron paramagnetic resonance of porous silicon : Observation and identification of conduction-band electrons. *Journal of Applied Physics*, 81:7468–7470, 1997. doi: 10.1063/1.365289.

[19] C. Young, E. Poindexter, and G. Gerardi. Electron paramagnetic resonance of conduction-band electrons in silicon. *Physical Review B*, 55(24):16245–16248, 1997. ISSN 1550235X. doi: 10.1103/PhysRevB.55.16245.

[20] Edward H Poindexter and Philip J Caplan. Characterization of Si/SiO2 interface defects by electron spin resonance. *Progress in Surface Science*, 14(3):201–294, 1983.

[21] E. H. Poindexter, G. J. Gerardi, M. E. Rueckel, P. J. Caplan, N. M. Johnson, and D. K. Biegelsen. Electronic traps and Pb centers at the Si/SiO2 interface: Band-gap energy distribution. *Journal of Applied Physics*, 56(10):2844–2849, 1984. ISSN 00218979. doi: 10.1063/1.333819.

[22] F. C. Rong, J. F. Harvey, E. H. Poindexter, and G. J. Gerardi. Nature of Pb-like dangling-orbital centers in luminescent porous silicon. *Applied Physics Letters*, 63(7):920–922, 1993. doi: 10.1063/1.109845.

[23] F. C. Rong, J. F. Harvey, E. H. Poindexter, and G. J. Gerardi. Identification and Properties of Pb-like Centers in Photoluminescent Porous Silicon. *Microelectronic Engineering*, 22:147–150, 1993. ISSN 00036951. doi: 10.1063/1.109845.

[24] H. J. von Bardeleben, M. Chamarro, A. Grosman, V. Morazzani, C. Ortega, J. Siejka, and S. Rigo. Pb-defects and visible photoluminescence in porous silicon. *Journal of Luminescence*, 57:39–43, 1993. ISSN 00222313. doi: 10.1016/0022-2313(93)90103-T.

[25] Patrick Bertrand. *Electron Paramagnetic Resonance Spectroscopy Fundamentals*. Springer Cham, 2020. ISBN 978-3-030-39662-6.

[26] K. L. Brower and T.J. Headley. Dipolar interactions between dangling bonds at the (111) Si-SiO2 interface. *Physical Review B*, 34(6):3610–3619, 1986.

[27] H. J. Von Bardeleben, D. Stievenard, A. Grosman, C. Ortega, and J. Siejka. Defects in porous p-type Si: An electron-paramagnetic-resonance study. *Physical Review B*, 47(16):10899–10902, 1993. ISSN 01631829. doi: 10.1103/PhysRevB.47.10899. URL `http://dx.doi.org/10.1103/PhysRevB.47.10899`.

[28] R. Laiho, L. S. Vlasenko, M. M. Afanasiev, and M. P. Vlasenko. Electron paramagnetic resonance in heat-treated porous silicon. *Journal of Applied Physics*, 76(7):4290–4293, 1994. ISSN 00218979. doi: 10.1063/1.357313.

[29] M. C. Cassidy, H. R. Chan, B. D. Ross, P. K. Bhattacharya, and C. M. Marcus. In vivo magnetic resonance imaging of hyperpolarized silicon particles. *Nature Nanotechnology*, 8(5):363–368, 2013. ISSN 17483395. doi: 10.1038/nnano.2013.65. URL `http://dx.doi.org/10.1038/nnano.2013.65`.

[30] Serena Iacovo and Andre Stesmans. Inherent interface defects in thermal (211)Si/SiO2:29Si hyperfine interaction. In *AIP Conference Proceedings*, volume 1624, pages 49–57, 2014. ISBN 9780735412613. doi: 10.1063/1.4900456.

[31] Y. Xiao, T. J. McMahon, J. I. Pankove, and Y. S. Tsuo. Existence of a Pb1-like defect center in porous silicon. *Journal of Applied Physics*, 76(3):1759–1763, 1994. ISSN 00218979. doi: 10.1063/1.358433. URL https://dx.doi.org/10.1063/1.358433.

[32] A. Stesmans and V. V. Afanas'ev. Undetectability of the Pb1 point defect as an interface state in thermal (100)Si/SiO2. *Journal of Physics Condensed Matter*, 10(1), 1998. ISSN 09538984. doi: 10.1088/0953-8984/10/1/003.

[33] J. Braet and A. Stesmans. Low T characterization of the Pbo- defect spin relaxation at (111) Si/SiO2 interfaces. *Physica B+C*, 126(1-3):463–464, 1984. doi: 10.1016/0378-4363(84)90206-7.

[34] Grzegorz Kwiatkowski, Yevhen Polyhach, Fabian Jähnig, Toni Shiroka, Fabian H. L. Starsich, Matthias Ernst, and Sebastian Kozerke. Exploiting Endogenous Surface Defects for Dynamic Nuclear Polarization of Silicon Micro- and Nanoparticles. *The Journal of Physical Chemistry C*, 122 (44):25668–25680, oct 2018. ISSN 1932-7447. doi: 10.1021/acs.jpcc.8b08926. URL http://pubs.acs.org/doi/10.1021/acs.jpcc.8b08926.

[35] Martin Stutzmann and David K. Biegelsen. Microscopic nature of coordination defects in amorphous silicon. *Physical Review B*, 40(14):9834–9840, 1989. ISSN 01631829. doi: 10.1103/PhysRevB.40.9834. URL http://dx.doi.org/10.1103/PhysRevB.40.9834.

[36] Grzegorz Kwiatkowski, Fabian Jähnig, Jonas Steinhauser, Patrick Wespi, Matthias Ernst, and Sebastian Kozerke. Nanometer size silicon particles for hyperpolarized MRI. *Scientific Reports*, 7: 7946, 2017. ISSN 20452322. doi: 10.1038/s41598-017-08709-0.

[37] A. E. Dementyev, D. G. Cory, and C. Ramanathan. Dynamic Nuclear Polarization in Silicon Microparticles. *Physical Review Letters*, 100:127601, 2008. doi: 10.1103/PhysRevLett.100.127601. URL http://dx.doi.org/10.1103/PhysRevLett.100.127601.

[38] Gevin von Witte, Matthias Ernst, and Sebastian Kozerke. Modelling and correcting the impact of RF pulses for continuous monitoring of hyperpolarized NMR. *Magnetic Resonance*, (April):1–16, 2023. doi: 10.5194/mr-2023-5.

[39] Gevin Von Witte, Aaron Himmler, Sebastian Kozerke, and Matthias Ernst. Relaxation enhancement by microwave irradiation may limit dynamic nuclear polarization. *Physical Chemistry Chemical Physics*, 26(12):9578–9585, 2024. ISSN 1463-9076, 1463-9084. doi: 10.1039/D3CP06025J. URL https://xlink.rsc.org/?DOI=D3CP06025J.

[40] W. T. Wenckebach. *Essentials of Dynamic Nuclear Polarisation*. Spindrift Publications, 2016. ISBN 9789075541182.

[41] A. Stesmans. Comparison of the low-temperature esr properties of Pb0 defects residing at the interfaces of differently-oxidized Si/SiO2 structures. *Zeitschrift für Physikalische Chemie*, 151:191–209, 1987. ISSN 09429352. doi: 10.1524/zpch.1987.151.Part_1_2.191.

[42] A. Abragam and M. Goldman. *Nuclear Magnetism: Order and Disorder*. Oxford University Press, 1982.

[43] W. Th. Wenckebach. Electron Spin–Spin Interactions in DNP: Thermal Mixing vs. the Cross Effect. *Applied Magnetic Resonance*, 52(7):731–748, July 2021. ISSN 1613-7507. doi: 10.1007/s00723-021-01335-0. URL https://doi.org/10.1007/s00723-021-01335-0.

[44] M. Lee, M. C. Cassidy, C. Ramanathan, and C. M. Marcus. Decay of nuclear hyperpolarization in silicon microparticles. *Physical Review B - Condensed Matter and Materials Physics*, 84(3):33–35, 2011. ISSN 10980121. doi: 10.1103/PhysRevB.84.035304.

[45] P. E. Blöchl. Projector augmented-wave method. *Physical Review B*, 50(24):17953–17979, 1994. ISSN 01631829. doi: 10.1103/PhysRevB.50.17953.

[46] John P. Perdew, Matthias Ernzerhof, and Kieron Burke. Rationale for mixing exact exchange with density functional approximations. *Journal of Chemical Physics*, 105(22):9982–9985, 1996. ISSN 00219606. doi: 10.1063/1.472933.

[47] G. Feher. Electron spin resonance experiments on donors in silicon. I. Electronic structure of donors by the electron nuclear double resonance technique. *Physical Review*, 114(5):1219–1244, 1959. ISSN 0031899X. doi: 10.1103/PhysRev.114.1219.

[48] Michael W. Swift, Hartwin Peelaers, Sai Mu, John J.L. Morton, and Chris G. Van de Walle. First-principles calculations of hyperfine interaction, binding energy, and quadrupole coupling for shallow donors in silicon. *npj Computational Materials*, 6(1):1–9, 2020. ISSN 20573960. doi: 10.1038/s41524-020-00448-7. URL `http://dx.doi.org/10.1038/s41524-020-00448-7`.

[49] A Henstra, P Dirksen, and W. Th. Wenckebach. Enhanced dynamic nuclear polarization by the integrated solid effect. *Physics Letters A*, 134(2):134–136, 1988. ISSN 03759601. doi: 10.1016/0375-9601(88)90950-4. URL `http://linkinghub.elsevier.com/retrieve/pii/0375960188909504`.
